# Pediatric stewardship in Italy: a necessity, not an option - a National Multi-Society Expert Consensus on Antimicrobial and Diagnostic Stewardship (SIP, SITIP, SIMRI, SIAIP, SIMEUP, SIPPS, SICUPP, SIMIT, SIMPE, SIPINF, SIT, SIAATIP, SARNEPI, AIEOP, SIM, SITI, SIF, SIFACT, SITA, SIN)

**DOI:** 10.1186/s13052-025-02112-6

**Published:** 2025-10-09

**Authors:** Daniele Dona, Elisa Barbieri, Giulia Brigadoi, Martina Barchitta, Alberto Berardi, Samantha Bosis, Sara Buchini, Danilo Buonsenso, Andrea Cagliero, Beatrice Rita Campana, Fabio Capello, Romeo Carrozzo, Elio Castagnola, Salvatore Cazzato, Simone Cesaro, Elena Chiappini, Claudia Colomba, Manola Comar, Alessandra De Alessandri, Maia De Luca, Barnaba Esposito, Maria Rosaria Filograna, Alessia Franceschi, Luisa Galli, Silvia Garazzino, Fabrizio Gemmi, Emelyne Gres, Laura Lancella, Cecilia Liberati, Andrea Lo Vecchio, Milena Lo Giudice, Gianluigi Marseglia, Gaia Martelli, Daniele Mengato, Stefania Mercadante, Marianna Meschiari, Michele Miraglia del Giudice, Carlotta Montagnani, Paola Muggeo, Giangiacomo Nicolini, Stefania Nobili, Federico Pea, Dino Pedrotti, Lamberto Reggiani, Vittorio Sambri, Maurizio Sanguinetti, Alessandra Santiloni, Maria Chiara Silvani, Luisa Vatiero, Daniele Zama, Stefania Zampogna, Rosanna Zanai, Susanna Esposito

**Affiliations:** 1 Division of Pediatric Infectious Diseases, Department of Women’s and Children’s Health, University of Padua, 35128 Padua, Italy; 2 PENTA Foundation ETS, Padua, Italy; 3 Department of Medical and Surgical Sciences and Advanced Technologies “GF Ingrassia”, University of Catania, 95123 Catania, Italy; 4 Neonatal Intensive Care Unit, Department of Medical and Surgical Sciences for Mothers, Children and Adults, Policlinico University Hospital, Modena, Italy; 5 Pneumology and Infectious Diseases Unit, Fondazione IRCCS Ca’ Granda Ospedale Maggiore Policlinico, 20122 Milan, Italy; 6 Healthcare Professions Department, Institute for Maternal and Child Health, IRCCS ‘Burlo Garofolo’, Via Dell’Istria 65/1, Trieste, Italy; 7 Department of Woman and Child Health and Public Health, Fondazione Policlinico Universitario A. Gemelli IRCCS, Rome, Italy; 8 Area Pediatrica, Dipartimento Di Scienza Della Vita E Sanità Pubblica, Università Cattolica del Sacro Cuore, 00136 Rome, Italy; 9 Family Pediatrics, 10123 Turin, Italy; 10 Department of Medicine and Surgery, Pediatric Clinic, University Hospital, University of Parma, 43126 Parma, Italy; 11 UO Territorial Pediatrics, Primary Care Department, AUSL Bologna, 40126 Bologna, Italy; 12 Italian Primary Care Paediatrics Society (SICuPP), Lombardy, Italy; 13 Infectious Diseases Unit, Department of Pediatrics, IRCCS Istituto Giannina Gaslini, 16147 Genoa, Italy; 14 Pediatric Unit, Department of Mother and Child Health, Salesi Children’s Hospital, Ancona, Italy; 15 Pediatric Hematology Oncology, Department of Mother and Child, Azienda Ospedaliera Universitaria Integrata, Verona, Italy; 16 Department of Health Sciences, University of Florence, Viale Pieraccini, 24, 50100 Florence, Italy; 17 Pediatric Infectious Diseases Unit, Meyer Children’s Hospital IRCCS, Florence, Italy; 18 Division of Pediatric Infectious Diseases, “G. Di Cristina” Hospital, ARNAS Civico Di Cristina Benfratelli, University of Palermo, 90134 Palermo, Italy; 19 Institute for Maternal and Child Health-IRCCS Burlo Garofolo, 65/1 Via Dell’Istria, 34137 Trieste, Italy; 20 Department of Medicine, Surgery and Health Sciences, University of Trieste, Strada Di Fiume 447, 34149 Trieste, Italy; 21 Cystic Fibrosis Center, IRCCS G. Gaslini Institute, Genoa, Italy; 22 Infectious Diseases Unit, Bambino Gesù Children’s Hospital, IRCCS, 00165 Rome, Italy; 23 Federation of Italian Medical Pediatricians, Via Miglietta, 73100 Lecce, Italy; 24 G. Gaslini Children’s Hospital, Genoa, Italy; 25 Department of Paediatrics, Infectious Diseases Unit, Regina Margherita Children’s Hospital, University of Turin, Turin, Italy; 26 Regional Health Agency of Tuscany, Florence, Italy; 27 Department of Molecular Medicine, University of Padua, Padua, Italy; 28 Department of Translational Medical Sciences, Federico II University, Naples, Italy; 29 Family Care Paediatrician, Local Health Unit 6 Palermo, Palermo, Italy; 30 Department of Pediatrics, Ospedale San Matteo (IRCCS), Pediatrics Clinic, University of Pavia, Pavia, Italy; 31 Hospital Pharmacy Department, Azienda Ospedale-Università of Padova, Via Giustiniani 2, 35128 Padua, Italy; 32 Department of Infectious Diseases, Azienda Ospedaliero-Universitaria Di Modena, Policlinico Di Modena, University of Modena and Reggio Emilia, 41125 Modena, Italy; 33 Department of Woman, Child and of General and Specialized Surgery, University of Campania ‘Luigi Vanvitelli’, Naples, Italy; 34 Pediatric Hematology and Oncology, University Hospital Policlinico of Bari, Bari, Italy; 35 Pediatric Unit, San Martino Hospital, Belluno, Italy; 36 Department of Neuroscience, NEUROFARBA - Section of Pharmacology and Toxicology, University of Florence, Psychology, Drug Research and Child Health, Florence, Italy; 37 Department of Medical and Surgical Sciences, Alma Mater Studiorum, University of Bologna, Bologna, Italy; 38 Clinical Pharmacology Unit, Department for Integrated Infectious Risk Management, IRCCS Azienda Ospedaliero Universitaria Di Bologna, Bologna, Italy; 39 Anesthesiology, St. Chiara Hospital, Azienda Provinciale Per I Servizi Sanitari (APSS), Trento, ITA Italy; 40 Primary Care Pediatricians, Azienda Unità Sanitaria Locale (AUSL) Imola, 40026 Imola, Italy; 41 Unit of Microbiology, Greater Romagna Area Hub Laboratory, Cesena, Italy; 42 Dipartimento Di Scienze Di Laboratorio E Infettivologiche, Fondazione Policlinico Universitario A. Gemelli IRCCS, Rome, Italy; 43 Dipartimento Di Scienze Biotecnologiche Di Base, Cliniche Intensivologiche E Perioperatorie, Università Cattolica del Sacro Cuore, Rome, Italy; 44 Hospital Pharmacy Department Ravenna (AUSL Romagna), Ravenna, Italy; 45 Meyer Children’s Hospital IRCCS, Florence, Italy; 46 Pediatric Unit, IRCCS Azienda Ospedaliero-Universitaria Di Bologna, Via Massarenti 9, Bologna, Italy; 47 SIMEUP “Società Italiana Di Medicina Di Emergenza Ed Urgenza Pediatrica”, Pugliese Ciaccio Hospital, Catanzaro, Italy; 48 Cardiovascular Department, Mediterranean Pediatric Cardiology Center, Bambino Gesù Children’s Hospital, 98035 Taormina, Italy

## Abstract

Antimicrobial Stewardship Programs (ASPs) and Diagnostic Stewardship Programs (DSPs) are essential for optimizing infectious disease management and addressing antimicrobial resistance (AMR). However, the implementation of pediatric ASPs presents distinct challenges that set them apart from adult-focused initiatives. Additionally, many existing ASP guidelines are primarily tailored to the U.S. healthcare system, requiring significant adaptation to fit the diverse healthcare infrastructures, resources, and prescribing practices across different countries. These factors highlight the need for context-specific strategies to ensure the effective implementation of pediatric ASPs worldwide. To develop a national, intersociety consensus on pediatric ASPs in Italy, an ASP steering committee was established, bringing together a multidisciplinary group of experts. A systematic scoping review was conducted to identify relevant literature on ASPs and DSPs published between 2007 and August 2024, retrieving 260 articles. Based on this evidence, 33 recommendations were formulated, covering general ASP and DSP principles (10 recommendations), ASP interventions (14), DSP interventions (3), and monitoring strategies (6). Consensus on the importance and feasibility of each recommendation was reached using the Delphi method, with two rounds of anonymous questionnaires. The steering group defined a priori criteria for recommendation acceptance, requiring at least 80% agreement on the importance of each item. This consensus highlights the critical role of a multidisciplinary approach in ASP implementation, supported by institutional leadership. Given the variability in healthcare systems, ASP interventions must be tailored to specific settings, considering factors such as hospital resources, patient complexity, and the parent–child dynamic. Standardized metrics for assessing the impact of ASPs are essential for benchmarking and ensuring sustainability, although data collection remains a significant challenge. While there was strong agreement on the importance of the recommendations, feasibility assessments identified key areas requiring further refinement, particularly in settings with limited pediatric-specific expertise and diagnostic tools. This national consensus provides a structured framework for the implementation of pediatric ASPs in Italy, equipping clinicians with essential tools to optimize antibiotic use in both inpatient and outpatient settings. It represents a foundational step toward improving pediatric ASP, fostering national and international collaboration, and guiding future research to address implementation barriers.

**Supplementary Information:**

The online version contains supplementary material available at 10.1186/s13052-025-02112-6.

## Introduction

Antimicrobial resistance (AMR) continues to pose one of the most critical global health threats, with particularly severe implications for pediatric populations, especially those under 1 year of age, and for countries in Southern Europe [1–4]. The effective management of infectious diseases in children depends heavily on programs that promote the appropriate use of antibiotics and diagnostic tools. Antimicrobial stewardship programs (ASPs) are increasingly recognized as essential components in the fight against AMR, with their importance in pediatric healthcare gaining significant attention [5–10]. However, despite the growing awareness of AMR and its consequences, Italy has not yet developed a unified, evidence-based consensus on pediatric ASPs, offering an important opportunity to further refine the country’s response to this urgent health crisis.

A central goal of pediatric ASPs is to reduce inappropriate antibiotic use, which remains a persistent challenge. Children are among the most frequent recipients of antibiotics, particularly in outpatient settings, where unnecessary prescriptions and improper dosing are common [11–14]. Such misuse not only accelerates the development of antimicrobial resistance but also contributes to adverse drug reactions and long-term health issues, including disruptions to the developing microbiome and immune system. Particularly in early life, antibiotic exposure perturbs the developing microbiome and contributes to the observed rise in a variety of complex diseases mediated by the immune system, as well as metabolic disorders such as allergies, asthma, and obesity [14–18]. However, reducing inappropriate prescriptions in neonates presents an even greater concern [19]. While delayed antimicrobial treatment in this group can have devastating consequences, neonates, especially preterm neonates, often lack specific initial symptoms, and early diagnostic tests have limited predictive value.

Moreover, especially in outpatient settings, unnecessary antibiotic use can be substantially driven by the fear of being blamed for missing and not treating appropriately a severe bacterial infection, coupled with parental expectations and the pressure on healthcare providers to prescribe [20]. Additionally, pediatric-specific factors—including differences in pharmacokinetics, disease epidemiology, and the effects of antimicrobial exposure on the developing microbiome—require stewardship strategies tailored to children, rather than relying on adaptations of adult-focused approaches [21, 22].

The establishment of a national, intersociety consensus on pediatric ASPs represents a crucial step toward optimizing antibiotic use, reducing inappropriate prescriptions, and preventing the spread of resistant pathogens. Standardizing strategies across both hospital and primary care settings will improve clinical outcomes, minimize adverse drug events, and preserve the effectiveness of antimicrobials for future generations. Furthermore, a consensus-driven approach will promote interdisciplinary collaboration, ensuring that pediatricians, neonatologists, pediatric infectious disease specialists, microbiologists, pharmacists, nurses, and policymakers work together to develop and implement effective, evidence-based antimicrobial stewardship policies.

The recommendations provided by this consensus aim to equip clinicians with the necessary tools to advocate for the resources needed to support pediatric ASPs across inpatient and outpatient care settings in Italy, as well as in countries with similar health-care settings.

Moving forward, structured pediatric ASPs should no longer be viewed as optional but as integral components of high-quality, sustainable healthcare. This national consensus provides a framework for integrating antimicrobial stewardship into routine clinical practice. As Italy aligns with other countries in prioritizing pediatric antimicrobial stewardship, this initiative has the potential to serve as a model for international collaboration in combating AMR, ensuring the protection of children’s health for future generations. Additionally, it aims to address key areas crucial to children’s well-being that, while widely acknowledged as important, remain challenging to implement effectively within the country.

## Materials and methods

### Steering committee

In 2024, an Italian ASP steering committee was established to bring together a multidisciplinary group of experts in pediatric ASP. The group includes pediatricians (both hospital and primary care), pediatric infectious disease specialists, neonatologists, pediatric hemato-oncologists, microbiologists, hospital pharmacists, public health specialists, and nurses, all of whom play a pivotal role in incorporating antimicrobial stewardship principles into diverse healthcare settings. Panel members were selected through a direct invitation within each chair’s affiliated Italian scientific societies (Società Italiana di Pediatria, SIP, Società Italiana di Infettivologia Pediatrica, SITIP, Società Italiana Malattie Respiratorie Infantili, SIMRI, Società Italiana di Allergologia e Immunologia Pediatrica, SIAIP, Società Italiana di Medicina di Emergenza Urgenza Pediatrica, SIMEUP, Società Italiana di Pediatria Preventiva e Sociale, SIPPS, Società Italiana delle Cure Primarie Pediatriche, SICUPP, Società Italiana Medici Pediatri, SIMIT, Società Italiana di Malattie Infettive e Tropicali, SIMPE, Società Italiana di Pediatria Infermieristica, SIPINF, Società Italiana Telemedicina, SIT, Società Italiana di Anestesia Analgesia e Terapia Intensiva Pediatrica, SIAATIP, Società di Anestesia e Rianimazione Neonatale e Pediatrica Italiana, SARNEPI, Associazione italiana di ematologia e oncologia pediatrica, AIEOP, Società Italiana di Microbiologia, SIM, Società Italiana di Igiene Medicina Preventiva e Sanità Pubblica, SITI, Società Italiana di Farmacologia, SIF, Società Italiana di Farmacia Clinica e Terapia, SIFACT, Società Italiana di Terapia Antinfettiva Antibatterica Antivirale Antifungina, SITA, Società Italiana di Neonatologia, SIN).

This national, multidisciplinary panel was convened to advance pediatric antimicrobial stewardship policies, drawing on the collective expertise of professionals dedicated to optimizing antimicrobial use in children. By fostering collaboration among specialists with aligned expertise and objectives, the committee aims to develop standardized, evidence-based statements to strengthen ASP implementation across hospitals and primary care settings in Italy.

### Supporting literature review

A systematic scoping review was conducted to inform this consensus, aiming to identify the existing evidence on pediatric antimicrobial and diagnostic stewardship programs. The review followed the Preferred Reporting Items for Systematic Reviews and Meta-Analyses (PRISMA) guidelines. A comprehensive search was performed across the MEDLINE, Embase, and Cochrane Library databases, covering publications from January 1, 2007, to August 31, 2024, using a search strategy that combined Medical Subject Headings (MeSH) and free-text terms for ‘children’, ‘antimicrobial’, ‘diagnostic’ and ‘stewardship’. The completed search strategy is reported in Supplementary Material 1.

The steering group conducted the literature search, and studies were deemed eligible for full-text review if they met the following criteria: published in English, included data on patients under 18 years of age, and conducted in either outpatient or hospital settings. The review encompassed a variety of study designs, including randomized controlled trials, controlled and non-controlled before-and-after studies, controlled and non-controlled interrupted time series, and cohort studies.

### Consensus process for statements development

Consensus was reached through a Delphi method, a well-known process used in health care that involves several rounds of anonymous questionnaires.

The steering group worked collaboratively to develop a set of recommendations, drawing on a comprehensive literature review and the collective expertise of leading pediatric ASP specialists. These recommendations were then incorporated into an electronic survey using Google Forms and distributed via email to the targeted panelists, ensuring broad engagement from key stakeholders. Panelists were asked to evaluate anonymously all 33 recommendations based on their importance and feasibility using a five-point Likert scale (very important, important, unsure if important or unimportant, unimportant, very unimportant; very feasible, feasible, unsure if feasible or unfeasible, unfeasible, very unfeasible). Additionally, they had the option to list perceived barriers to implementation.

The steering group established a priori criteria for recommendation acceptance, requiring at least 80% agreement on the importance of each item (considering both"important"and"very important"responses). Survey responses were carefully reviewed, and any comments related to feasibility were documented and discussed.

A quantitative analysis of the statement ratings collected during the panel process was conducted to assess the level of consensus among participants. Following initial revisions, the results were presented and discussed in an online meeting chaired by the steering group, to facilitate further discussion and review of feedback received.

A second anonymous round of revised statements was then circulated, and final approval was obtained. Recommendations in the guidelines were categorized into four sections: General Principles, Antimicrobial Stewardship Interventions (including ancillary approaches), Diagnostics Stewardship Interventions, and Monitoring.

## Results

### Descriptions of the systematic review results

**Fig. 1:**
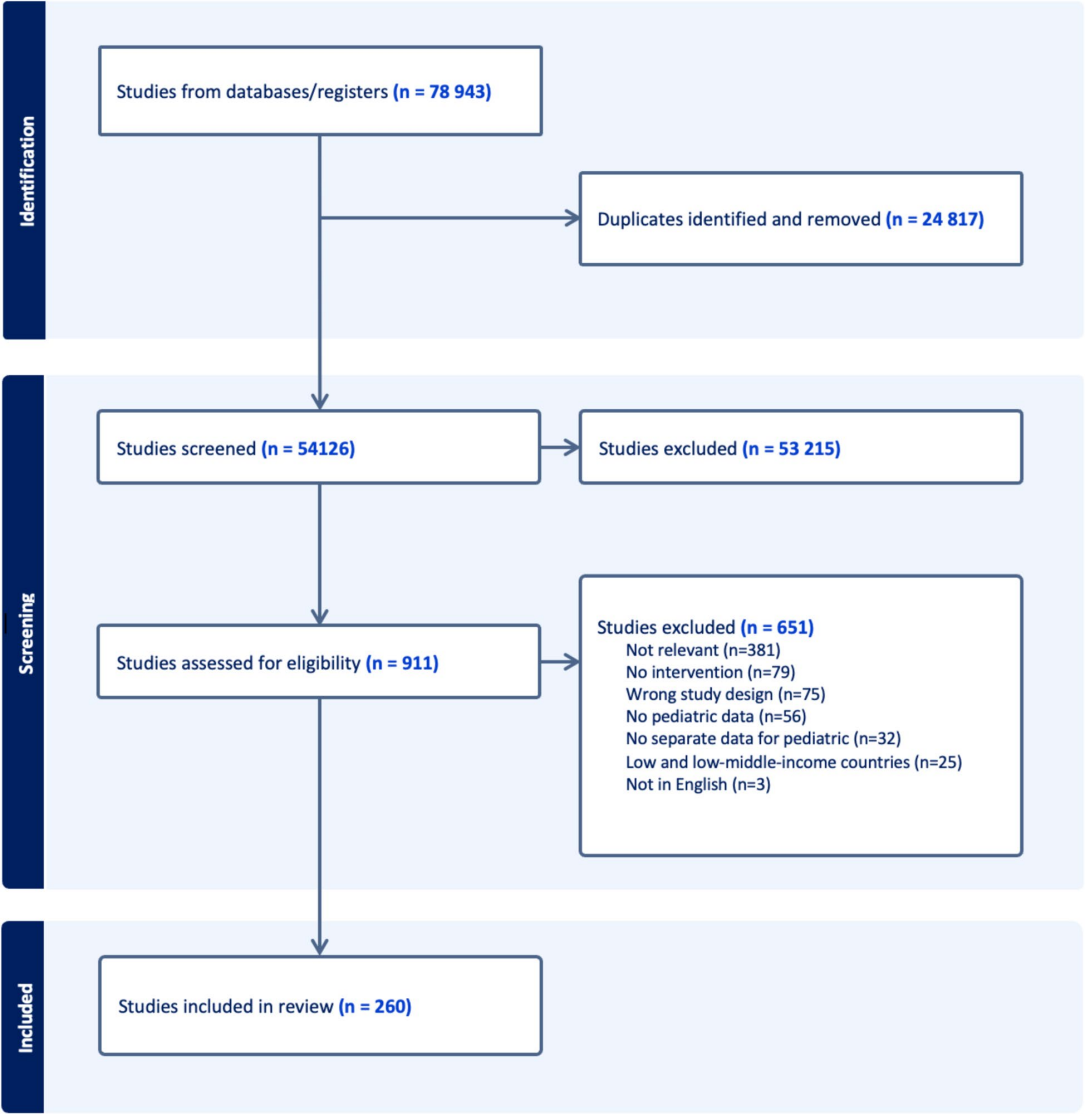
PRISMA flowchart of included studies

A total of 78,943 articles were retrieved from the systematic search across the three databases and uploaded on Rayyan software for screening. After removing duplicates, 54,126 studies remained for the title and abstract screening, of which 911 were assessed in full text. Following a thorough evaluation, 260 articles were included in the systematic review. The selection process is summarized in Fig. 1.

Most of the included studies were conducted in high-income countries, particularly in the USA and Europe. The majority focused on the implementation of ASPs in both inpatient and outpatient settings, with most published after 2015. In contrast, fewer articles addressed diagnostic stewardship interventions, with publications mainly appearing after 2018. The characteristics of the included articles are reported in Table S1.

### Statements

#### General principles

**Fig. 2:**
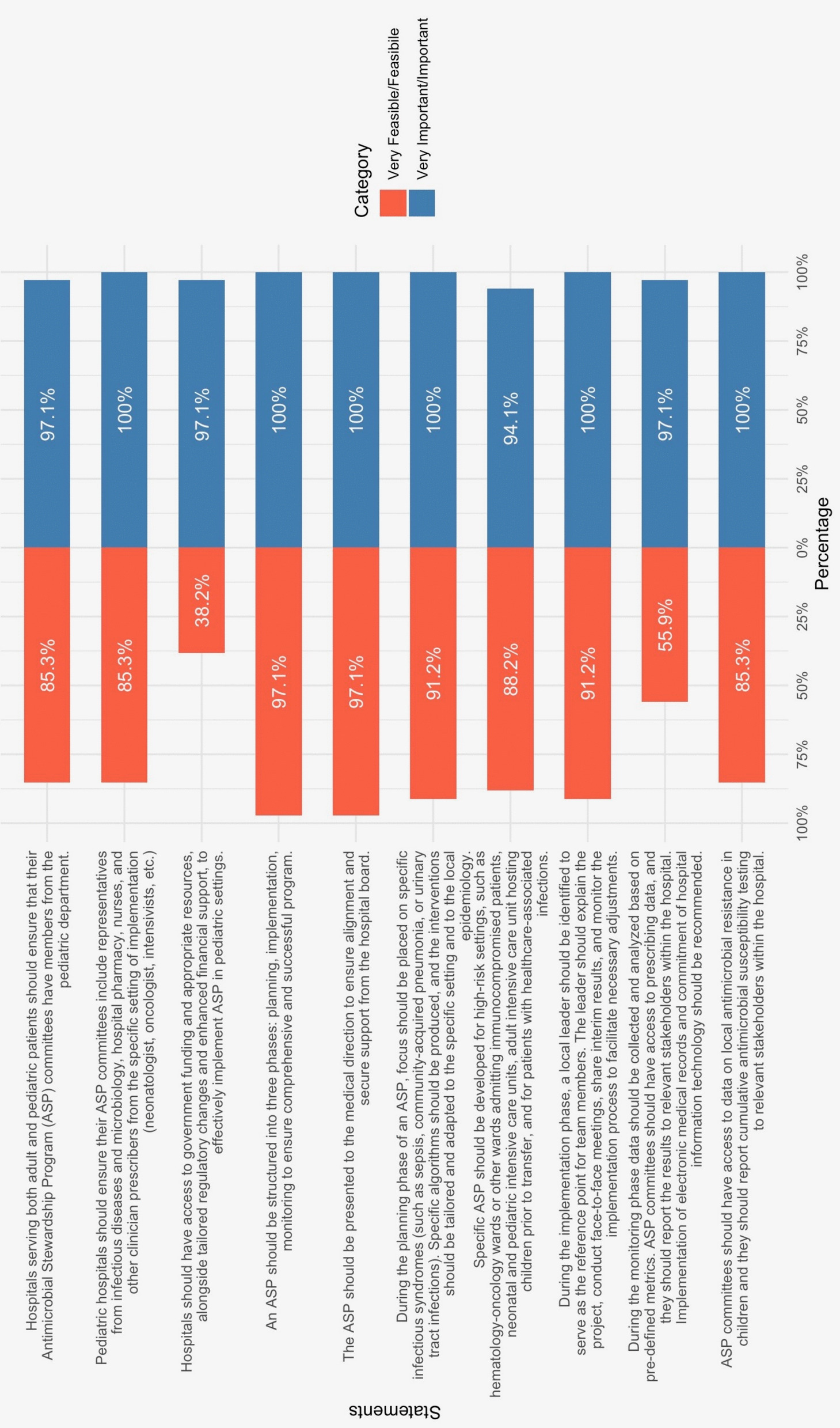
Tornado chart of results regarding feasibility and importance for general principles

The panel reached an overwhelming consensus (over 97%) on the ten key principles of pediatric ASP (Fig. 2), emphasizing the need for evidence-based approaches tailored to children’s unique physiological and developmental needs (*Importance 97.1%, Feasibility 85.3%*).

The successful implementation of pediatric ASPs requires a multidisciplinary approach, where effective leadership is a critical component. The roles within the team must be clearly defined to ensure optimal collaboration and coordination [5]. The Antimicrobial Stewardship Team (AST) should comprise professionals from diverse disciplines, including pediatric nurses, hospital pharmacists, microbiologists, public health specialists, and other relevant healthcare professionals [6] (*Importance 100%, Feasibility 85.3%*). In line with existing literature, the panel emphasizes the importance of direct engagement with prescribers from the specific clinical setting where the ASP is implemented, such as neonatologists, oncologists, intensivists, and others [23, 24]. This engagement facilitates meaningful interactions that help educate clinicians on best prescribing practices, foster collaboration in managing challenging cases, and address potential barriers encountered during program implementation. Moreover, the involvement of the clinical governance committee is essential to securing formal approval, ensuring institutional buy-in, and obtaining the financial support necessary for the program’s successful execution (*Importance 100%, Feasibility 97.1%*). Without the backing of clinical leadership and institutional resources, the long-term sustainability of pediatric ASPs faces a major impediment [5]. Implementing ASPs is particularly challenging in primary care and emergency departments due to short patient encounters, limited follow-up, high patient turnover, diagnostic uncertainty from limited rapid testing, and insufficient funding for outpatient care. Indeed, funding limitations are a challenge for both inpatient and outpatient ASPs, and government funding and appropriate resources should be available to support those programs (*Importance 97.1%, Feasibility 38.2%*).

Inpatient programs may benefit to a certain extent from centralized teams and shared resources, while outpatient ASPs face greater constraints. Resource shortages hinder the implementation of standardized protocols, tracking of clinical outcomes, and monitoring of improvements. The lack of dedicated funding is a major barrier, making it difficult to recruit specialized staff and sustain long-term stewardship efforts [25, 26].

In Italy, healthcare is primarily financed through public funds via the National Health Service (Servizio Sanitario Nazionale, SSN), which provides universal coverage largely free of charge. However, outpatient services and diagnostic testing may sometimes involve a co-payment, and there is no widespread use of private insurance or patient-paid subscription models for basic pediatric care. Given this structure, dedicated government funding remains essential to support sustainable ASPs in both inpatient and outpatient settings, to develop coordinated antimicrobial stewardship networks, enable structured collaboration across institutions, and to provide adequate compensation for professionals involved in stewardship activities.

The panel unanimously agreed that the implementation of pediatric ASPs should be carried out in three distinct phases: the planning phase, the implementation phase, and the monitoring and sustainability phase (*Importance 100%, Feasibility 97.1%*).

The planning phase is critical for establishing a robust program, ensuring that interventions are precisely targeted. This phase lays the foundation for the program by identifying specific goals, strategies, and outcomes (*Importance 100%, Feasibility 91.2%*). Moreover, the panel emphasized the need for tailored ASPs in high-risk settings, such as neonatal intensive care units (NICUs). In these environments, particularly for preterm infants, early antibiotic administration can be life-saving, yet clinical signs of infection often overlap with those of non-infectious conditions, making diagnosis challenging. Despite these complexities, recent studies have demonstrated the efficacy and safety of ASPs in this setting [27, 28].

Additionally, growing evidence links prolonged antibiotic use in uninfected preterm neonates to an increased risk of short-term adverse effects, including necrotizing enterocolitis, late-onset sepsis, bronchopulmonary dysplasia, and death [29–31]. Further challenges were identified, particularly in adult wards that also accommodate pediatric patients (*Importance 94.1%, Feasibility 88.2%*). Children present with a distinct spectrum of diseases, epidemiology, and risk factors compared to adults [32]. They have unique physiological and emotional responses, and often struggle to clearly communicate their needs and symptoms [33]. This can lead to frustration for both the children and the healthcare providers caring for them. Adult wards are generally not designed to be family-centered and are rarely equipped to accommodate both the child and their caregivers [33, 34]. Additionally, antibiotic prescribing in children presents specific complexities: not all antibiotics approved for adults are suitable for pediatric use, and dosage calculations based on weight (mg/kg) introduce a greater risk of prescribing errors — a concern even within dedicated pediatric settings [35]. The implementation phase focuses on performing the planned strategies. During this phase, the team implements the interventions and engages key stakeholders to ensure effective execution [5, 36] (*Importance 100%, Feasibility 91.2%*). The monitoring and sustainability phase is essential for evaluating the results and ensuring the long-term success of the program [37, 38]. Continuous data collection, whether manually, electronically, or through point prevalence surveys (PPSs), allows for comprehensive analysis using appropriate metrics. This phase ensures that the program can adapt and sustain improvements over time [5, 9, 36].

While the first two phases were deemed highly feasible, the panel raised concerns regarding data collection in the third phase (*Importance 97.1%, Feasibility 55.9%*). Data collection remains a major challenge. In the absence of integrated electronic medical record systems, manual collection of antibiotic prescription data becomes necessary. This process is labor-intensive and time-consuming, making it difficult to continuously assess prescribing patterns and effectively monitor progress.

The panel unanimously agreed on the critical importance of ensuring that ASP committees have access to local AMR data for the pediatric population and regularly report susceptibility rates to relevant hospital stakeholders to enable data-driven decisions and the optimization of guidelines and empirical antibiotic therapy. In this case, the feasibility of this measure was widely acknowledged (*Importance 100%, Feasibility 85.3%*). Ensuring that ASP committees have timely access to resistance trends also facilitates real-time intervention in high-risk clinical scenarios, ultimately improving patient outcomes and enhancing the sustainability of stewardship efforts [39–41].

#### ASP interventions

**Fig. 3:**
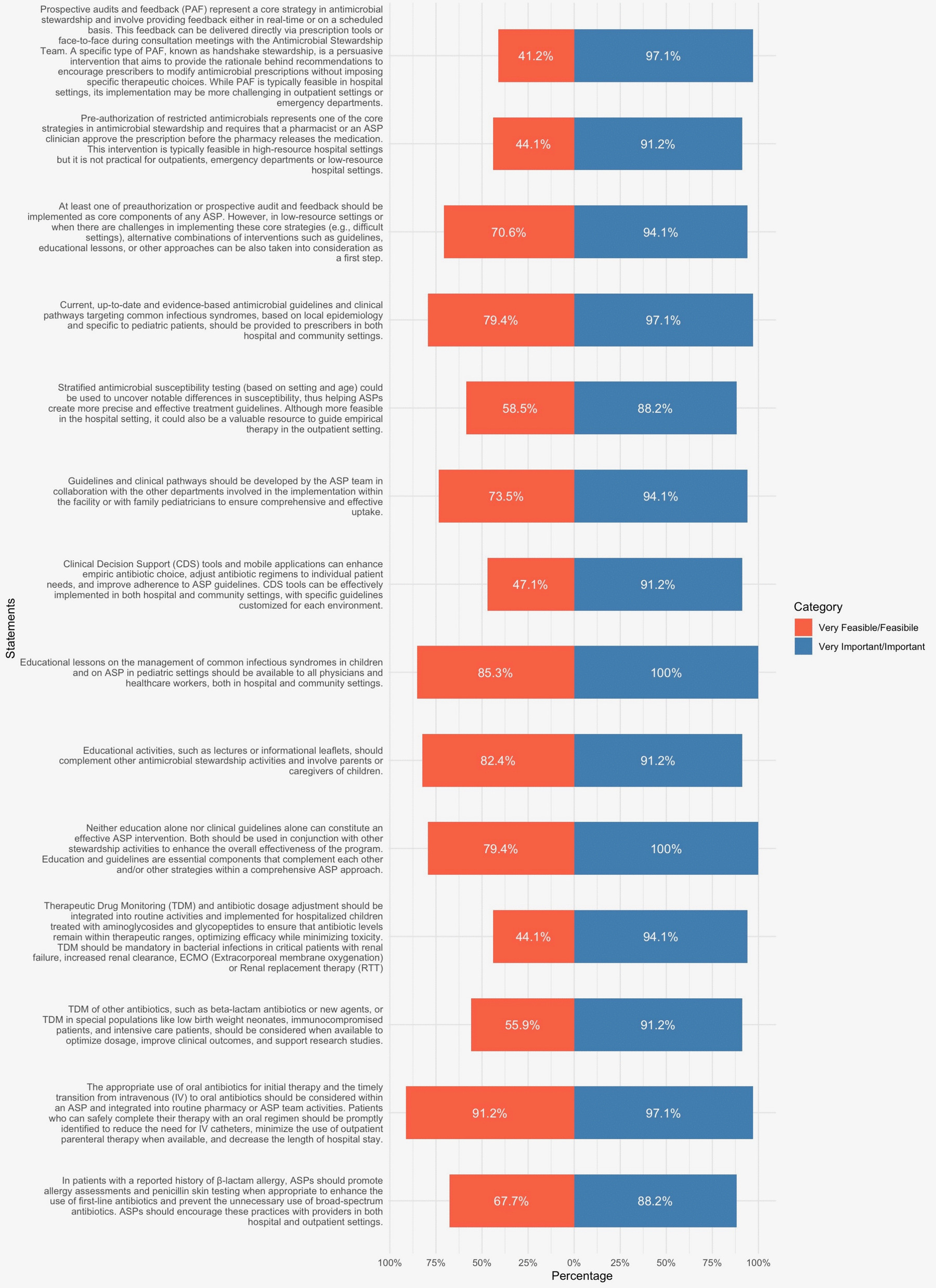
Tornado chart of results regarding feasibility and importance for asp interventions

The panel strongly endorsed the importance of antimicrobial stewardship interventions, with most statements receiving over 90% support for being important or very important. However, feasibility varied significantly (Fig. 3).

Core strategies like preauthorization of antimicrobials and prospective audit and feedback (PAF) were rated as highly important (*Importance 91.2% and 97.1%, respectively*) but had the lowest feasibility scores (*Feasibility 41.2% and 44.1%, respectively*), reflecting challenges in implementation, especially in outpatient and low-resource settings. In contrast, interventions such as education, guideline implementation, and timely IV-to-oral antibiotic transition were both highly important and more feasible. Ancillary approaches like Therapeutic Drug Monitoring (TDM) were considered important but had moderate feasibility (*94.1%−97.1% and 44.1%−55.9% respectively*) (Fig. 3).

Although both pre-authorization and PAF are considered core strategies for ASPs, their implementation presents significant challenges, particularly in primary care, ED or resource-limited settings when not endorsed at the legislative level. Pre-authorization of restricted antimicrobials provides direct control over prescribing by requiring approval from a pharmacist or ASP clinician before a drug is dispensed. While effective in optimizing empirical therapy, this approach reduces prescriber autonomy and may delay treatment. Its feasibility is generally limited to broad-spectrum antibiotics in the hospital setting [5, 6, 39] (*Importance 97.1%, Feasibility 41.2%*). PAF, including its variation known as handshake stewardship, offers a more flexible approach by providing prescribers with real-time or scheduled feedback on antimicrobial use, preserving clinical autonomy and promoting collaboration with the ASP team (*Importance 91.2%, Feasibility 44.1%*) [40, 42–44]. However, the success of these types of interventions depends on the availability of dedicated personnel, structured monitoring systems, and reliable prescription tracking tools—resources that are often scarce in outpatient and ED settings. Therefore, in low-resource settings or when there are challenges in implementing the core strategies, alternative combinations of easier interventions should be considered (*Importance 94.1%, Feasibility 70.6%*).

Education and clinical guidelines are crucial for ASP, but they must be integrated with the core strategies for meaningful impact [45, 46]. Current, up-to-date and evidence based antimicrobial guidelines should be provided in both hospital and community settings (*Importance 97.1%, Feasibility 79.4%*). Locally adapted clinical guidelines and pathways are essential for optimizing prescribing practices, provided they are developed collaboratively and aligned with regional epidemiology [47–49] (*Importance 94.1%, Feasibility 73.5%*). Educational talks play a crucial role in the success of ASP (*Importance 100%, Feasibility 85.35%*). Stratified antimicrobial susceptibility testing, accounting for both setting (inpatient or outpatient settings, and type of ward) and age, is crucial for identifying significant variations in susceptibility patterns and refining ASP-driven treatment guidelines. While more readily applicable in hospital settings, it can also help guide empirical therapy in outpatient care. However, implementing cumulative antibiograms, including weighted-incidence syndrome combination antibiograms (WISCA), presents significant challenges in data processing, limiting their broader feasibility despite their potential importance [40, 41] (*Importance 88.2%, Feasibility 58.5%*). In line with World Health Organization (WHO) and IDSA/SHEA recommendations, the implementation of clinical guidelines should be accompanied by targeted strategies such as structured education, clinical pathways development, and audit and feedback mechanisms (*Importance 100%, Feasibility 79.4%*).

Engaging patients and families through education is essential for reducing unnecessary antibiotic use and improving adherence to ASP [50] (*Importance 91.2%, Feasibility 82.4%*). Parental anxiety, pressure to prescribe, fear, diagnostic uncertainty, perceived risks, and communication challenges influence prescribing behavior, especially in outpatient settings. Public awareness campaigns, especially with multilingual materials, targeted education, a strong parent–healthcare provider communication, can help address these concerns, reduce over-prescription, and promote rational antibiotic use [20, 51, 52].

Computerized decision support (CDS) tools improve prescribing [53] but face barriers like cost, alert fatigue, and integration with existing healthcare medical record systems (*Importance 91.2%, Feasibility 47.1%*).

The panel strongly agreed on the critical role of TDM in the optimization of antibiotic dosing [54, 55], but its feasibility remains limited due to resource constraints and the need for specialized personnel (*Importance 94.1%, Feasibility 44.1%* for TDM regarding glycopeptides and aminoglycosides, *Importance 91.2% and Feasibility 55.9%* for further antibiotics). Another ancillary approach widely recognized as important and feasible regards the transition from IV to oral antibiotics (*Importance 97.1%, Feasibility 91.2%*). Encouraging the transition improves outcomes, reduces costs, and is a practical intervention within ASP [6]. Finally, addressing β-lactam allergies can further prevent unnecessary broad-spectrum antibiotic use [56], though challenges such as specialized personnel shortages and coordination difficulties hinder implementation (*Importance 88.2%, Feasibility 67.7%*).

#### Diagnostic stewardship

**Fig. 4:**
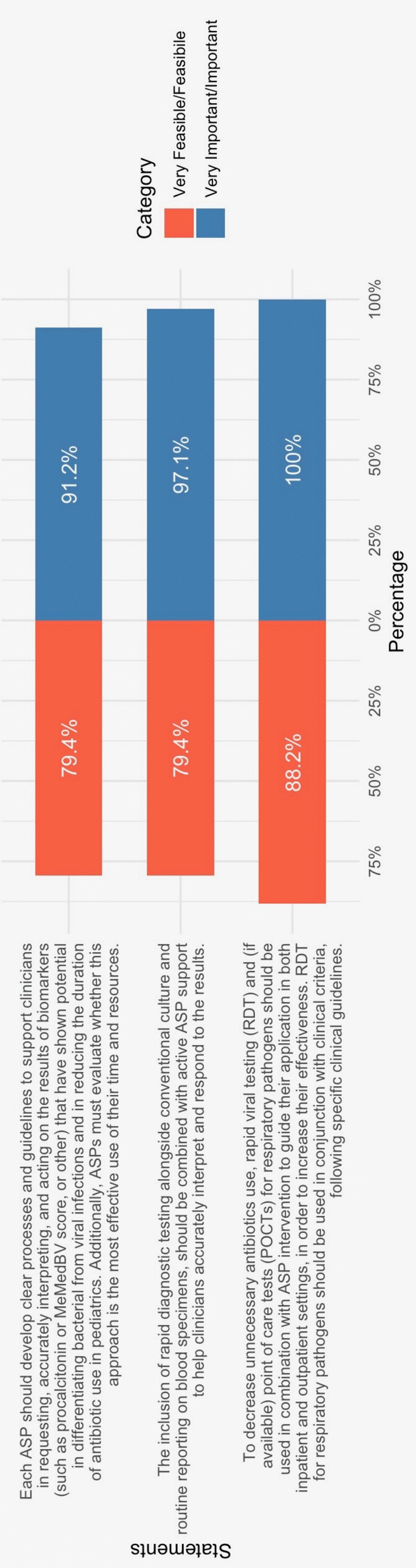
Tornado chart of results regarding feasibility and importance for dsp interventions

Based on the available literature, the panel reached a strong consensus on key aspects of diagnostic stewardship programs (Fig. 4).

The highest level of agreement was observed regarding the role of rapid diagnostic tests (RDTs). To reduce unnecessary antibiotic prescriptions, RDTs and, where available, point-of-care tests (POCTs) should be integrated into ASP strategies across both inpatient and outpatient settings to enhance their impact [57–60]. However, in pediatrics, further cost-effectiveness studies are needed to determine their optimal use. Additionally, RDT should always be interpreted alongside clinical criteria and culture results, when available, in accordance with established guidelines (*Importance 100%, Feasibility 88.2%*).

The integration of rapid diagnostic testing with conventional culture methods for blood specimens should be systematically supported by ASPs to aid clinicians in accurate interpretation and clinical decision-making (*Importance 97.1%, Feasibility 79.4%).*

Each ASP should establish clear protocols for using diagnostic tools to differentiate bacterial from viral infections and prevent overtesting and inappropriate antibiotic use [61] (*Importance 91.2%, Feasibility 79.4%*). Robust microbiological support with pediatric expertise, evidence-based pathways for common pediatric infections, and targeted education for clinicians and nurses are essential. Integrating these efforts with ASP interventions ensures proper specimen collection, timely processing, and accurate diagnostics, leading to appropriate diagnoses and cost-effective reductions in antibiotic use.

#### Monitoring

**Fig. 5:**
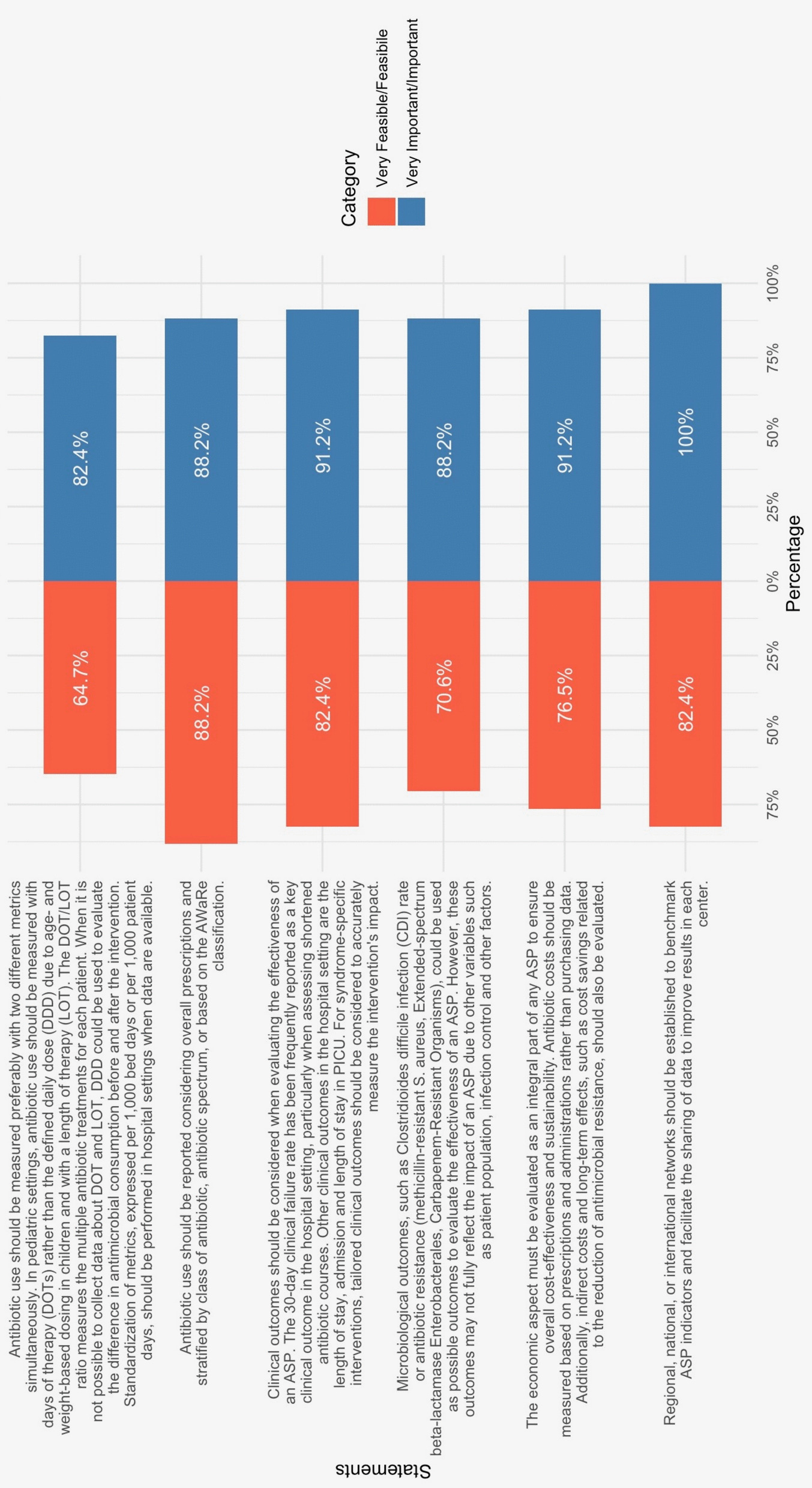
Tornado chart of results regarding feasibility and importance for monitoring

The panel reached a broad consensus on the necessity of measuring performance indicators to assess the effectiveness of ASPs (*Importance 82.4%, Feasibility 64.7%*) (Fig. 5). The discussion highlighted the variability observed in the literature regarding metrics selection. Defined daily doses (DDD) per 1,000 patient days can be readily extracted from pharmacy data in both outpatient and inpatient settings. However, other metrics, such as days of therapy (DOT) and length of therapy (LOT), may better represent antibiotic consumption in the pediatric population due to age- and weight-based dosing. Indeed, DOT and LOT per 1,000 bed days or 1,000 patient days are the preferred metrics for their consistency across studies, but their collection requires access to detailed medical records, which can be challenging, especially in healthcare settings without electronic medical records. Moreover, their application in outpatient settings remains particularly difficult due to data collection constraints, posing significant feasibility challenges in many healthcare systems [46].

Moreover, these metrics alone do not capture prescribing appropriateness, including dose accuracy, antibiotic spectrum, and clinical rationale for prescription. Given these limitations, the panel strongly supported the incorporation of the WHO AWaRe classification as a complementary tool for evaluating antibiotic use. This system categorizes antibiotics into *Access*, *Watch*, and *Reserve* groups based on their clinical importance and resistance potential, providing a structured framework for stewardship [52, 62] (*Importance 88.2%, Feasibility 88.2%*).

Antibiotic consumption metrics alone should not be the sole focus. It is equally crucial to evaluate clinical outcomes, particularly the rate of treatment failure at 30 days, as this provides a more comprehensive picture of the effectiveness of antibiotic stewardship interventions (*Importance 91.2%, Feasibility 82.4%*). Assessing both antibiotic consumption and clinical outcomes ensures that efforts to reduce antibiotic use do not compromise patient care and treatment success, allowing for a balanced approach to both stewardship and therapeutic efficacy.

From a microbiological perspective, ASP effectiveness can be assessed through infection- and resistance-related outcomes, such as *Clostridioides difficile* infection (CDI) rates or resistance trends in key pathogens, including methicillin-resistant *Staphylococcus aureus* (MRSA), extended-spectrum beta-lactamase (ESBL)-producing *Enterobacterales*, and carbapenem-resistant organisms [63, 64]. However, these indicators may not fully reflect ASP impact, as they are influenced by multiple confounding factors, including infection prevention strategies, patient demographics, and local epidemiology (*Importance 88.2%, Feasibility 70.6%*).

The economic impact of ASPs is another crucial factor requiring systematic evaluation to ensure long-term sustainability. Antibiotic costs should be assessed based on actual prescriptions and administrations rather than purchasing data alone, as inventory-based evaluations may not accurately reflect usage patterns. Additionally, cost analyses should incorporate indirect benefits, such as reductions in AMR and HAI, which can yield significant long-term savings [65–67] (*Importance 91.2%, Feasibility 76.5%*). However, even if needed in support of ASP implementation, comprehensive economic evaluations remain complex due to the challenges of modeling resistance-related costs and the heterogeneity of healthcare system structures. Nevertheless, such evaluations are essential for benchmarking, as they provide valuable data to compare the effectiveness and cost-efficiency of ASPs across different settings and over time (*Importance 100%, Feasibility 82.4%*).

## Discussion

This consensus represents the first comprehensive effort to establish ASP principles specifically for pediatric patients in Italy, providing valuable insights adaptable to other countries with similar healthcare systems. It lays a crucial foundation for future implementation and research in this field, aiming to improve pediatric care and foster national and international collaboration. While all statements received strong agreement regarding their importance from the initial round, differences in feasibility assessments highlight areas for improvement.

Accurate information is fundamental to ASP success, but data access remains a significant challenge. Real-time electronic surveillance provides a precise and representative overview of antibiotic use, facilitating the identification of prescribing trends before and after interventions. However, with the lack of the necessary electronic systems, manual data collection is still required. To mitigate this burden on healthcare professionals, PPS conducted periodically throughout the year serves as a practical alternative for tracking antibiotic prescriptions [68].

The use of heterogeneous metrics and the absence of internationally validated pediatric-specific measures complicate cross-study comparisons. DDDs have been utilized in pediatric research, but concerns persist regarding their applicability [69]. The WHO AWaRe classification offers a step forward in standardizing antibiotic use assessment [12, 52, 62].

One of the major obstacles in implementing pediatric ASPs is the need for regulatory adaptations, as existing guidelines and policies may not always align with the unique characteristics and requirements of pediatric care. Moreover, many existing guidelines are tailored to the U.S. healthcare system, necessitating adjustments to fit diverse global healthcare structures [51]. Some nations have already developed national ASP frameworks. For instance, the UK has implemented a range of ASP initiatives since 2013, focusing on reducing the use of broad-spectrum antibiotics in both primary and secondary care [70].

Beyond infrastructural limitations, financial constraints were a key concern identified by the panel. Sustainable ASP development requires secure funding, supported by national and regional healthcare commitments [5, 9].

Although ASPs are consistently associated with improved clinical practices and patient outcomes, their financial return on investment is often indirect and not immediately evident. Ensuring program sustainability requires strategic planning, including integrating stewardship responsibilities into existing clinical roles to optimize resource use. Nonetheless, for long-term success, securing dedicated financial support for personnel and infrastructure remains essential. Overcoming these barriers may involve leveraging innovative approaches such as telehealth and artificial intelligence (AI) to extend ASP reach and effectiveness.

AI holds significant promise in antimicrobial stewardship. Machine learning (ML) algorithms can support antibiotic prescribing by integrating real-time clinical data, aiding in treatment optimization. AI models can also predict AMR trends, guide therapeutic choices, and identify genetic markers associated with resistance [71]. For instance, an ML-based model developed in Greece using antimicrobial susceptibility data demonstrated 72.6% accuracy in predicting resistance patterns [72]. Still, translating AI tools into routine clinical practice requires further validation and integration into existing healthcare workflows. Similarly, telehealth can bridge critical gaps in ASP implementation, particularly in resource-limited or geographically remote areas. Virtual consultations enable healthcare providers to offer timely stewardship guidance, mitigating staffing shortages [73]. Additionally, digital tools that provide real-time data on antibiotic use—including alerts for broad-spectrum antibiotic prescriptions, automatic stop orders, and IV-to-oral conversion prompts—enhance ASP efficiency. Integrating AI and telehealth into pediatric care offers a promising pathway to improving antibiotic prescribing, strengthening adherence to clinical guidelines, and facilitating timely therapy adjustments.

Rapid diagnostic tools (POCTs, multiplex PCR, and biomarker assays) are pivotal in optimizing antimicrobial use in pediatric settings [57, 58, 74]. The successful implementation of POCTs requires not only initial investment but also sustained funding, adequate training for healthcare professionals, and integration within existing clinical workflows [60, 75]. Without proper interpretation, these diagnostics could lead to no improvement or unnecessary treatments, highlighting the need for structured ASP guidance [76]. Additionally, challenges such as the risk of ototoxicity with aminoglycosides in neonates highlight the need for genetic screening tools to guide safe prescribing in vulnerable populations [77].

Unlike adult populations, children often have limited access to novel antimicrobials due to ethical constraints and the complexity of pediatric drug trials [78]. This can lead to suboptimal dosing strategies, as pharmacokinetics and pharmacodynamics differ significantly among neonates, infants, older children and adults. TDM plays a crucial role in optimizing antimicrobial therapy, given the narrow therapeutic window of many antibiotics used to treat MDR infections [55, 79]. Despite its importance, implementing TDM in pediatric settings remains challenging due to limited specialized personnel, restricted access to real-time TDM services, and gaps in pediatric-specific pharmacokinetic data [80]. Overcoming these barriers requires investment in training, expanded access to TDM, and further research to refine pediatric dosing strategies.

This consensus represents a significant milestone, as it is the first time multiple Italian scientific societies have collaborated to define a unified approach to ASP and DSP in pediatric care. The statements were developed through an extensive literature review and critically evaluated by a panel of experts with diverse backgrounds, ensuring a comprehensive and multidisciplinary perspective.

A key strength of this consensus is its broad applicability across different clinical settings, encompassing both inpatient and outpatient care. This holistic approach acknowledges the complexity of ASP implementation across various healthcare environments, where prescribing practices, resource availability, and infrastructure differ significantly. Despite the high level of agreement on the importance of the statements, the panel recognized variability in their feasibility, reflecting the diverse challenges encountered in different settings.

Furthermore, the systematic scoping review provided the foundation for the evidence-based recommendations, strengthening the consensus and offering a robust framework for improving pediatric ASP strategies. By integrating multiple expertise, the consensus ensures that the proposed interventions align with current best practices and address critical gaps in pediatric antimicrobial use.

A limitation of this consensus is its primary focus on high-income healthcare settings, which may limit the direct applicability of its recommendations in resource-limited environments. While designed for broad implementation across various healthcare settings, practical feasibility may vary depending on institutional policies, local regulations, and healthcare system structures. Future research should assess the adaptability of these recommendations in diverse contexts, ensuring their relevance across different economic and epidemiological landscapes.

## Conclusions

This document represents the first consensus-based framework for pediatric ASPs in Italy, offering a structured approach to optimizing antimicrobial use in children and offering valuable insights for developing ASPs in other countries with similar healthcare systems.

This initiative fosters interdisciplinary collaboration, facilitating knowledge exchange while promoting future research in pediatric antimicrobial stewardship.

Despite the strong consensus on the importance of these recommendations, significant challenges remain in their practical implementation. Variability in healthcare infrastructure, resource availability, and institutional policies may impact the feasibility of certain interventions, particularly in settings with limited access to pediatric-specific expertise and diagnostic tools. Addressing these barriers should be a priority for future research to ensure effective implementation and optimize antibiotic use in pediatric healthcare.

Strengthening pediatric ASPs will be crucial for safeguarding antimicrobial efficacy, improving pediatric healthcare quality, and ensuring that children worldwide benefit from responsible and effective antibiotic use.

## Supplementary Information

Supplementary Material 1.

## Data Availability

All the data are included in the manuscript.
