# Supplementary material for: Pediatric stewardship in Italy: a necessity, not an option - a National Multi-Society Expert Consensus on Antimicrobial and Diagnostic Stewardship (SIP, SITIP, SIMRI, SIAIP, SIMEUP, SIPPS, SICUPP, SIMIT, SIMPE, SIPINF, SIT, SIAATIP, SARNEPI, AIEOP, SIM, SITI, SIF, SIFACT, SITA, SIN)
Source: Ital J Pediatr. 2025 Oct 9;51:283. doi: 10.1186/s13052-025-02112-6 (PMC12512260; doi:10.1186/s13052-025-02112-6)
Supplement: Supplementary file 1 — Supplementary Material 1. [file 13052_2025_2112_MOESM1_ESM.docx]

**Search strategy:**

1. (newborn* or neonat* or infan* or toddler* or pre-schooler* or preschooler* or child* OR children or adolescen* or pediatr* or paediatr* or youth* or teen or teens or teenage* or kid or kids or baby or babies).mp.
2. guideline* .mp.
3. (antibiotic stewardship or antimicrobial stewardship).mp.
4. diagnostic stewardship.mp.
5. (anti-bacterial* or antibacterial* or anti-mycobacterial* or antimycobacterial* or antibiotic* or anti-infective or antifungal or anti-fungal or bactericid* or bacteriocid* or antimicrobial* or treatment* or therap* or prophyla* or perioperative*).mp.
6. (adher* or complian* or concordan* or according*).mp.
7. exp "Outcome Assessment (Health Care)"/ or outcome*.mp.
8. exp Drug Resistance/ or resistan*.ti,ab,kw.
9. exp "Costs and Cost Analysis"/ or cost*.ti,ab,kw.
10. exp "Drug-Related Side Effects and Adverse Reactions"/ or adverse effects.fs. or (adverse effect* or adverse reaction* or adverse drug reaction* or adverse event* or adverse drug event* or undesirable effect* or side effect*).ti,ab,kw. or exp Mortality/ or mortality.fs. or (mortalit* or death* or fatal*).ti,ab,kw. or Morbidity/ or morbidit*.ti,ab,kw.
11. 3 or 4
12. 6 or 7 or 8 or 9 or 10
13. 5 and 12
14. 2 and 5
15. 11 or 14
16. 1 and 13 and 15
17. 16 not case reports.pt.
18. 17 not (exp HIV Infections/ or exp HIV/ or (HIV or human immunodeficiency virus).ti.)
19. Limit 18 to yr=”01.01.2007-31.08.2024”

**Table S1.** Characteristics of included studies

| **Author** | **Pubblication year** | **Title** | **Study design** | **Country** | **Setting** | **Type of ASP** | | **Main results** |
| --- | --- | --- | --- | --- | --- | --- | --- | --- |
| Andrade et al [77] | 2023 | Evaluation of pharmacist guided intervention using procalcitonin and respiratory virus testing | RCT | USA | ED | •• | POCT Flu/RSV Assay, PCT, and pharmacist-led result-based education for physicians | There was little difference in antibiotic or antiviral prescribing between the intervention and control groups in this study (39% - 32% = 7.0%, 95% CI: −6.2, 20.2, P=0.30). However, a post-hoc analysis of the use of PCT showed PCT results were used as indicated in the ED (P=0.001). |
| Angoulavant et al [78] | 2012 | Impact of implementing French antibiotic guidelines for acute respiartory-tract infections in a paediatric emergency department, 2005-2009 | BA | FRA | ED | •• | Local guideline based on French guidelines on antibiotic prescriptions for ARTI; teaching sessions twice a year; pocket cards | The proportion of ARTI patients given antibiotic prescriptions fell from 32.1% during the first year to 21% in year 4 (p<10−4, Cochran- Armitage test). Amoxicillin-clavulanic acid and amoxicillin accounted for 50% and 34% of antibiotic prescriptions for ARTI, respectively |
| Angoulavant et al [79] | 2014 | Impact of unlabeled French antibiotic guidelines on antibiotic prescriptions for acute respiratory tract infections in 7 Pediatric Emergency Departments, 2009-2012 | BA | FRA | ED | ••• | Local protocols for antibiotic use with ARTI, implementation of guidelines with scientific discussion, teaching lessons twice per year, pocket guidelines | Amoxicillin prescriptions rose from 34.0% to 84.7%, while amoxicillin clavulanate and cefpodoxime prescriptions decreased to 10.2% and 2.5%, respectively |
| Aronson et al [80] | 2015 | Association of Clinical Practice Guidelines with Emergency Department Management of Febrile Infants ≤ 56 Days | ObsR, cross sectional | USA | ED | • | Local Clinical Practice Guideline | Ceftriaxone use at ED discharge varied significantly based on guidelines recommendations. The guidelines implementation were not associated with lower healthcare costs |
| Baer et al [81] | 2013 | Procalcitonin Guidance to Reduce Antibiotic Treatment of Lower Respiratory Tract Infection in Children and Adolescents (ProPAED): A Randomized Controlled Trial | RCT | CHE | ED | *•* | Laboratory PCT | Antibiotic prescribing rates were not significantly different in PCT guided patients compared to controls (OR 1.26; 95% CI 0.81, 1.95). Mean duration of antibiotic exposure was reduced from 6.3 to 4.5 days under PCT guidance for all lower respiratory tract infection and from 9.1 to 5.7 days for pneumonia |
| Barbieri et al. [34] | 2020 | Impact and Sustainability of Antibiotic Stewardship in Pediatric Emergency Departments: Why Persistence Is the Key to Success. | BA | ITA | ED | •• | CPs were delivered as laminated pocket cards; educational lectures were presented to physicians and residents on how to implement these tools in practice but with different timing in the different settings | Broad-spectrum prescription rates decreased significantly by 80% for pharyngitis and 29.5 to 55.2% for otitis after the implementation. In Center C, rates gradually increased from the year after the implementation. Amoxicillin dosage adjusted to pharyngitis recommendations in Center C (53.7 vs. 51.6 mg/kg/die; p = 0.011) and otitis recommendations in Center A increasing from 50.0 to 75.0 mg/kg/die (p < 0.001). Days of therapy in children < 24 months with otitis increased from 8.0 to 10.0 in Center A, while in older children decreased in Center A (8.0 vs. 7.0; p < 0.001) and Center B (10.0 vs. 8.0; p < 0.001). |
| Bird et al [82] | 2021 | A Pragmatic Study to Evaluate the Use of a Rapid Diagnostic Test to Detect Group A Streptococcal Pharyngitis in Children With the Aim of Reducing Antibiotic Use in a UK Emergency Department. | BA | GBR | ED | • | Clinical scoring system and GAS RDT for sore throat | The baseline prescribing rate was 79%, whereas rates after intervention were 24% and 27%, respectively. |
| Crook et al [83] | 2020 | Impact of clinical guidance and rapid molecular pathogen detection on evaluation and outcomes of febrile or hypothermic infants | BA | USA | ED | •• | Clinical guideline during period 2 associated with Rapid testing during period 3 | Use of a standardized guideline safely reduced unnecessary testing and LPs, and use of mPCR tests together with the guideline further reduced unnecessary testing and decreased antibiotic exposure for infants 29-60 days old |
| Cunney et al [84] | 2019 | ‘Start smart’: using front-line ownership to improve the quality of empiric antibiotic prescribing in a paediatric hospital | ObsP | IRL | ED | ••• | Plan, Do, Study, Act, Weekly audit with feedback, front-line ownership, spot quiz, app and laminated card, poster | Guideline compliance increased from a median of 30% in December 2014 to 100% in March 2015 and was sustained at 100% to September 2016, then 90% to December 2017. The intervention was associated with an overall reduction in antimicrobial consumpions and a €105000 reduction in annual antimicrobial acquisition costs |
| Dagget et al. [85] | 2022 | Improving Emergency Department Use of Safety-Net Antibiotic Prescriptions for Acute Otitis Media | BA | USA | ED | •• | Safety-net antibiotic prescriptions in conjunction with parent education | The percentage offered a SNAP increased at both EDs. One ED had a single shift in the mean to 7.9%, whereas the other had 2 shifts in the mean, an initial shift to 5.1% and a second to 7.3%. Providers consistently used the algorithm and electronic medical record aids. |
| Demirjian A et al [86] | 2019 | Implementation of a Highly Accurate Rapid Point-of-Care Test for Group a Streptococcus Detection at a Large Pediatric Emergency Department in South London | ObsP | GBR | ED | •• | Algorithm describing criteria for testing and proposed treatment of suspected GAS tonsillitis with Rapid Point-of-Care Test for Group a Streptococcus Detection | Strong association between a molecular-based point-of-care testing result and outpatient antibiotic prescribing (P < 0.001). |
| Dona et al [87] | 2018 | The Impact of Clinical Pathways on Antibiotic Prescribing for Acute Otitis Media and Pharyngitis in the Emergency Department | BA | ITA | ED | •• | CPs were delivered as laminated pocket cards and 3 educational lectures were presented to physicians and residents on how to implement these tools in practice | There was an increase in “wait and see” approach for AOM and a decrease in broad-spectrum prescriptions for both AOM and GAS pharyngitis (53.2% vs. 32.4%; p < 0.001 - 46.4% vs. 6.6%; p < 0.001). For both conditions, no difference was found in treatment failure, and total antibiotics cost was significantly reduced, with a decrease especially in broad-spectrum antibiotics costs |
| Dube et al [88] | 2023 | Improving Prescribing for Otitis Media in a Pediatric Emergency Unit: A Quality Improvement Initiative. | ObsP | USA | ED | ••• | Guideline card, a 5-day amoxicillin prescription option, educational sessions, text templates for WSPs explanations, and reminder signs for appropriate prescribing, emails with performance graphs to reinforce engagement. | Our baseline data showed that only 39% of patients prescribed antibiotics were prescribed an appropriate duration based on age and estimated AOM severity, and only 3% were prescribed WSPs. Via 2 PDSA cycles, we increased the percentage of patients who received appropriate antibiotics to an average of 67%, sustained for >6 months |
| Free et al [89] | 2021 | Specialist pneumonia intervention nurse service improves pneumonia care and outcome | ObsP | GBR | ED | •• | SPIN service : assessment checklist based on the BTS pneumonia guidelines, face-to-face education modules | 82% of these admissions received antibiotic treatment in <4 hours (68.5% in the national audit). Compared with the pre-SPIN period, there was a significant reduction in both 30-day (OR=0.77 (0.70–0.85), p<0.0001) and in-hospital (OR=0.66 (0.60–0.73), p<0.0001) mortality after service implementation, with a review by the service showing the largest independent 30-day mortality benefit (HR=0.60 (0.53–0.67), p<0.0001). There was no change in length of stay (median 6 days). |
| Geurts et al [90] | 2014 | Impact analysis of an evidence-based guideline on diagnosis of urinary tract infection in infants and young children with unexplained fever | BA | NLD | ED | •• | Implementation of guideline, several group lectures in medical staff meetings, laminated pamphlets of the guideline were available at the ED, written instructions were sent threee times to all health care professionals working at the ED | Guidelines compliance increased (24.9 % pre vs 46.7 % post) |
| Gomez et al [91] | 2021 | Impact of the Step-by-Step on febrile infants. | BA | ESP | ED | •• | Step-by-Step approach on care quality in young febrile infants : two information training sessions + algorithm | Resource use: lumbar puncture and admission rates decreased (24.1% vs 18.7% and 43.6% vs 38.3%, respectively; p<0.01), while the rate of antibiotic therapy increased (30.2% vs 43.2%; p<0.01). |
| Grandjean-Blanchet et al [92] | 2023 | Value-Based Care for Healthy Children With First Episode of Febrile Neutropenia | BA | CAN | ED | •••• | Guideline for healthy children with febrile neutropenia, education, targeted audit and feedback, and reminders | Over the 44-month study period, the mean percentage of low-risk patients hospitalized and/or who received antibiotics decreased from 73.3% to 12.9%. Importantly, there were no missed serious bacterial infections, no new hematologic diagnoses after ED discharge, and only 2 ED return visits within 72 hours without adverse outcomes. |
| Hamner et al [93] | 2022 | Improving Duration of Antibiotics for Skin and Soft-tissue Infections in Pediatric Urgent Cares | ObsP | USA | ED | •• | Update on current guidelines, Modified the electronic health record to display antimicrobial prescription sentences from shortest to longest duration ,  Provided project outcome and balancing measure updates to UCC providers at regular intervals. | After completing our PDSA cycles, we found that the percentage of children receiving 5 to 7 days of oral antibiotics for SSTIs exceeded 85%. The improvement was sustained over multiple months. There was no increase in the proportion of patients returning to the UCCs with an SSTI diagnosis within 14 days. |
| Kooner et al [94] | 2024 | Reducing Antibiotic Duration for Uncomplicated Urinary Tract Infection in Pediatric Emergency Department | BA | USA | ED | •• | Education, practice feedback, and electronic health record changes | In 1292 (n = 363 baseline, 929 post-intervention) eligible patients treated for uUTI, shorter antibiotic duration increased from 13% to 91%. We met our 50% aim within 2 months, with continued improvement leading to an additional centerline shift. Consequently, 2619 antibiotic days were saved. |
| Malmgren et al [41] | 2019 | Education, decision support, feedback and a minor reward: a novel antimicrobial Stewardship intervention in a Swedish paediatric emergency setting | BA | SWE | ED | •• | Education, decision support, email based feedback and a physician-directed reward | The ASP significantly improved adherence to the practice of refraining from antimicrobial treatment when recommended by guidelines |
| McGonagle E.A. et al [50] | 2023 | Evaluation of an Antimicrobial Stewardship Decision Support for Pediatric Infections | RCT | USA | ED | • | CDS developed through user-centered design for antibiotic prescribing for pediatric CAP and UTI | Usability testing in 21 ED clinical providers demonstrated that, compared to the standard order sets, providers preferred the prototype CDS, with improvements in domains such as explanations of suggested antibiotic choices ( p  < 0.001) and provision of additional resources on antibiotic prescription ( p  < 0.001). Simulated use of the CDS also led to overall improved guideline-adherent prescribing, with a 31% improvement for CAP. |
| Mercurio et al [95] | 2020 | Clinical Practice Guideline Reduces Evaluation and Treatment for Febrile Infants 0 to 56 Days of Age | BA | USA | ED | •• | Education, training and adoption of clinical practice guideline | CPGs implemented with appropriate teaching and training staff can enable decreased testing, treatment, ad admission of febrile infants, particularly in their second month of life without change in 7-day readmission reate |
| Nedved A et al [96] | 2023 | Impact of an antibiotic stewardship program on antibiotic choice, dosing, and duration in pediatric urgent cares | BA | USA | ED | •• | Tracking and reporting, semi-annual educational sessions, outpatient ASP handbook, prescription folders for common diagnoses (such as AOM, and SSTIs) within the EHR, displayed antibiotic stewardship commitment letters based on the CDC's MITIGATE tool kit | The percentage of prescriptions with the recommend agent at the appropriate dose and duration increased from a mean of 32.7% to 52.4%. The most substantial changes were seen in antibiotic duration (63.2%-80.5%), and appropriate dose (64.6%-77%). |
| Nedved Aet al [97] | 2022 | A Multisite Collaborative to Decrease Inappropriate Antibiotics in Urgent Care Centers | ObsP | USA | ED | •• | Menu of publicly available antibiotic stewardship materials : MITIGATE Antimicrobial Stewardship Toolkit, provider clinical education, provider communication training, parent engagement, and patient engagement tools. | Overall inappropriate antibiotic prescription rates decreased by a relative 53.9%. Inappropriate antibiotic prescribing decreased from 57.0% to 36.6% for AOM, 54.6% to 48.4% for otitis media with effusion, and 66.9% to 11.7% for pharyngitis. |
| Otake S et al [98] | 2023 | Comparing the effects of antimicrobial stewardship at primary emergency centers | BA | JPN | ED | • • | Facility-specific guideline, monthly newsletter that summarized recent antimicrobial prescribing patterns and the facility's prescribing targets | The 3GCs prescription rate at Site A, Site B, and Site C decreased from 6.7%, 4.2%, and 6.1% in 2016 to 2.3%, 1.0%, and 2.0% in 2019, respectively. |
| Powell et al [99] | 2015 | Appropriate Use of Vancomycin in a Pediatric Emergency Department Through the Use of a Standardized Electronic Guideline | BA | USA | ED | ••• | Implementation of a standardized treatment guideline + Antibiotic order template + Individual chart audits | No statistical difference in utilization rates pre- and post-standardized treatment guideline and order template (4% vs 3%) |
| Rossin S et al [57] | 2021 | Multistep antimicrobial stewardship intervention on antibiotic prescriptions and treatment duration in children with pneumonia | BA | ITA | ED | • | CPs for lower respiratory tract infections : pocket cards / poster | A drastic reduction of broad-spectrum antibiotics prescription for inpatients has been noticed; from 100.0% in the PRE-period to 66.7% in POST1, and up to 38.5% in POST6. Simultaneously, an increase in amoxicillin use from 33.3% in the PRE-period to 76.1% in POST1 (p-value 0.078 and 0.018) has been seen. The outpatients’ group’s broad-spectrum antibiotics prescriptions decreased from 54.6% PRE to 17.4% in POST6. Both for outpatients and inpatients, there was a decrease of macrolides. The inpatient group’s antibiotic therapy duration decreased from 13.5 days (PRE-period) to 7.0 days in the POST6. Antibiotic therapy duration in the outpatient group decreased from 9.0 days (PRE) to 7.0 days (POST1), maintaining the same value in subsequent periods. |
| Shishido A et al [100] | 2021 | Effects of a nudge-based antimicrobial stewardship program in a pediatric primary emergency medical center | BA (ITS) | JPN | ED | • | Nudge-based ASP in reducing unnecessary 3GC : monthly newsletters that report current antimicrobial use patterns and prescribing targets. | The number of unnecessary 3GC prescriptions decreased by 67.2% in the year after ASP implementation. The interrupted time-series analysis showed that the ASP was significantly associated with a reduction in 3GC prescriptions (regression coefficient − 0.58, P < 0.001). |
| van de Maat et al [101] | 2021 | The influence of chest X-ray results on antibiotic prescription for childhood pneumonia in the emergency department | RCT | NLD | ED | • | CXR | Children who underwent CXR were more likely to receive antibiotics, also when adjusted for clinical signs and symptoms, hospital and CXR result (OR 7.25 [95% CI 2.48–21.2]) |
| van de Maat et al [102] | 2020 | Evaluation of a clinical decision rule to guide antibiotic prescription in children with suspected lower respiratory tract infection in The Netherlands: A stepped-wedge cluster randomised trial | stepped wedge cluster RT | NLD | ED | *•* | Validated clinical prediction model (Feverkidstool) | Clinical decision rule for childhood pneumonia did not reduce overall antibiotic prescription, but it was non-inferior to usual care and leads to fewer strategy failures and fewer antibioticrs prescribed in low/intermediat-risk children , suggesting improved targeting of antibiotic by the decision rule |
| Walters et al [103] | 2019 | An Ambulatory Antimicrobial Stewardship Initiative to Improve Diagnosis and Treatment of Urinary Tract Infections in Children | BA | USA | ED | *•••* | PDSA cycles | After project initation, 100% of all antibiotic presciptions for UTI were guideline-concordant. These changes have been sustained for 19 months since the initiative began. |
| Weddle et al [104] | 2013 | Impact of an Educational Intervention to Improve Antibiotic Prescribing for Nurse Practitioners in a Pediatric Urgent Care Center | BA | USA | ED | *•* | Educational sessions | The rate of inappropriate antibiotic use among all conditions was 10% before and 8% after the intervention. A decrease in inappropriate antibiotic prescribing was seen after the educational session |
| Williams, Derek et al [105] | 2023 | Antibiotic clinical decision support for pneumonia in the ED: A randomized trial. | RCT | USA | ED | • | Electronic health record-based CDS : antibiotic recommendations tailored to each encounter and in accordance with national guidelines. | Exclusive guideline-concordant prescribing did not differ at 24 h (CDS, 51.7% vs. usual care, 53.3%). In pre-specified stratified analyses, CDS was associated with guideline-concordant prescribing among encounters discharged from the ED (OR 1.53 [95% CI: 1.01, 2.33]), but not among hospitalized encounters. Mean time to first antibiotic was shorter in the CDS group (p = .024). |
| Ambroggio et al [106] | 2013 | Quality Improvement Methods Increase Appropriate Antibiotic Prescribing for Childhood Pneumonia | BA | USA | ED + inpatient | *•••* | Multiple PDSA:1) guideline seminar, grand rounds, antibiotic reccomendations in the medical staff update, 2) charge nurse flag cards, 3) index card with approppriate first-line antibiotic information for ED physicians and inpatient residents and resident report, 4) history and physical examination templetae and order set in EMR and link to PIDS/IDSA guideline | Appropriate first-line prescription for CAP increased from of 0% to 100% at the ED and on the hospital medicine resident teams from 30% to 100% |
| Dona et al [107] | 2018 | Effects of clinical pathway implementation on antibiotic prescriptions for pediatric community-acquired pneumonia | BA | ITA | ED + inpatient | •• | Clinical pathway for CAP and educational sessions | Overall, there was a decrease in broad-spectrum regimens (outpatient: 50% vs. 26.8%, p = 0.02; inpatient: 100% vs. 66.7%, p = 0.02), in particular macrolides, and an increase in narrow-spectrum (inpatient: 0% vs. 33.3%, p = 0.02). Post-CP children received fewer antibiotic courses (outpatient: 10 vs. 8 median DOT, p<0.0001; inpatient: 18,5 vs 10 median DOT, p = 0.004) and LOT decreased for outpatient (10 vs 8 median LOT, p<0.0001). No difference in treatment failure was reported in both outpatient and inpatient |
| Doyon et al [108] | 2009 | Quantitative evaluation of a clinical intervention aimed at changing prescriber behaviour in response to new guidelines | BA | CAN | ED + inpatient | • | Consultation by email, small group educational sessions, implementation of guideline, pre-printed prescription sheet, educational session | Guidelines compliance increased from 20.1% to 52.9%. An inappropriate choice of antibiotic agent decreased from 66.6% to 42.1 % |
| McDaniel et al [109] | 2018 | A Multisite Intervention for Pediatric Community-acquired Pneumonia in Community Settings | BA | USA | ED + inpatient | •• | Implementation of the CAP pathway occurred between January and February2016: 1) in-person presentation and distribution of electronic copies, 2) display of the printed pathways in provider work areas, 3) an in-person presentation of the pathway to community hospital pediatric hospitalists ati site-specific staff meetings, An additional educational session was conducted with both EM providers and pediatric hospitalists at all three sites at the beginning of respiratory season in October 2016. | Adherence to process measures increased postintervention for appropriate laboratory testing, narrow-spectrum antibiotic stewardship and macrolide stewardship by 10.8% (95% CI 4.7% to 16.9%), 8.3% (95% CI = 21.5% to 15.2%), and 3.1% (95% CI = –4.3% to 10.4%), respectively |
| Rutman et al [110] | 2017 | A Comprehensive Approach to Pediatric Pneumonia: Relationship Between Standardization, Antimicrobial Stewardship, Clinical Testing, and Cost | BA | USA | ED + inpatient | *•* | Clinical pathway for CAP | Increase in narrow-spectrum antibiotic (ampicillin) use from (8 to 54%). No significant changes to cost of care were noted for patients who were seen only in the ED and discharged home |
| Yeo et al [111] | 2020 | Knowledge translation in Western Australia tertiary paediatric emergency department: An audit cycle of effectiveness of guideline dissemination on bronchiolitis management | BA | AUS | ED + inpatient | •• | Update local bronchiolitis guideline, education, email | The dissemination process of the update local hospital bronchiolitis guideline did not show any statistically significant reduction of unnecessary interventions inclueded antibiotic prescription in the hospital |
| Aoybamroong et al [112] | 2019 | Impact of an Antibiotic Stewardship Program on Antibiotic Prescription for Acute Respiratory Tract Infections in Children: A Prospective Before-After Study | BA | THA | ED + Primary care | •• | Education for pediatric faculty staff, residents, and fellows via providing guidelines of antibiotic smart use in respiratory tract infections; the guidelines were sent to all relevant physicians via email or LINE instant messaging every 2 months.Poster displaying the guidelines were posted in every examination room and active monitoring was conducted by informing physicias of the antibiotic use rate every 2 months via email or line | ASP increased appropriateness of antibiotic presciption for ARTI from 77.5% to 83.4%, with the greatest impact amoneg faculty staff expecially in semiprivate clinics (75% to 83%, p<0,001) |
| Di Pietro et al [113] | 2017 | Monitoring adherence to guidelines of antibiotic use in pediatric pneumonia: the MAREA study | BA | ITA | ED + Primary care | • | 1 day-educational intervention and coincise written materials | In not hospitalized CAP, educational intervention was followed by a 25% decrease in macrolide prescription (53.13% vs. 39.71%, p = 0.12) whereas there was no difference in AP in hospitalized CAP |
| March-Lopez et al [114] | 2020 | Impact of a Multifaceted Antimicrobial Stewardship Intervention in a Primary Health Care Area: A Quasi-Experimental Study | BA | ESP | ED + Primary care | ••••• | Face-to-face sessions (education) and poster : Point od care test for GAS, Every 3 months, updated report containing qualitative and quantitavie indicators on antibiotics consumption, Set of local guidelines on antibiotic usage and preferred regimens for adult and pediatric patients, interactive workshop, worhshop material by email | Overall antibiotic consumption decreased by 19.22% in the pediatric poputlatio (10.05 to 8.12 DID). |
| Poole et al [115] | 2020 | Improving Antibiotic Prescribing for Children With Urinary Tract Infection in Emergency and Urgent Care Settings | BA | USA | ED + Primary care | •• | Clinical pathway for uncomplicated UTI. The pathway was implemented through the introduction of a decision-making algorithm and an electronic order set in June 2010 and again in December 2011. Information on the pathway was also included in a newletter distributed to providers at the start of the intervention. Reminders to utilize the pathway were proveded quarterly via electronic mail | A clinical pathway produced a significant and sustained increase in narrow-spectrum empiric antibiotic prescribing for pediatric UTI, with an increase in oral first generation cephalosporins (cephalexina) prescribing after the CP implementation (19,2% pre vs 79,6& post) and a significant decline in oral third generation cephalosporins (cefixime) prescribing (50,3% vs 4,0%) without an increase of treatment failure or adverse patient outcomes. The percentage of positive urine culture isolates resistant to antibiotic prescribed decreased from 7,7% prior to the intervention to 5,2% after the intervention, although this change was not significant. |
| Saha et al [116] | 2017 | Urine Culture Follow-up and Antimicrobial Stewardship in a Pediatric Urgent Care Network | BA | USA | ED + Primary care | *•* | Standard protocol for urine culture follow-up and discontinuiation of unnecessary antibiotics | The antibiotic discontinuation rate increased from a baseline mean of 4% to a mean of 84% |
| Shaw et al [117] | 2020 | Improving antibiotic prescribing in the emergency department for uncomplicated community-acquired pneumonia | BA | USA | ED + Primary care | *•* | New CPG was implemented and knowledge of implementation was disseminated via email | Providers in the ED are aware of the PIDS/IDSA guideline regarding the first line therapy for uncomplicated CAP, however this knowledge does not translate into clinical practice. |
| Widmer K et al [118] | 2021 | Use of procalcitonin in a febrile infant clinical pathway and impact on infants aged 29 to 60 days | BA (ITS) | USA | ED, Inpatient | •• | Febrile infant clinical pathway with procalcitonin level for risk stratification. | In the adjusted interrupted time series analysis, there was no immediate level change : antibiotics (1.17 [95% CI: 0.56-2.43]) and there was no slope change post-CP versus pre-CP |
| Abdulla et al [119] | 2023 | The effect of Telehealth Antimicrobial Stewardship Program (Tele-ASP) on antimicrobial use in a pediatric intensive care unit: Pre- and post-implementation single center study | BA | KWT | Inpatient | •• | Short educational sessions, weekly prospective audit and feedback (telehealth) | Average monthly antimicrobial use decreased from 922 (95%CI 745–1000) to 485 DOT/1000 patient-days (95%CI 246–722, P < 0.05). A decline in DOT was observed across most antibiotic classes, except for ceftriaxone. No effect on the length of PICU stay, length of hospitalization, or mortality was observed. |
| Achten et al [120] | 2018 | Sepsis calculator implementation reduces empiric antibiotics for suspected early-onset sepsis. | BA | NLD | Inpatient | •• | sepsis calculator in addition to and in accordance with existing protocols | Antibiotic therapy for suspected EOS was reduced by 44% following implementation of the calculator |
| Acuna et al [121] | 2022 | FilmArray Meningoencephalitis panel in the diagnosis of central nervous system infections: stewardship and cost analysis in a paediatric hospital in Chile | BA | CHL | Inpatient | • | FilmArray® Meningoencephalitis (FAME) panel | There was no significant difference in the use of antibiotics upon admission to the hospital, but there was a difference in the use of antivirals, with a greater use recorded in the post-intervention period (p < 0.001) |
| Adams et al [122] | 2019 | Does an Antimicrobial Time-Out Impact the Duration of Therapy of Antimicrobials in the PICU? | ObsP | USA | Inpatient | • | Antimicrobial time-out (after 48-72 hours of antimicrobials) | The cohort that underwent time-outs had lower days of therapy for vancomycin (81.3 vs 138.1; p = 0.037) and meropenem (34.7 vs 67.1; p = 0.045). Time-outs led to antimicrobial duration being defined 63% of the time and deescalation or discontinuation of antimicrobials 29% of the time. |
| Agwu et al [123] | 2008 | A World Wide Web-based antimicrobial stewardship program improves efficiency, communication, and user satisfaction and reduces cost in a tertiary care pediatric medical center. | BA | USA | Inpatient | • | Automated CDS tool | A $370,069 reduction in projected annual cost associated with restricted antimicrobial use and an 11.6% reduction in the number of dispensed doses |
| Akangire et al [124] | 2020 | Implementation of the Neonatal Sepsis Calculator in Early-Onset Sepsis and Maternal Chorioamnionitis. | ObsP | USA | Inpatient | ••• | standardized guidelines and staff education for using the sepsis calculator | After implementation of the sepsis calculator and completion of the PDSA cycle, sepsis calculator use was greater than 95%, antibiotic use dropped significantly to 5% (P = .00069). |
| Akter et al [125] | 2009 | Impact of a training intervention on use of antimicrobials in teaching hospitals. | BA | MYS | Inpatient | • | Training intervention on antimicrobials prescribing by physicians in paediatric wards | Appropriate antimicrobial therapy for pneumonia and diarrhea increased by 16.4% and 56.8% respectively |
| Alejandre et al [126] | 2020 | Procalcitonin-guided protocol decreased the antibiotic use in paediatric patients with severe bronchiolitis | BA | ESP | Inpatient | •• | PCT-guided protocol to stop or de-escalate the antibiotic treatment in infants with severe bronchiolitis | The rate for antibiotic use was 79.9%, and this differed before and after implementation (88.2% vs 72.1%, P = .003). The length of antibiotic treatment was also different between the two periods (8.65 ± 4.8 days vs 5.05 ± 3.18 days, P = .023). |
| Aljassim et al [127] | 2022 | Antimicrobial Stewardship in Bronchiolitis: A Retrospective Cohort Study of Three PICUs in Canada | BA | CAN | Inpatient | • | Weekly meetings: auditing and providing feedback on the appropriateness of antimicrobial use | Among patients with viral bronchiolitis, implementation of an antimicrobial stewardship program at PICU 1 was associated with increased odds of discontinuing antimicrobials (odds ratio, 25.63; 95% CI, 2.86–326.29), but not with antimicrobial duration (odds ratio, 0.56; 95% CI, 0.31–1.02) or antimicrobial prescriptions (odds ratio, 0.33; 95% CI, 0.10–1.04). |
| Arora et al [128] | 2019 | Optimizing antibiotic use for early onset sepsis: A tertiary NICU experience | BA | USA | Inpatient | •• | Use of the Neonatal Sepsis Risk Calculator and a 36-hour time-out for prescribed empiric antibiotics. | Significant post-intervention reduction in the rate of both antibiotic prescriptions (29.4% decline; 70.3% vs. 49.6%; p < 0.001) |
| Astorga et al [129] | 2018 | Antibiotic Stewardship in the Neonatal Intensive Care Unit: Effects of an Automatic 48-Hour Antibiotic Stop Order on Antibiotic Use. | BA | USA | Inpatient | • | implementation of a 48-hour automatic stop (autostop) order during NICU admissions | Total doses given per patient decreased by 35% and doses per PD decreased by 25% (p < 0.0001). The greatest effect was a 66% decrease in the use of vancomycin, an antibiotic not included in the admission order set. The cost estimates for antibiotics decreased by 30.8% to 36.7% in the postintervention period; the largest cost decrease was seen with vancomycin. |
| Bauer et al [130] | 2021 | Using electronic health record tools to decrease antibiotic exposure in infant sepsis evaluation | ObsP | USA | Inpatient | •• | Education, Creation of Illness-Specific Infant Sepsis Evaluation (H&P), a criteria-based rule to default to this H&P template, and editing influencer smartphrases. | Percentage of patients who received >30 hours of administered antibiotic doses decreased from 75.6% to 62%. Percentage of H&P notes documenting the 36-hour phrase increased from 4.9% to 75.6%. Illness-specific H&P template and influencer smartphrase usage increased to a mean of 51.5%; length of stay did not change. No readmissions for positive culture results were reported. |
| Beavers et al [131] | 2017 | Implementation and Evaluation of the Early-Onset Sepsis Risk Calculator in a High-Risk University Nursery | BA | USA | Inpatient | •• | Kaiser early-onset sepsis (EOS) risk calculator, deviate from the Centers for Disease Control and Prevention guidelines | There was a reduction in NICU admissions rates (91% vs. 37%, p < 0.001), number of blood cultures drawn (92% vs. 50%, p < 0.001) and antibiotic administration rates (94% vs. 37%, p < 0.001) for neonates delivered to mothers with chorioamnionitis. Total charges, total bed charges, and length of stay also decreased significantly. |
| Berild et al [132] | 2008 | A controlled intervention study to improve antibiotic use in a Russian paediatric hospital. | BA | RUS | Inpatient | • | Guidelines for diagnosis and treatment of infections | The percentage of patients with gastrointestinal infections who received antibiotics decreased from 94% in 2002 to 41% in 2003, but increased to 73% in 2004. In respiratory tract infection patients these percentages were 90% in 2002, 53% in 2003 and 83% in 2004 |
| Berrondo et al [133] | 2022 | Adherence to Perioperative Antibiotic Prophylaxis Recommendations and Its Impact on Postoperative Surgical Site Infections. | ObsR | USA | Inpatient | • | Perioperative Antibiotic Prophylaxis Recommendations | Adherence was achieved for weight-based dosing in 35% of surgeries, administration prior to the incision in 91%, administration within 60 minutes (two hours for vancomycin/fluoroquinolones) in 86%, correct redosing in 97%, and to all recommendations in 29%. |
| Bobillo-Perezet al [134] | 2019 | Procalcitonin to stop antibiotics after cardiovascular surgery in a pediatric intensive care unit-The PROSACAB study | BA | ESP | Inpatient | •• | PCT-guided protocol to stop or de-escalate the antibiotic treatment | A reduction of 1.1 days of antibiotic treatment (group 1, 7.7±2.2 and group 2, 6.7±2.2, with *p* = 0.005) and 2 more antibiotic free-days free in PICU in group 2 were observed (*p* = 0.001), without adverse outcomes. |
| Borzecka et al [135] | 2021 | Antibiotic usage at a clinical paediatric hospital before and after the implementation of actions related to the hospital antibiotic policy | BA | POL | Inpatient | • | Hospital antibiotic policy | Wards with pre-authorisation duty for third-line antibiotics (group 1 of wards) used less DDD/100 BD (from 28.81 to 31.12 DDD/100 BD) than wards without such a duty (from 54.72 to 76.06 DDD/100 BD). We observed a temporary decrease of 6.37% in DDD/100 BD in group 1 of wards and a stable 9% to 21% decrease in DDD/100 BD tendency in group 2 of wards (wards without pre-authorisation duty: oncology, haematology and intensive care unit) compared with average values of DDD/100 BD in the period before ASP-related actions (2013-2014). |
| Cantey et al [136] | 2016 | Reducing unnecessary antibiotic use in the neonatal intensive care unit (SCOUT): a prospective interrupted time-series study. | ObsP | USA | Inpatient | • | empirical antibiotic therapy was set to discontinue after 48 h in the electronic medical record and the duration of therapy for pneumonia and culture-negative sepsis was limited to 5 days. | Antibiotic use declined from 343.2 DOT/1000 PD to 252.2 DOT/1000 PD |
| Cantey et al [137] | 2022 | Remote Stewardship for Medically Underserved Nurseries: A Stepped-Wedge, Cluster Randomized Study. | BA (ITS) | USA | Inpatient | •• | Education, audit, and feedback; and 24/7 infectious diseases provider-to-provider phone consultation availability | Infants exposed to antibiotics declined from 6.2% pre-ASP to 4.2% post-ASP (relative risk 0.68 [95% confidence interval, 0.63% to 0.75%]). Total antibiotic use declined from 117 to 84.1 days of therapy per 1000 patient-days (-28% [95% confidence interval -22% to -34%]. |
| Caruso et al [138] | 2017 | A quality improvement initiative to optimize dosing of surgical antimicrobial prophylaxis. | BA | USA | Inpatient | • | new electronic medical record order sets, personal provider antibiotic dose badges, and utilization of pharmacists to prepare antibiotics to increase compliance with the recommended dose. | The rate of compliance of administering cefazolin at 30 mg/kg was significantly higher when given after an electronic order than when given verbally, 94% vs 76% |
| Ceradini et al [139] | 2017 | Telemedicine as an effective intervention to improve antibiotic appropriateness prescription and to reduce costs in pediatrics. | BA | ITA | Inpatient | • | remote infectious disease consultancy program via telemedicine | It was observed a not statistically significant difference in hospital infections intensive care unit rate. The rate of multi drug resistant isolation decreased from 104 to 79 per 1000 PD (-25%, p = 0.01). The overall costs of antimicrobials fell dramatically (25000 €/year vs. 15000 €/year) due to a lower utilization of complex molecules |
| Chan et al [140] | 2015 | Implications and Impact of Prior Authorization Policy on Vancomycin Use at a Tertiary Pediatric Teaching Hospital. | BA | USA | Inpatient | • | Prospective audit with intervention and real time feedback | Vancomycin use declined from 378 doses administered/1000 PD to 208 doses administered/1000 PD (45%). Following the implementation of preauthorization, vancomycin use decreased by an additional 16% |
| Chiotos et al [141] | 2022 | Improving Vancomycin Stewardship in Critically Ill Children | ObsP | USA | Inpatient | ••• | Stakeholder education, generation of a consensus-based guideline for empiric vancomycin use, implementation of this guideline through clinical decision support. | During the 3-year QI project, overall vancomycin DOT per 1000 patient days in the PICU decreased from a baseline mean of 182 DOT per 1000 patient days to 109 DOT per 1000 patient days (a 40% reduction). |
| Chiu et al [142] | 2011 | Effectiveness of a Guideline to Reduce Vancomycin Use in the Neonatal Intensive Care Unit. | BA | USA | Inpatient | • | guideline to restrict vancomycin use in the NICU | Vancomycin start rates were reduced from 6.9 to 4.5 per 1000 PD (-35%; p <0 .01) at hospital 1, and from 17 to 6.4 per 1000 PD (-62%; p < 0.0001) at hospital 2. The number of infants exposed to vancomycin decreased from 5.2 to 3.1 per 1000 PD (-40%; p < 0.008) at hospital 1, and 10.8 to 5.5 per 1000 PD (-49%; p < 0.009) at hospital 2. Causes of infection, duration of bacteremia, and incidence of complications or deaths attributable to late-onset infection did not change significantly at either institution |
| Ciofi degli Atti et al [143] | 2017 | A collaborative intervention to improve surgical antibiotic prophylaxis in children: results from a prospective multicenter study. | BA | ITA | Inpatient | ••• | local guidelines, educational meetings, development and dissemination of standardized educational material | Adherence to recommendations was significantly higher for procedures with indication to SAP (87.6%), compared to procedures with no indication (82.8%) (p < 0.01). Increase of exclusive use of penicillins/1st-or 2nd-gen. cephalosporins in the post intervention and follow up period (49.1% vs.68.3% vs. 72.5%) and a concomitant decrease of 3rd- or 4thgen. cephalosporins/carbapenems/tazobactam and piperacillin (23.1%vs. 4.4% vs.2.9%) over time. Appropriate timing and duration increased in the follow up period compared to the preintervention (48.6% vs. 70.0% - 39.6% vs. 53.3%) |
| Coggins et al [144] | 2013 | Use of a computerized C-reactive protein (CRP) based sepsis evaluation in very low birth weight (VLBW) infants: A five-year experience. | BA | USA | Inpatient | •• | CRP-guided computerized decision support (CDS) algorithm | The infants in the compliance group received significantly less doses of ampicillin (5 vs. 14, p<0.001) and a smaller total dose of ampicillin (0.52 mg/g vs. 1.38 mg/g, p<0.001) compared to the 160 patients in the non-compliant group |
| Colletti et al [145] | 2019 | A multifaceted quality improvement project improves intraoperative redosing of surgical antimicrobial prophylaxis during pediatric surgery | BA | USA | Inpatient | ••• | change in cefazolin dosing: electronic countermeasures to display previous and next dose timing, an alert 5 minutes prior to next dose, and weight-based dose recommendation (badge cards, posting of guidelines, and updates to housestaff manual) | Mean monthly compliance with redosing was 4.3% (May 2014-April 2015) and 73% (November 2015-October 2017) (P < 0.001). Dose-only compliance increased from 76% to 89% (P < 0.001), and time-only compliance increased from 4.9% to 82% (P < 0.001). |
| Cotter et al [146] | 2021 | Clinical impact of a diagnostic gastrointestinal panel in children | BA | USA | Inpatient | • | Rapid multiplex polymerase chain reaction GIP | In the GIP era, there was a decreased time to treatment (11 vs 35 hours). Although there was a decrease in LOS by 2 days among those who received treatment of a bacterial and/or parasitic pathogen (5.1 vs 3.1; P < .001), this represented only 3% of tested children. |
| Cowart et al [147] | 2022 | Implementation of an Automatic 48-Hour Vancomycin Hard-Stop in a Pediatric Community Hospital | BA | USA | Inpatient | ••• | Education: in-person education at the pediatric department meeting, education flyer emailed Vancomycin hard-stop at 48 hours | The median DOT per ordered course of vancomycin was 1.58 days (IQR, 1.00–2.59) in the pre-implementation group compared with 1.55 days (IQR, 1.00–1.99) in the post-implementation group (p = 0.51). Fewer vancomycin courses continued beyond 48 hours after hard-stop implementation (23% versus 33%). Overall, the total yearly drug acquisition cost savings to the pharmacy equated to $3000. |
| Cunningham et al [148] | 2020 | Effectiveness of a clinical pathway for pediatric complex appendicitis based on antibiotic stewardship principles | BA | USA | Inpatient | • | novel CA pathway limiting postoperative antibiotics | No differences in postoperative LOS (slope − 0.008; p = 0.855), intra-abdominal abscess rate (5% vs. 8%; p = 0.135), or readmission rate (12% vs. 8%; p = 0.113) across time periods |
| da Silva et al [149] | 2021 | Quality analysis of antimicrobial restriction policy in pediatrics | ObsP | BRA | Inpatient | • | Antimicrobial policy restriction | 111 (92.5%) requisitions were approved. In five refused requisitions, a narrow-spectrum antimicrobial was prescribed after further discussion; four were refused due to lack of information, and in one case, the de-escalation of the antimicrobial was possible. No mistake regarding dosage and duration was detected. |
| Dassner et al [150] | 2017 | Evaluation of a Second-Sign Process for Antimicrobial Prior Authorization. | BA | USA | Inpatient | • | electronic medical record as a pediatric ASP strategy | Appropriateness of second-sign restricted antibiotic use significantly increased (84.5% to 92.9%) |
| del Rosal T [54] | 2024 | Multiplex PCR and Antibiotic Use in Children with Community-Acquired Pneumonia | BA | ESP | Inpatient | • | mPCR respiratory panel | There were no statistically significant differences in the total antibiotic consumption (83% of cases and 86% of controls) or antibiotics given for ≥72 h (58% vs. 66%). Antibiotics were prescribed in 41% of the cases and 72% of the controls at discharge (p = 0.001). Ampicillin was the most commonly prescribed antibiotic among the patients (44% vs. 18% for controls, p = 0.004), while azithromycin was the most commonly prescribed among the controls (19% vs. 48% for patients and controls, respectively; p = 0.001). |
| Di Pentima et al [151] | 2009 | Antimicrobial Prescription Errors in Hospitalized Children: Role of Antimicrobial Stewardship Program in Detection and Intervention. | ObsP | USA | Inpatient | • | actively monitored 13 targeted Ams (CareNet and PharmNet) | Errors rate associated with these was 0.09/1000 doses administered and 5 errors/1000 PD |
| Di Pentima et al [152] | 2010 | Impact of Antimicrobial Stewardship Program on Vancomycin Use in a Pediatric Teaching Hospital. | ObsP | USA | Inpatient | • | Discussion with a pediatric infectious disease physician and real-time feedback provided to clinicians | Density of vancomycin use declined overtime from 378 doses administered/1000 PD to 255 doses administered/1000 PD. The rate of vancomycin prescription errors decreased |
| Di Pentima et al [153] | 2011 | Benefits of a Pediatric Antimicrobial Stewardship Program at a Children’s Hospital. Pediatrics | BA | USA | Inpatient | • | Active surveillance of antimicrobial use with intervention and real-time feedback to providers and reinforcement of prior authorization for selected antimicrobials | Total antimicrobial use decreased to 1904 doses administered/1000 PD per year. Targeted- antimicrobial use declined from 1250 to 988 doses administered/1000 PD per year. Nontargeted-antimicrobial use declined from 1839 to 916 doses administered/1000 PD per year. Rates of antimicrobial resistance to broad-spectrum antimicrobials among the most common Gram-negative bacilli remained low and stable over time |
| Dimopoulou et al [154] | 2016 | Perioperative antimicrobial prophylaxis in pediatric patients in Greece: Compliance with guidelines and impact of an educational intervention | BA | GRC | Inpatient | •• | educational intervention to improve adherence to PAP guidelines | The percentage of patients receiving appropriate perioperative antimicrobial prophylaxis improved from 6.2% to 77.1% |
| Dommett et al [155] | 2009 | Successful introduction and audit of a step-down oral antibiotic strategy for low risk paediatric febrile neutropaenia in a UK, multicentre, shared care setting. | ObsP | GBR | Inpatient | •• | guidelines for the management of low risk of febrile neutropaenia | The intervention was associated with low hospital readmission rate (5.6%), no intensive care admissions and no deaths in low risk episodes |
| Dona et al [156] | 2019 | Effects of an antimicrobial stewardship intervention on perioperative antibiotic prophylaxis in pediatrics | BA | ITA | Inpatient | • | Clinical Pathway in perioperative antibiotic prophylaxis (PAP). | After CP implementation, there was an increase in appropriate PAP administration, as well as in the selection of the appropriate antibiotic for prophylaxis, both for monotherapy (p = 0.02) and combination therapy (p = 0.004). The overall correctness of the PAP increased significantly after the CP implementation. Cefazolin was the most used antibiotic, with a significant increase in the post-intervention period (p < 0.001) and with a reduction in the use of other broad-spectrum antibiotics. |
| Downes et al [157] | 2019 | Implementation of a Pragmatic Biomarker-Driven Algorithm to Guide Antibiotic Use in the Pediatric Intensive Care Unit: The Optimizing Antibiotic Strategies in Sepsis (OASIS) II Study | ObsP | USA | Inpatient | •• | 8 serum biomarkers daily over 72 hours, cut points of biomarker combinations | Patients without bacterial infection received a mean of 3.8 excess days of therapy. |
| Doyon et al [158] | 2009 | Quantitative evaluation of a clinical intervention aimed at changing prescriber behaviour in response to new guidelines. | BA | CAN | Inpatient | • | new community-acquired pneumonia management guidelines | Guidelines compliance increased from 20.1% to 52.9%. An inappropriate choice of antibiotic agent decreased from 66.6% to 42.1 % |
| Drwiega et al [159] | 2019 | Impact of Rapid mecA Polymerase Chain Reaction Rapid Diagnostic Testing for Staphylococcus aureus in a Pediatric Setting | BA | USA | Inpatient | • | implementation of rapid mecA detection via use of GeneXpert MRSA/SA for blood culture positive for SA | The median (interquartile range) time (hours) to optimal therapy from culture collection was decreased from 61.5 (47.8–68.1) preimplementation to 42.5 (21.9–56.6; P = 0.003) postimplementation. |
| Dukhovny et al [160] | 2019 | A collaborative multicenter qi initiative to improve antibiotic stewardship in newborns | ObsP | USA | Inpatient | •• | interactive Web sessions, a series of 4 point-prevalence audits, and expert coaching designed to help teams test and implement the CDC core elements of antibiotic stewardship | The median AU rate decreased from 16.7% to 12.1% (P for trend < .0013), a 34% relative risk reduction. |
| Esposito et al [161] | 2011 | Procalcitonin measurements for guiding antibiotic treatment in pediatric pneumonia | RT | ITA | Inpatient | •• | Algorithm based on a PCT cut-off value | The PCT group received significantly fewer AP (85.8% vs 100%; p < 0.05), were exposed to antibiotics for a shorter time (5.37 vs 10.96 days; p < 0.05), and experienced fewer antibiotic-related adverse events (3.9% vs 25.2%; p < 0.05), regardless of CAP severity. There was no significant difference in recurrence of respiratory symptoms and new AP in the month following enrollment |
| Fernandez-Polo et al [162] | 2023 | Impact of an outpatient parenteral antimicrobial treatment (OPAT) as part of a paediatric-specific PROA program. | ObsR | ESP | Inpatient | • | OPAT | Favorable clinical outcome occurred in 74.5% of the episodes. The main cause of premature interruption was unfavorable clinical outcome of the infection (37.1%). A total of 2.62 beds/day were saved, resulting in an economic benefit of 1,069,963 €. |
| Frost et al [163] | 2023 | Sustainability of Interventions to Increase Guideline-Concordant Durations of Antibiotic Therapy for Children with Acute Otitis Media | BA | USA | Inpatient | •• | Change in the electronic health record, education, feedback comparing prescribing with peers | Both EHR-only and bundled interventions substantially increased prescribing of guideline-concordant antibiotic durations for AOM, and the change in prescribing was sustained 18 months after discontinuation of clinician education and feedback reports to clinicians. The bundled intervention remained 26% (absolute percentage) more effective than the EHR-only intervention. Over a 2-year evaluation period, treatment failure and recurrence rates were low and did not increase with either intervention. |
| Gareau-Terrell et al [164] | 2020 | Can Procalcitonin Improve Antibiotic Stewardship for Late-Onset Sepsis Evaluations in Neonates? | BA | USA | Inpatient | •• | New guidelines with recommended PCT cutoff levels | Proportion of infants receiving appropriate antibiotics pre-/postinitiation of PCT guidelines did not significantly differ but clinical significance with an improvement in the proportion of appropriate antibiotic administration. |
| Gill et al [165] | 2009 | Impact of Enhanced Infection Control at 2 Neonatal Intensive Care Units in The Philippines. | BA | PHL | Inpatient | • | a simplified package of infection-control measures | Staff hand hygiene compliance improved and overall mortality declined. Colonization with resistant pathogens and sepsis rates did not change significantly at either NICU |
| Gillon et al [166] | 2017 | Vancomycin Use: Room for Improvement Among Hospitalized Children. | BA | USA | Inpatient | • | prospective audit with real-time feedback on vancomycin use | Monthly vancomycin use decreased from 114 DOT/1000 PD to 89 DOT/1000 PD |
| Goel et al [167] | 2022 | Implementation of an adapted Sepsis Risk Calculator algorithm to reduce antibiotic usage in the management of early onset neonatal sepsis: A multicentre initiative in Wales, UK | BA | GBR | Inpatient | ••• | Consensus guideline with SRC algorithm + staff training and parent education | 4304 (14.3%) of the 30 105 live-born infants received antibiotics in the baseline period compared with 1917 (7.7%) of 24 749 infants in the intervention period (45.5% mean reduction). All 19 infants with culture-positive sepsis in the postimplementation phase were identified and treated appropriately. There were no increases in sepsis-related neonatal unit admissions, disease morbidity and late readmissions. |
| Goff et al [168] | 2022 | The Impact of a Multifaceted Tertiary Pediatric Hospital's Antimicrobial Stewardship Service | BA | AUS | Inpatient | •••• | Antimicrobial guidelines and monographs with regular updates; restriction of selected antimicrobials; regular AMS ward rounds and “handshake” stewardship activities | The appropriateness of individual antimicrobial orders improved across the study periods from 6111/7040 (79.4%) in the first 2 years following implementation of the AMS program to 17,819/19,229 (92.3%) in the latter period. Guideline compliance increased from 5426/7700 (70.5%) to 17,822/19,316 (92.3%). A reduction in overall antimicrobial expenditure (34% reduction, equivalent to $12.52 per bed day) |
| Goldman et al [169] | 2015 | Clinical Diagnoses and Antimicrobials Predictive of Pediatric Antimicrobial Stewardship Recommendations: A Program Evaluation. | ObsR | USA | Inpatient | • | Audit and feedback: stop therapy, modify therapy, optimize therapy, or consult infectious diseases. | 3rd-gen. cephalosporins, (0.20) were the antimicrobials with the highest predictive probability of an ASP recommendation whereas linezolid (0.05) had the lowest probability |
| Goldman et al [170] | 2019 | Clinical impact of an antimicrobial stewardship program on high-risk pediatric patients | ObsR | USA | Inpatient | • | Stopping antibiotics; modifying antibiotic type, dose, or duration; or obtaining an infectious diseases consultation | The ASP made 2,088 recommendations, and 50% of these recommendations were to stop antibiotics. Recommendation agreement occurred in 70% of these cases. Agreement with an ASP recommendation was not associated with higher odds of mortality or hospital readmission. |
| Gong et al [171] | 2016 | Effect of Financially Punished Audit and Feedback in a Pediatric Setting in China, within an Antimicrobial Stewardship Program, and as Part of an International Accreditation Process. | BA | CHN | Inpatient | •• | Prospective Audit and Feedback, Financial Penalties “Dear doctor” letters were sent from the ASPs team to physicians. | The proportion of both AP and expenditure on antibiotics dropped immediately |
| Graus et al [172] | 2022 | Managing antibiotics wisely in a neonatal intensive care unit in a low resource setting | BA | PER | Inpatient | ••• | Multidisciplinary team, education sessions (weekly), culture change (Blood culture collection standardization and reporting), Discontinuation of antibiotics at 48 h, regular reporting of antibiotic use, EBM, journal club meetings, Introduction of the observational method for diagnosis of EOS | Antibiotic usage rate declined from 291/1000 patient-days to 82/1000 patient-days during the last months of 2020, representing a total decrease of 65.1%. |
| Grewer-Katona et al [173] | 2021 | Opportunities for Antibiotic Stewardship Interventions in a Pediatric Hospital | BA | DEU | Inpatient | •• | Weekly teaching sessions, antibiotic pocket cards, and rounds with pediatric infectious disease staff | After the AMS intervention, the use of second-generation cephalosporins decreased, while penicillin with BLI increased. Survey of antibiotic prescriptions over the 4 months study period in the non-university hospital showed a high administration rate of second-generation cephalosporins and extended-spectrum penicillins in the non-ICU wards (48.53 and 38.93 days of therapy [DoT]/1,000 PD, respectively) and a high rate of third-generation cephalosporins in the ICU ward (110.33 DoT/1,000PD). Adherence to national guidelines was highest in the NICU and pediatric ICU wards. |
| Guitart et al [174] | 2021 | Impact of a modification of the clinical practice guide of the American Academy of Pediatrics in the management of severe acute bronchiolitis in a pediatric intensive care unit | BA | ESP | Inpatient | • | Modification of the American Academy of Pediatrics guidelines for the management of bronchiolitis in hospital. | The antibiotherapy rate decreased significantly (P=.003). |
| Gustavsson et al [175] | 2020 | Reduced antibiotic use in extremely preterm infants with an antimicrobial stewardship intervention | BA | SWE | Inpatient | •• | Updated local guidelines with a focus on shortened and standardised treatment duration plus increased access to infectious disease consultant advice | Overall antibiotic use (treatment and prophylaxis) was 534 versus 466 days per 1000 patient-days during the baseline and intervention periods, respectively. Antibiotic treatment days decreased from 287 to 197 days per 1000 patient-days. The proportion of meropenem-based regimens was 69% versus 44%, respectively. |
| Hamdy et al [176] | 2020 | Reducing vancomycin use in a level IV NICU | ObsP | USA | Inpatient | •••• | physician education; pharmacy-initiated 48-hour antibiotic time-outs on rounds; development of clinical pathways to standardize empirical antibiotic choices, daily prospective audit with feedback | vancomycin use declined from 112 to 38 days of therapy per 1000 patient-days. After education, pharmacy-initiated 48-hour time-outs, and development of clinical pathways, vancomycin use declined by 29%, and by an additional 52% after implementation of prospective audit with feedback. Vancomycin-associated acute kidney injury also declined from 1.4 to 0.1 events per 1000 patientdays. |
| Hersh et al [177] | 2015 | Antimicrobial Stewardship Programs in Freestanding Children’s Hospitals. | ObsR | USA | Inpatient | • | a comprehensive program that functions continuously to monitor antimicrobial use and that dedicates full-time equivalents to support a clinical pharmacist and/or pediatric infectious diseases specialist | 8 of 9 ASP hospitals revealed declines in antibiotic use, with an average monthly decline of 5.7% DOT/1000 PD. For the select subset of antibiotics, the average monthly decline was 8.2% |
| Holzmann-Pazgal et al [178] | 2015 | Decreasing vancomycin utilization in a neonatal intensive care unit. | BA | USA | Inpatient | •• | education versus audit and feedback in decreasing vancomycin utilization | Vancomycin utilization and administration duration >3 days significantly decreased but it was not affected by addition of audit and feedback |
| Horikoshi et al [179] | 2016 | Impact of computerized pre-authorization of broad spectrum antibiotics in *Pseudomonas aeruginosa* at a children’s hospital in Japan | ObsR | JPN | Inpatient | • | computerized pre-authorization for broad spectrum antibiotics for Pseudomonas aeruginosa | Administration of carbapenems, piperacillin/tazobactam, and ceftazidime decreased significantly. Antibiotic costs were reduced by 26000$ annually. None of the antipseudomonal agents showed decreased sensitivity |
| Horikoshi et al [180] | 2017 | Sustained pediatric antimicrobial stewardship program with consultation to infectious diseases reduced carbapenem resistance and infection-related mortality. | BA | JPN | Inpatient | •• | computerized preauthorization and a prospective audit for carbapenem were implemented as core elements of the ASP. An electronic chart-based drug ordering system was designed to block orders for carbapenem automatically | A positive correlation was observed between the carbapenem resistance rate in *P. aeruginosa* and DOT (0.76, p = 0.04). The carbapenem resistance rate in P. aeruginosa (p < 0.01) and DOT (p < 0.01) decreased significantly |
| Horikoshi et al [181] | 2018 | The North Wind and the Sun: Pediatric Antimicrobial Stewardship Program Combining Restrictive and Persuasive Approaches in Hematology-Oncology Ward and Hematopoietic Stem Cell Transplant Unit | ObsR | JPN | Inpatient | •• | preauthorization of carbapenem, prospective audit with feedback, a weekly luncheon meeting among physicians, consensus on febrile neutropenia management, and implementation of viral molecular diagnostics | DOTs of cefepime, piperacillin/tazobactam, meropenem and vancomycin decreased by 20%, 45%, 57% and 38% respectively (p<0.05) |
| Huebner et al [182] | 2013 | Pilot Project of a Pediatric Antibiotic Stewardship Initiative at the Hauner Children’s Hospital | BA | DEU | Inpatient | •• | prospective-audit with feedback and formulary restriction, pre-authorization | The cost of antibiotics decreased by 62% (from 76835€ to 29315€ ). The use of teicoplanin decreased by 97%, while vancomycin consumption increased by only about 34%. There was a decline in prescriptions of carbapenems, 3rd-gen. cephalosporins and glycopeptide antibiotics (carbapenems by 19%, linezolid by 63% and 3rd-gen cephalosporines by 53%) accompanied by an increase in consumption in aminopenicillins |
| Huetz et al [183] | 2020 | Potential Impact of Umbilical-Cord-Blood Procalcitonin-Based Algorithm on Antibiotics Exposure in Neonates With Suspected Early-Onset Sepsis | ObsP | FRA | Inpatient | •• | Algorithm incorporating umbilical-cord-blood PCT level | With the PCT-based algorithm, the potential decrease in prescription rate would be 1.8% (95% CI, 1.3-2.3), corresponding to a 39% (95% CI, 37.3-40.7) relative reduction in antibiotics exposure (p < 0.05). |
| Hum et al [184] | 2014 | Developing Clinical Decision Support within a Commercial Electronic Health Record System to Improve Antimicrobial Prescribing in the Neonatal ICU. | ObsP | USA | Inpatient | • | CDS tool to improve antibiotic prescribing in NICUs | Most (63%) survey respondents were aware of the CDS tool, but fewer (37%) used it during their most recent NICU rotation |
| Hurst et al [185] | 2016 | Handshake Stewardship: A Highly Effective Rounding-based Antimicrobial Optimization Service | BA | USA | Inpatient | •• | Handshake stewardship : restriction and preauthorization, review of all prescribed antimicrobials, rounding-based, in-person approach to feedback by a pharmacist-physician team. | Overall antimicrobial use decreased by 10.9% during the 4 years of the analysis. Vancomycin use decreased by 25.7%, meropenem by 22.2% without a compensatory increase of other antipseudomonal agents |
| Kalil et al. [186] | 2019 | Pediatric Inpatient Antimicrobial Stewardship Program Safely Reduces Antibiotic Use in Patients with Bronchiolitis Caused by Respiratory Syncytial Virus: A Retrospective Chart Review | BA | CAN | Inpatient | • | audit and feedback antimicrobial stewardship program | Compared with the 2011–2012 cohort of bronchiolitis, the 2015–2016 cohort showed a decrease of 46% in mean days of therapy per 1,000 patientdays in the >28 days old age group of patients. There was also a 15.1% absolute reduction in the proportion of patients who received any antimicrobials in the hospital between the 2 cohorts (neonates included). The proportion of patients receiving antimicrobial prescriptions at discharge also decreased from 33.5% to 19%. The use of second-generation cephalosporins was eliminated in the 2016 cohort. There was a significant decrease in length of stay between the 2011–2012 and 2015–2016 cohorts, and no readmissions were documented. |
| Karaali et al [187] | 2019 | A new antibiotic stewardship program approach is effective on inappropriate surgical prophylaxis and discharge prescription. | BA | TUR | Inpatient | •••• | local guidelines updated, periodictraining sessions , feedback to the infection control committee, clinical pathway | ASP not change first dose timing rates, affect the rates of prophylactic antibiotic indication, discontinuation of SP within 24 hours and antibiotic prescription at discharge, with statistical significance (p < 0.05). In addition, ASP continued to increase its effectiveness throughout the 3rd year. |
| Karandikar et al [188] | 2020 | Limiting Vancomycin Exposure in Pediatric Oncology Patients With Febrile Neutropenia May Be Associated With Decreased Vancomycin-Resistant Enterococcus Incidence. | BA | USA | Inpatient | • | Change in the empiric FN guideline limiting vancomycin exposure | Empiric vancomycin DOT/1000 FN days decreased from 315 pre-intervention to 164 post-intervention (P < .01) in high-risk episodes and from 199 to 115 in standard risk episodes (P < .01). |
| Kashtan et al [189] | 2020 | Implementation of a Plan-Do-Study-Act framework to reduce unindicated surgical antimicrobial prophylaxis | ObsP | USA | Inpatient | ••• | Faculty meetings to review guidelines and establish consensus, publicizing guidelines with regular email reminders, and conducting ongoing compliance audits, education-based emails targeting residents with mandatory feedback loop closure | Preintervention, 40.4% (107/265) of patients received unindicated SAP. Postintervention, the rate of unindicated SAP decreased to 15.4% (6/39) after the first month and 6.2% (20/323) after 10 months, reflecting an 85% reduction across periods (p < 0.01). There was no difference in the rate of surgical site infections between the pre and postintervention cohorts (0.36% vs. 0.67%, p = 1.00). |
| Katz et al [190] | 2020 | Use of a Procalcitonin-guided Antibiotic Treatment Algorithm in the Pediatric Intensive Care Unit | RCT | USA | Inpatient | ••• | targeted education to PICU providers, procalcitonin-guided antibiotic treatment algorithm | Antibiotic DOT were not significantly different between the PCT arm (6.6, IQR: 3.1-10.9) and the usual care arm (7.6, IQR: 3-11.8; P = 0.37). More AMS recommendations were made in the procalcitonin vs. control arm (54 vs. 37; P = 0.03). Adherence with algorithm-based antibiotic recommendations was high in the procalcitonin arm (70%). |
| Ketha et al [191] | 2021 | Eliminating Use of Home Oral Antibiotics in Pediatric Complicated Appendicitis. | BA | USA | Inpatient | • | Institutional review board approval : In the pre-protocol group, a white blood cell count was checked at discharge and patients with leukocytosis were prescribed oral antibiotics to complete a total of 7 days. In the post-protocol group, no white blood cell count was checked and patients were discharged home without antibiotics. | The pre-protocol group included 71 children, and post-protocol included 58 children. There were no differences between mean postoperative days to discharge (2.57 versus 3, P = 0.0896), postoperative abscess rate (12.7% versus 12.1%, P = 1.0000), or readmission rate (12.7% versus 17.2%, P = 0.6184). None of the patients in the post-protocol group were discharged home with oral antibiotics compared with 22.5% in the pre-protocol group (P < 0.001). |
| Khorshidi-Malahmadi et al [192] | 2021 | Second-year Outcomes of Implementing Antimicrobial Stewardship Program in a Tertiary Pediatric Hospital | BA | IRN | Inpatient | •• | Prospective audit and feedback, advice from an infectious disease specialist within 72 hours of the antimicrobial’s administration. Expert assistance (infectious disease specialist and pharmacists). Small-group education : disseminating educational materials, holding meetings, and sending verbal and written reminders | Utilization of total antimicrobials decreased meaningfully by 12.41% (from 62.11 DDDs/100 PD in February 2017-January 2018 to 54.40 DDDs/100 PD in February 2019-January 2020; P = 0.024). The results showed that the reduction in non-restricted antimicrobials was less (5.43%) than restricted antimicrobials (27.6%). |
| Kinoshita et al. [35] | 2020 | The effect of preauthorization and prospective audit and feedback system on oral antimicrobial prescription for outpatients at a children's hospital in Japan | BA | JPN | Inpatient | •• | ID consultation system, education to medical staff, development of institutional guidelines of antimicrobial prescription, continuous monitoring and feedback of antimicrobials consumption, and preauthorization for broadspectrum intravenous antimicrobial | Antimicrobial consumption and cost of targeted oral antimicrobials decreased from 11.1 DOT per 1000 outpatient visits and 860,040 yen ($ 7167: 1 $ ¼ 120 yen) to 1.9 DOT per 1000 outpatient visits and 142,200 yen ($ 1185) annually, respectively (p < 0.001). Interrupted time-series analysis showed that prescriptions for targeted antimicrobials decreased rapidly after initiation of preauthorization (p < 0.001). Prescriptions for non-targeted oral antimicrobial increased temporarily (p < 0.001), but a decreasing trend was found after the initiation (p < 0.001). The appropriate prescription rate of post-intervention period increased to 58.5%. |
| Kit-Anan et al [193] | 2022 | Handshake stewardship reduces carbapenem prescription in a pediatric critical care setting | BA | THA | Inpatient | • | Handshake stewardship was performed by direct feedback to ICU physicians regarding the appropriateness of carbapenem prescriptions within 24 h. | Carbapenem consumption decreased significantly from 667 to 369 DOT/1,000 patient-ICU days, with a median difference of 292 DOT/1,000 patient-ICU days (P < 0.001; 95% confidence interval: 175-408) after HS implementation. The acceptability of the HS was 95.4%. The LOCS, 30-day infection-related mortality, and CRE rate were not significantly different between pre-and post-implementation periods. |
| Kitano et al [194] | 2019 | A simple and feasible antimicrobial stewardship program in a neonatal intensive care unit of a Japanese community hospital | BA | JPN | Inpatient | •• | Protocol : start and stop of criteria antimicrobial treatment, weekend report of blood culture result, stopping ordering antimicrobials | DOT was 175.1 and 41.6/1000 patient-days, respectively (p < 0.001) with 76.2% reduction. The percentage of neonates who had any antimicrobials and the percentage of prolonged antimicrobial treatments among neonates who had any antimicrobials decreased significantly. The methicillin-resistant rate of S.aureus rates were significantly reduced in post-ASP period (p = 0.002). |
| Kopsidas et al [195] | 2021 | Reducing duration of antibiotic use for presumed neonatal early-onset sepsis in greek nicus. A "low-hanging fruit" approach | BA (ITS) | GRC | Inpatient | • | Discontinuation of antibiotics | Declining trend in DOT/1000 patient-days relative to the pre-intervention trend (p = 0.002); a monthly decrease rate of 28.96 DOT/1000 patient-days (p = 0.001, 95%CI [-45.33, -12.60]). |
| Kreitmeyr et al [196] | 2017 | Pediatric antibiotic stewardship: successful interventions to reduce broad-spectrum antibiotic use on general pediatric wards. | BA | DEU | Inpatient | •• | Infectious diseases ward rounds (prospective-audit-with-feedback), ID consultation service, Internal guidelines on empiric antibiotic therapy. | Overall DOT and LOT decreased by 10.5 and 7.7%, respectively. Use of cephalosporins and fluoroquinolones decreased by 35.5 and 59.9%, whereas the use of penicillins increased by 15.0%. An increase in dosage accuracy was noted (78.8 vs. 97.6%) and guideline adherence for CAP improved from 39.5 to 93.5% |
| Kreitmeyr et al [197] | 2021 | Pediatric Antibiotic Stewardship: Optimization of Vancomycin Therapy Based on Individual Pharmacokinetics. | ObsP | DEU | Inpatient | •• | Internal guidelines on correct vancomycin dosing, TDM timing, installed a pharmacokinetic (PK) consultation service to adapt vancomycin dosing to individually calculated PK parameters | Percentage of patients with sustained therapeutic vancomycin trough levels increased from 17.8% to 94.7% (P < 0.001) and percentage of treatment days with therapeutic vancomycin trough levels increased from 18.4% (117/637) to 665% (155/233, P < 0.001). Readmission rate decreased from 24.4% to 5.3% (P = 0.07). |
| Labenne et al [198] | 2007 | A Population-Based Observational Study of Restrictive Guidelines for Antibiotic Therapy in Early-Onset Neonatal Infections. | ObsP | FRA | Inpatient | • | restrictive guidelines for the antibiotic therapy in EONI | The EONI cure rate was 96.8% without infectious relapse. |
| Laccetta et al [199] | 2021 | Early-onset sepsis risk calculator: a review of its effectiveness and comparative study with our evidence-based local guidelines | ObsP | ITA | Inpatient | • | Calculator’s recommendations in neonates born at ≥34 weeks’ GA. | 32/265 (12.1%) neonates ≥34 weeks' GA received antibiotics within the first 12 h of life. According to EOS calculator 55/265 (20.7%) patients would have received antibiotics with EOS incidence 2/1000 live births (p < 0.0001). |
| Lamba et al [200] | 2020 | Standardizing the approach to late onset sepsis in neonates through antimicrobial stewardship: a quality improvement initiative | ObsP | USA | Inpatient | • | Documented reason for sepsis evaluation, appropriate initial evaluation considered, appropriate antibiotic selection and appropriate antibiotic de-escalation. | There was improvement in appropriate initial antibiotic selection (70% vs. 94%); appropriate consideration of initial evaluation (63% vs. 94%, respectively); appropriate de-escalation of antibiotics (86% vs. 100%, respectively). The overall antibiotic utilization rate and length of treatment did not change significantly. |
| Lanata et al [201] | 2021 | Empiric Vancomycin Reduction in a Pediatric Intensive Care Unit. | BA | USA | Inpatient | ••• | Consensus indications for empirical vancomycin use for PICU patients, visual reminder for clinicians, paper placards, educating medical residents, modified a preexisting PICU empirical antibiotic order set in the EMR with passive decision support listing the indications for empirical vancomycin | Empirical vancomycin decreased from a baseline of 73% to 45%, a 38% relative reduction. No patient not prescribed empirical vancomycin later required the addition of vancomycin or other MRSA-targeted antibiotics. |
| Lee et al [202] | 2007 | Control of extended-spectrum β-lactamase-producing *Escherichia coli* and *Klebsiella pneumoniae* in a children’s hospital by changing antimicrobial agent usage policy. | BA | KOR | Inpatient | • | change in antibiotic policy: use of extended-spectrum cephalosporins was restricted | Piperacillin/tazobactam use increased from 2.2 to 108.0 days on antibiotics/1000 patient AD/year (p<0.001), whereas extended-spectrum cephalosporin use decreased from 175.0 to 96.9 AD (p<0.001). Among 252 strains of E. coli and K. pneumoniae, the overall prevalence of ESBL producers decreased from 39.8% to 22.8% (p< 0.018) |
| Lee et al [203] | 2016 | Reduction of Broad-Spectrum Antimicrobial Use in a Tertiary Children’s Hospital Post Antimicrobial Stewardship Program Guideline Implementation | BA | USA | Inpatient | ••• | Guideline development and education: cycles of education, retrospective review, and feedback. | Hospital-wide targeted broad- spectrum antibiotic DOT/1000 patient-days decreased from 33% to 70%. The overall antibiotic DOT decreased 41%, 21%, and 18%, and targeted broad-spectrum antibiotic DOT decreased by 99%, 75%, and 61% in the cardiac, PICU, NICU, respectively. Yearly purchases of our most common broad-spectrum antibiotics decreased 62% from $230059 to $86887 after guideline implementation. Median monthly purchases of these drugs before implementation were $19389 and $11043 after implementation (p < 0.001) |
| Lee R.A et al [204] | 2020 | Assessment of the clinical utility of plasma metagenomic next-generation sequencing in a pediatric hospital population | ObsR | USA | Inpatient | • | mNGS of cfDNA | Fourteen percent of tests impacted clinical management by changing antimicrobial therapy. |
| LeRiger et al [205] | 2020 | Improving the Compliance of Intraoperative Antibiotic Redosing: A Quality Improvement Initiative. | BA | USA | Inpatient | ••• | New organizational redosing guidelines, as well as a new antibiotic-specific reminder alert in the electronic medical record, after providing education to the anesthesiologists, surgeons, and circulating nurses | Following interventions, compliance has reached and sustained an average of 99%. (Before : 11%). SSI rate decreased from 3.19% in 2014 to 2.3% in 2018. |
| Li et al [206] | 2022 | Utility of Broad-Range PCR Sequencing for Infectious Diseases Clinical Decision Making: a Pediatric Center Experience | ObsR | USA | Inpatient | • | Broad-Range PCR Sequencing | Only 5% of BRPCR results influenced antimicrobial management |
| Liem et al [207] | 2010 | Antibiotic weight-watching: slimming down on antibiotic use in a NICU | ObsR | NLD | Inpatient | • | policy of restricted and appropriate antibiotic use | Total antimicrobial use, expressed as DOT decreased significantly, from 9.0 to 5.8 |
| Lighter-Fisher et al [208] | 2017 | Implementing an Inpatient Pediatric Prospective Audit and Feedback Antimicrobial Stewardship Program Within a Larger Medical Center | BA | USA | Inpatient | • | Audit and Feedback Antimicrobial Stewardship Program | Total antimicrobial DOT and LOT decreased significantly. The susceptibility profiles of common bacterial pathogens to antibiotics remained stable |
| Lloyd et al [209] | 2021 | Impact of a Best Practice Advisory for Pediatric Patients with Staphylococcus aureus Bacteremia | BA | USA | Inpatient | •• | EMR–based BPA for SAB, recommending ID consult and optimal antibiotic therapy | Preintervention, 48 (68.6%) patients received an ID consult compared to 27 (93.1%) postintervention, but this was not statistically significant on ITS analysis due to a preexisting trend of increasing consultation. Median hours to optimal therapy decreased from 26.1 to 5.5 (P = .03), most notably in patients with MSSA (42.2 to 10.8; P < .01). |
| Lombardi et al [210] | 2020 | Assessment of Surgical Antibiotic Prophylaxis Compliance in Pediatrics: A Pre-post Quasi-experimental Study | BA | CAN | Inpatient | ••• | Guidelines, teaching sessions, anesthesia carts in the operating room at all times | The composite partial and total compliance increased from 51.4% to 55.8% [adjusted OR 1.3; 95% confidence interval: 1.0–1.8; P = 0.06]. No significant improvement in correct timing, agent selection or duration. |
| Lu et al [211] | 2019 | Implementation of the Smart Use of Antibiotics Program to Reduce Unnecessary Antibiotic Use in a Neonatal ICU: A Prospective Interrupted Time-Series Study in a Developing Country | BA | CHN | Inpatient | •• | Smart Use of Antibiotics Program: audit, feedback, prior authorization, and point-of-prescription interventions | The total quantity of antibiotics in the intervention phase was significantly decreased from 543 days of therapy per 1,000 patient-days to 380 days of therapy/1,000 patient-days compared with that of baseline (p = 0.0001). A reduced multidrug-resistant organism rate was also observed following Smart Use of Antibiotics Program implementation (1.4% vs 1.0%; p = 0.02). |
| MacBrayne et al. [212] | 2019 | Sustainability of Handshake Stewardship: Extending a Hand Is Effective Years Later | BA | USA | Inpatient | • | Handshake stewardship | Hospital-wide mean anti-infective use significantly decreased, from 891 (95% confidence interval [CI] 859–923) in the pre-implementation phase to 655 (95% CI 637–694) DOT/1000 PD in post-implementation Year 5; in a segmented regression time series analysis, this was a rate of -2.6 DOT/1000 PD (95% CI -4.8 to -0.4). This is largely attributable to decreased antibacterial use, from 704 (95% CI 686–722) to 544 (95% CI 525 –562) DOT/1000 PD. The percentage of children ever receiving an anti-infective during admission likewise declined, from 65% to 52% (95% CI 49–54). There were no detrimental effects on severity adjusted mortality, readmissions, or lengths of stay. |
| Malcolmson et al [213] | 2016 | Impact of Matrix-Assisted Laser Desorption and Ionization Time-of-Flight and Antimicrobial Stewardship Intervention on Treatment of Bloodstream Infections in Hospitalized Children. | ObsR | USA | Inpatient | •• | Matrix-Assisted Laser Desorption and Ionization Time-of-Flight, prospective audit and feedback | Time to optimal therapy reduced (77.0 to 54.2 h). In the subgroup analysis of Gram-negative bacteremia, time to effective and optimal therapy were significantly reduced (2.0 vs 0.7 h and 146.8 vs 48.0 h, respectively) |
| McCarthy et al [214] | 2018 | Antimicrobial stewardship in the neonatal unit reduces antibiotic exposure. | ObsP | IRL | Inpatient | ••• | Electronic prescribing, re-audit and prescriber feedback | There was a significant overall reduction in the DOT/1000 PD (572 vs. 417 DOT/1000, p < 0.0001). This represents a reduction in antibiotic use by 155 DOT/1000 PD and a 27% reduction in total antibiotic use. Prolonged antibiotic treatment (>36 hours) were reduced from 82 DOT to 7.5 DOT (p = 0.0004). Treatment courses greater than five days for culture-negative sepsis were reduced from 46.5 DOT to 7 DOT (p = 0.0009) |
| McCulloh et al [215] | 2015 | Clinical Impact of an Antimicrobial Stewardship Program on Pediatric Hospitalist Practice, a 5-Year Retrospective Analysis. | ObsR | USA | Inpatient | • | ASP recommendations among hospitalist-managed children | Ceftriaxone was the most common antibiotic associated with a recommendation (154/350, 44.0%); CAP was the most common diagnosis (105/350, 30.0%). Disagreement with ASP recommendations was associated with a decreased length of stay of 15.4 (95% CI –33.2 to 1.1) hours but not 30-day readmission prevalence |
| McCulloh R.J. et al [216] | 2021 | Effect of Combined Clinical Practice Guideline and Electronic Order Set Implementation on Febrile Infant Evaluation and Management | BA | USA | Inpatient | •• | in-person education sessions + febrile infant CPGs | Overall antibiotic use and duration of antibiotic use decreased for infants 29 to 60 days (57% vs 51%, P = 0.02). |
| McMullan et al [217] | 2021 | Improving intravenous-to-oral antibiotic switch in children: a team-based audit and implementation approach. | ObsP | AUS | Inpatient | ••• | Guideline, with team-based education, audit and feedback, for timely, safe switch from intravenous-to-oral antibiotics | The percentage of children switched within 24 hours of eligibility significantly increased from 32/50 (64%) at baseline to 203/249 (82%) post-implementation (p=0.006). In addition, there was a 14-hour median reduction in hospital length of stay (p=0.008). |
| Messacar et al [218] | 2017 | Clinical impact and provider acceptability of real-time antimicrobial stewardship decision support for rapid diagnostics in children with positive blood culture results. | BA | USA | Inpatient | •• | real-time AMS decision support for children with positive blood culture results according to the FilmArray blood culture identification panel | The median time to optimal therapy decreased from 60.2 hours to 26.7 hours. Among children with blood cultures that contained true pathogens, the time to effective antimicrobial therapy decreased from 6.9 to 3.4 hours. Unnecessary antibiotic initiation for children with a culture that contained organisms considered to be contaminants decreased from 76% to 26% |
| Messacar et al [219] | 2017 | A Handshake From Antimicrobial Stewardship Opens Doors for Infectious Disease Consultations | BA | USA | Inpatient | • | unique in-person pediatric antimicrobial stewardship program | Mean monthly ID consultations per 1000 admissions increased from 31.0 to 42.0 |
| Messacar et al [220] | 2022 | Clinical and Financial Impact of a Diagnostic Stewardship Program for Children with Suspected Central Nervous System Infection | BA | USA | Inpatient | •• | Electronic medical record indication selection to guide testing : FilmArray MEP multiplex polymerase chain reaction testing of CSF | Time-to-optimal antimicrobials decreased from 28 hours among 1124 preimplementation controls to 18 hours (P < .0001) among 1127 postimplementation cases. Postimplementation, intravenous antimicrobial duration was shorter (24 vs 36 hours, P = .004). |
| Metjian et al [221] | 2008 | Evaluation of an Antimicrobial Stewardship Program at a Pediatric Teaching Hospital. | ObsP | USA | Inpatient | •• | Computerized physician order entry system and CHOP formulary monograph to contact the ASP for each targeted antimicrobial agent. Clinicians receive approval to administer a targeted antimicrobial agent | Forty-five percent of calls required an intervention by the ASP:1) Targeting the known or suspected pathogens (20%); 2) Consultation (43%); 3) Optimize antimicrobial treatment (33%); and 4) Stop antimicrobial treatment (4%) |
| Metz et al [222] | 2017 | Improvement of Guideline Adherence After the Implementation of an Antibiotic Stewardship Program in a Secondary Care Pediatric Hospital | BA | DEU | Inpatient | ••• | SOPs, audits, a weekly ward round with experts in pediatric infectious diseases and an antibiotic pocket-card for selected infectious diseases | After the ASP was implemented guideline adherence increased significantly from 33 to 63%. The consumption of cephalosporins decreased significantly (−60%), whereas aminopenicillin use increased accordingly (+120%). Neither in the pre- nor in the post-intervention group deaths occurred. This is largely attributable to decreased antibacterial use, from 704 (95% CI 686–722) to 544 (95% CI 525 –562) DOT/1000 PD. The percentage of children ever receiving an anti-infective during admission likewise declined, from 65% to 52% (95% CI 49–54). There were no detrimental effects on severity adjusted mortality, readmissions, or lengths of stay |
| Meyers et al [223] | 2020 | A quality improvement initiative to optimize antibiotic use in a level 4 NICU | ObsP | USA | Inpatient | ••• | sepsis risk calculator, adopting a 36-hour rule-out period for sepsis evaluations, a 36-hour antibiotic hard stop, and novel guideline for EOS evaluation | The AUR decreased from 27.6% at baseline to 15.5%, a 43% reduction, and has been sustained for >18 months. |
| Miller H et al [224] | 2023 | Improving Time to Stat Intravenous Antibiotic Administration: An 8-Year Quality Initiative | ObsP | USA | Inpatient | ••• | Formation of a Pediatric Sepsis Committee, routine use of automated dispensing machines for stat IV antibiotics, creation of sepsis order sets, manual and automated sepsis screening implementation, participation in national sepsis QI collaboratives, creation of difficult intravenous access guidelines, and an automated notification system for charge nurses. | Improved the stat IV antibiotics given within 1 hour of order from 33% in 2012 to 77% in 2019 and maintained this through the end of the study period in July 2020. |
| Miloslavsky et al [225] | 2017 | The Impact of Pediatric-Specific Vancomycin Dosing Guidelines: A Quality Improvement Initiative. | BA | USA | Inpatient | • | national vancomycin guidelines : standardized dosing algorithm | The time to therapeutic trough decreased from 2.78 to 1.56 days. Vancomycin-related toxicity was unchanged by the intervention (6.1% versus 4.5%) |
| Minotti C. et al [226] | 2023 | Impact of guidelines implementation on empiric antibiotic treatment for pediatric uncomplicated osteomyelitis and septic arthritis over a ten-year period: Results of the ELECTRIC study (ostEomyeLitis and sEptiC arThritis tReatment in children) | BA | ITA | Inpatient | •• | Guidelines implementation on empiric antibiotic treatment for pediatric uncomplicated osteomyelitis and septic arthritis | In OM patients, IV DOT, DOT/LOT ratio, and bsDOT were significantly lower in the guidelines group, with also the lowest proportion of patients discharged on IV treatment. |
| Molloy et al [227] | 2017 | Acceptance of Pharmacist-Driven Antimicrobial Stewardship Recommendations with Differing Levels of Physician Involvement in a Children’s Hospital. | ObsP | USA | Inpatient | • | pharmacist-physician team for daily AMS activities | Independently pharmacist driven AMS efforts were generally successful, and recommendations for antimicrobial de-escalation were better accepted after the involvement of an infectious diseases physician. |
| Mrosak J et al [228] | 2021 | The influence of integrating clinical practice guideline order bundles into a general admission order set on guideline adoption | BA | USA | Inpatient | •• | CPG + developed a candidate CDS system based on the most commonly identified barriers | CPG order bundle use increased from 27.8% to 66.6% while antibiotic ordering errors decreased from 62.9% to 18.5% with the new design. |
| Muller M.R et al [229] | 2022 | Decreased Antibiotic Exposure for Suspected Early-Onset Sepsis in the Neonatal Intensive Care Unit Through Implementation of an Antimicrobial Time-out | BA | USA | Inpatient | • | Automatic stop order and an antimicrobial time-out | These 2 simple strategies were associated with a nearly 30% reduction in antibiotic use (31 days per 1000 patient days). |
| Murni et al [230] | 2015 | Reducing hospital-acquired infections and improving the rational use of antibiotics in a developing country: an effectiveness study | BA | IDN | Inpatient | •• | hand hygiene campaign, antibiotic stewardship (using the WHO Pocket Book of Hospital Care for Children guidelines as standards of antibiotic prescribing for community-acquired infections), and other elementary infection control practices. | Major reduction in hospital acquired infections, from 22.6% to 8.6%. Inappropriate antibiotic use declined from 43% to 20.6%. Hand hygiene compliance increased from 18.9% to 62.9%. In-hospital mortality decreased from 10.4% to 8% |
| Murni et al [231] | 2020 | Multifaceted interventions for healthcare-associated infections and rational use of antibiotics in a low-to-middle-income country: Can they be sustained? | BA | IDN | Inpatient | •••• | Education, seminars, surveillance or audit, hand hygiene campaign, effective hospital therapeutics committees, guidelines for antibiotic treatment and prophylaxis and antibiotic formularies, monitoring antibiotic use and feedback to prescribers, Discussion on pediatric patients | Inappropriate antibiotic use also increased, from 20.6% (182 of 882 patients who were prescribed antibiotics) to 48.6% (545/1855) (RR 2.35 (2.04 to 2.71)). |
| Newland et al [232] | 2012 | Impact of a Prospective-Audit-With-Feedback Antimicrobial Stewardship Program at a Children’s Hospital | BA | USA | Inpatient | • | Prospective-Audit-With-Feedback | Antibiotic use decreased from 883 DOT and 567 LOT/1000 PD to 787 DOT and 523 LOT/1000 PD. Select antibiotics dropped from 353 DOT and 294 LOT/1000 PD to 311 DOT and 256 LOT/1000 PD. Antibiotic monthly usage was 6% less for both DOT and LOT per 1000 PD |
| Newman et al [233] | 2012 | Impact of a Guideline on Management of Children Hospitalized With Community-Acquired Pneumonia | BA | USA | Inpatient | •• | CPG for children hospitalized with community-acquired pneumonia | 34% increase in ampicillin use. Discharge antibiotics also changed, significant increase in amoxicillin and a significant decrease in cefdinir and amoxicillin clavulanate |
| Nguyen-Ha et al [234] | 2016 | A Quality Assessment of a Collaborative Model of a Pediatric Antimicrobial Stewardship Program | BA | USA | Inpatient | ••• | education, antimicrobial restriction, day 3 audits, and practice guidelines | Blunting of a significant downward trend for vancomycin drug starts (relative change –12%) and use (–25%). Although meropenem use was already low due to preexisting requirements for preauthorization, a decline in drug use (–31%, p = 0.021) and a nonsignificant decline in drug starts (–21%, p = 0.067) were noted |
| Nzegwu et al [235] | 2017 | Implementation of an Antimicrobial Stewardship Program in a Neonatal Intensive Care Unit | BA | USA | Inpatient | •• | guidelines for common infections, with a focus on prescriber audit and feedback | Antibiotic use decreased by 14.7 DOT/1000 PD. Ampicillin use, decreased significantly, declining by 22.5 DOT/1000 PD. Late-onset sepsis per 100 NICU days of clinical service decreased significantly, with an average reduction of 2.65 evaluations per year per provider |
| Okado et al. [236] | 2020 | Antibiotic Practice Change to Curtail Linezolid Use in Pediatric Hospitalized Patients in Hawai‘i with Uncomplicated Skin and Soft Tissue Infections | BA | USA | Inpatient | •• | guidelines and physicians education | The use of the combination of cefazolin and clindamycin as the initial treatment, compared with prior practice of monotherapy with clindamycin or cephazolin, was associated with fewer patients started on linezolid (P=.03), no increase in patients switching to linezolid (P=.97), and no significant change in LOS (P=.06). |
| Oliveira da Silva B.B. et al [237] | 2022 | The impact of monitoring software on antimicrobial management in a pediatric intensive care unit | BA | BRA | Inpatient | • | Pharmaceutical Evolution program : The system flags patients with antimicrobial prescriptions (yellow, green or red) | In the period after the implementation of software, we observed decreases in total antimicrobial consumption (P = .037). Regarding Enterobacterales, we observed a decrease in the proportion of antimicrobial resistance of first and second-generation cephalosporin classes (P = .041) and third and fourth-generation cephalosporins (P = .028). There was a decrease in the proportion of resistance of nonfermenting gram-negative bacilli to aminoglycoside scans (P = .016). We also observed evidence of a decrease in the proportion of resistance of Staphylococcus aureus agents to oxacillin (P < .001). |
| Olson et al [238] | 2020 | Oral Step-Down Therapy With Levofloxacin for Febrile Neutropenia in Children With Cancer. | BA | USA | Inpatient | • | Guideline with a goal to change practice from using IV antibiotics after hospital discharge to the use of step-down oral therapy with levofloxacin | The postimplementation period was associated with a decrease in home IV antibiotics (aRR = 0.07 [95%CI}, .03-.13]) and fewer IV antibiotic initiations within 24 hours of a new healthcare encounter up to 7 days after discharge (aRR, 0.39 [95% CI, .17-.93]) compared to the preintervention time period. |
| Otake et al [239] | 2022 | How do we reduce acyclovir overuse? Impact of FilmArray meningitis/encephalitis panel tests for pediatric patients | BA | JPN | Inpatient | •• | FilmArray meningitis/encephalitis panel tests + consultation of pediatric infectious disease specialists | The median duration of ACV decreased significantly from 6 days to 0 day (p < 0.001), and the median dose of ACV use decreased significantly from 14 vials to 0 vial (p < 0.001). No significant differences were noted in the total duration and dose of antibiotic use, LOS in PICU, and the total LOS after testing. |
| Pace D et al [240] | 2023 | Antimicrobial Stewardship in Neonates with Necrotizing Enterocolitis: A Quality Improvement Initiative | ObsP | USA | Inpatient | • | Antibiotic protocol for the management of NEC | Antibiotic exposure was reduced from a median 119.19 to 80.65 DOT per 1000 patient days (p = 0.11). Piperacillin-tazobactam exposure decreased after protocol implementation (median 68.78 vs. 7.97 DOT per 1000 patient days, p = 0.002). There were no significant differences in morbidity or mortality outcomes. |
| Pantoja, Alfonso et al [241] | 2023 | New strategies to Reduce Unnecessary Antibiotic Use in the NICU: A Quality Improvement Initiative. | ObsP | USA | Inpatient | • | Discontinuing antibiotics within 24 hours of life, "antibiotic time-out" during rounds | For all newborns admitted to our NICU, the AUR decreased, for EOS from 137 to 32 days per 1000 patient days (77% reduction) and for LOS from 277 to 121 days per 1000 patient days (56% reduction). |
| Papastergiou P et al [242] | 2022 | Implementation of a hospital antimicrobial stewardship program to improve vancomycin use in Cyprus: Challenges and opportunity | BA | CYP | Inpatient | ••• | Implementation of a protocol for treatment and TDM : education, selective reporting of antimicrobial susceptibility, providing comments on the microbiology results and regular microbiology clinical ward rounds | By implementing our vancomycin protocol, we achieved a statistically significant improvement (P<0.01) in achieving vancomycin therapeutic levels over a 2-year period, while improving administration practices. |
| Parker et al [243] | 2017 | Anti-infective Acquisition Costs for a Stewardship Program: Getting to the Bottom Line | ObsR | USA | Inpatient | • | Handshake Stewardship: prospective audit and feedback at the 24-hour and 72-hour time-points by both an ASP physician and pharmacist | Pharmacy purchasing endorsed minimal financial benefit (decrease planning to post-ASP of $590 per 1000 PD) whereas electronic medical record and pediatric hospital information system data endorsed a decrease of $12785 and $21380 per 1000 PD, respectively |
| Pauquet E. et al. [244] | 2021 | Carbapenem stewardship program in a French university children's hospital | BA | FRA | Inpatient | ••• | Revision of antibiotic treatment protocols, a half-day educational session with feedback | Overall carbapenem consumption decreased from 0.54 prescriptions per 100 admissions to 0.32 (p = 0.06). Conformity increased during the study for indication (46-87%, p = 0.004) and for reassessment (48-78%, p = 0.04) and was significantly associated with the second study period, after adjustment for ESBL carriage. |
| Pontello, Eleonora et al [245] | 2022 | Neonatal Early Onset Sepsis: Impact of Kaiser Calculator in an Italian Tertiary Perinatal Center. | BA | ITA | Inpatient | •• | EOS calculator (In electronic medical record) | There was a halving in empirical antibiotics exposure: 3% in the baseline and 1.4% in the post-EOS-implementation period, P < 0.05. The number of antibiotic days per 100 live births decreased from 15.05 to 6.36 days (P <0.05). |
| Putnam et al [246] | 2015 | Adherence to surgical antibiotic prophylaxis remains a challenge despite multifaceted interventions. | BA | USA | Inpatient | ••• | antibiotic prophylaxis guidelines, educational materials , preincisional checklist, a CPOE module, easy-to-use antibiotic guidelines | Adherence to all guideline components remained unchanged (54 vs 55%, p = 0.38). Redosing significantly improved (7 vs 53%, p = 0.02), but correct type decreased (98 vs 70%, p <0.01). The percentage of cases in which only one antibiotic guideline component was missed remained unchanged (35 vs 34%, p = 0.46) |
| Ren Z et al [247] | 2023 | Reduction of antibiotic use and multi-drug resistance bacteria infection in neonates after improvement of antibiotics use strategy in a level 4 neonatal intensive care unit in southern China | BA | CHN | Inpatient | •• | Discontinuation of antibiotic use in ruled-out sepsis within 72 h, treatment duration for culture-negative pneumonia less than 7 days, and vancomycin or meropenem was not used unless the cultured bacteria was only susceptible to them | The total antibiotic consumption decreased from 791.1 to 466.3 days of therapy per 1000 patient days from baseline to intervention period. Antibiotics were stopped within 72 h for 47.48% patients with rule-out sepsis and within 7 days for 75.70% patients with pneumonia compared with 11.56% and 37.69% during the baseline period respectively. The prevalence of multi-drug resistance bacteria decreased from 67.20 to 48.90%. The total use rate of meropenem or vancomycin decreased from 7.6 to 1.8%. |
| Renk et al [248] | 2020 | Antibiotic stewardship in the PICU: Impact of ward rounds led by paediatric infectious diseases specialists on antibiotic consumption. | BA | DEU | Inpatient | • | Weekly PID ward round with prospective audit and feedback | An 18% reduction of DOT/1000 PD was observed in the post-implementation period (p = 0.005). LOT/1000 PD decreased by 11% (p = 0.09). Meropenem and vancomycin usage were reduced by 49% (p = 0.07) and 56% (p = 0.03), respectively |
| Ross et al [249] | 2016 | Safety of Automatic End Dates for Antimicrobial Orders to Facilitate Stewardship | BA | USA | Inpatient | • | automatic end dates for antimicrobial orders | Following implementation of automatic end dates for antimicrobial orders no differences were observed in patient level of mortality or trend in mortality (p =0.37 and p= 0.57, respectively) or level of trend in readmission (p =0.88 and p= 0.28, respectively) or length of stay (p =0.75 and p= 0.43, respectively). |
| Rungsitsathian K et al [250] | 2021 | Acceptance and outcome of interventions in a meropenem de-escalation antimicrobial stewardship program in pediatrics | ObsP | THA | Inpatient | •• | implementation of local hospital guidelines, de-escalation | The incidence rate of acquisition of CR-GNB within 30 days after treatment was 5.8% in the accepted group and 15.8% in the rejected group (P = 0.03). |
| Ruvinsky et al [251] | 2014 | Effectiveness of a program to improve antibiotic use in children hospitalized in a children’s tertiary care facility in Argentina | BA | ARG | Inpatient | ••• | discussion and monitoring workshops for antibiotic prescription and distributing treatment guidelines | There was a statistically significant decline in the post intervention period in parenteral antibiotic treatments (OR=0.50 [0.35-0.70];p<0.001), especially for inpatient with fever with no clinical focus of infection and for surgical prophylaxis cases. The program decreased the proportion of inappropriate AP from 35.6% to 21.6% |
| Salau H.D et al [252] | 2023 | Antibiotic usage in a South African paediatric medical ward following the introduction of an antibiotic prescription chart | BA | ZAF | Inpatient | • | antibiotic prescription chart | Antibiotic use decreased significantly by 7.04% following the introduction of the antibiotic prescription chart (p=0.027). |
| Same R.G et al [253] | 2021 | The Association of Antibiotic Duration with Successful Treatment of Community-Acquired Pneumonia in Children | ObsR | USA | Inpatient | • | short-course (5-7 days) vs prolonged-course (8-14 days) | Four percent of children experienced treatment failure, with no differences observed between patients who received short-course vs prolonged-course antibiotic therapy (odds ratio, 0.48; 95% confidence interval, .18-1.30). |
| Savage et al [254] | 2021 | Predictive value of direct disk diffusion testing from positive blood cultures in a children's hospital and its utility in antimicrobial stewardship | ObsR | USA | Inpatient | • | dDD from positive blood cultures | Antibiotics were narrowed in 30% of cases after a dDD result and a further 25% of cases after AST result. |
| Saw C et al [255] | 2021 | Retrospective cohort study of neonatal early onset of sepsis and the role of the EOS calculator in a level II nursery | BA | AUS | Inpatient | • | Web-based EOS-calculator | By applying the EOS-calculator, a significant reduction of IV antibiotics usage from 13.4% to 3.9% (z value 10.4, p < 0.0001) could be achieved in this cohort. |
| Schwenk et al [256] | 2021 | Use of Prospective Audit and Feedback to Reduce Antibiotic Exposure in a Pediatric Cardiac ICU. | BA | USA | Inpatient | • | prospective audit and feedback program | Mean cardiac ICU IV antibiotic use decreased 20% (701 vs 880 days of therapy per 1,000 patient days, p = 0.001) during the prospective audit and feedback period compared with the preprospective audit and feedback period |
| Scott, Philip A et al [257] | 2022 | Neonatal early-onset sepsis calculator safety in an Australian tertiary perinatal centre. | BA | AUS | Inpatient | • | Neonatal EOS calculator | Using the neonatal EOS calculator, 11 neonates (35%, 95% confidence interval 19.2-54.6%) would not have received antibiotics by 24 h of age. In comparison, only one neonate (3%, 95% confidence interval 0.1-16.7%) would not have received antibiotics by 24 h of age using the current guidelines. In terms of the current practice in the cohort of patients, two neonates (6%) did not receive antibiotics by 24 h of age. |
| Seah et al [258] | 2014 | Impact of an Antimicrobial Stewardship Program on the Use of Carbapenems in a Tertiary Women’s and Children’s Hospital, Singapore. | BA | SGP | Inpatient | • | prospective-audit-and-feedback ASP implementation on the appropriate utilization of carbapenems | Significant decrease in DDD/100 PD by 55.6% from a baseline of 0.9 to 0.4 post-ASP and a reduction in DOT/100 PD by 46.7% from a baseline of 1.5 to 0.8 post-ASP without significant changes in prescription rates. Cost increased from a pre-ASP mean of $175/100 PD to a peak of $238 and decreased significantly post-ASP to a mean of $149. The month-to-month change in cost decreased significantly post-ASP |
| Seddik et al [259] | 2020 | Reducing Piperacillin and Tazobactam Use for Pediatric Perforated Appendicitis. | BA | USA | Inpatient | ••• | education, modification of electronic antibiotic orders | Piperacillin tazobactam exposure was 31 of 40 (78%) and 20 of 109 (18%) (P < 0.001), and use ≥ half of intravenous antibiotic days was 31 of 40 (78%) and 14 of 109 (13%) (P < 0.001), in the preintervention and postintervention groups, respectively. |
| Sick et al [260] | 2013 | Sustained Savings from a Longitudinal Cost Analysis of an Internet-Based Preapproval Antimicrobial Stewardship Program | ObsR | USA | Inpatient | • | internet-based preapproval antimicrobial stewardship program | The average savings from the ASP was $103787 (95% CI, $98583–$109172) per year, or $14156 (95% CI, $13446– $14890) per 1000 PD |
| Sick-Samuels et al [261] | 2023 | A Novel Comprehensive Algorithm for Evaluation of PICU Patients With New Fever or Instability | ObsP | USA | Inpatient | ••• | Fever algorithm, education during meetings, via e-mails, and in-person walk-rounds | Overall, antibiotic DOT did not change. Antibiotic initiations declined 12% from 56.9 to 49.8 initiations/1,000 patient-days in March 2021 coinciding with implementation of the third iteration (IRR, 0.88; 95% CI, 0.79–0.97). |
| Simo et al [262] | 2020 | Effects of a Paediatric Antimicrobial Stewardship Program on Antimicrobial Use and Quality of Prescriptions in Patients with Appendix-Related Intraabdominal Infections. | BA | ESP | Inpatient | ••• | Updated antibiotic guidelines for AR-IAI, pre-set antimicrobial protocols and prescription filters in e-prescription system, daily standardised evaluations, daily face-to-face and/or electronic feedback, meetings with the surgery team | Global AU, measured both as DOT/100PD and LOT, and global LOS remained unchanged in the postintervention period. The use of piperacillin-tazobactam decreased by 96% (p = 0.044). |
| Smith et al [263] | 2012 | Effectiveness of Antimicrobial Guidelines for Community-Acquired Pneumonia in Children. | BA | USA | Inpatient | •• | guidelines and education on empirical therapy for community-acquired pneumonia. | Ampicillin use increased from 2% at baseline to 6% after antimicrobial stewardship task force formation and 44% after guideline release. Ceftriaxone use increased slightly (from 56% to 59%) after task force formation but decreased to 28% after guideline release |
| So et al [264] | 2015 | Surgical Site Infection Task Force. Increasing Compliance With an Antibiotic Prophylaxis Guideline to Prevent Pediatric Surgical Site Infection. | BA | CAN | Inpatient | •••• | evidence-based AP guideline : guideline, online formulary, only recommended antibiotics were available in operating rooms, incoming trainees received orientation, antibiotic verification with time-out, computerized alerts, e-mails when guideline | There were significant improvements in appropriate antibiotic use (51.6% vs 67.0%), complete (26.2% vs 53.2%) and partial compliance (73.3% vs 88.7%), correct dosage (77.5% vs 90.7%), timing (83.3% vs 95.8%), redosing (62.5% vs 95.8%), and duration (47.1% vs 65.3%) |
| Stocker et al [265] | 2010 | Neonatal Procalcitonin Intervention Study (NeoPInS): Effect of Procalcitonin-guided decision making on Duration of antibiotic Therapy in suspected neonatal early-onset Sepsis: A multi-centre randomized superiority and non-inferiority Intervention Study. | RCT | CHE | Inpatient | •• | ùPCT guided treatment | PCT-guided decision-making resulted in a shortening of 22.4 h of antibiotic therapy |
| Stocker et al [266] | 2012 | Antibiotic surveillance on a paediatric intensive care unit: easy attainable strategy at low costs and resources. | ObsP | GBR | Inpatient | • | mandatory checklist requiring indication and recording likelihood of infection at start of antibiotic therapy and a review of the continuing need for therapy at 48 h and 5 days, reasons for continuation and possible target pathogen | The percentage of appropriate empiric antibiotic therapy courses for culture-negative infection-like symptoms increased from 18% to 74% (p<0.0001), DOT <3 days increased from 18% to 35% (p=0.05) and correct targeting of pathogen increased from 58% to 83% ( p=0.21) |
| Stocker et al [267] | 2017 | Procalcitonin-guided decision making for duration of antibiotic therapy in neonates with suspected early-onset sepsis: a multicentre, randomised controlled trial (NeoPIns). | RT | GBR | Inpatient | •• | PCT-guided decision making | For the PCT group, the duration of antibiotic therapy was reduced (intention to treat: 55.1 vs 65.0 h; per protocol: 51.8 vs 64.0 h) |
| Taylor M et al [268] | 2021 | Intermittent Education and Audit and Feedback Reduce Inappropriate Prescribing of Oral Third-Generation Cephalosporins for Pediatric Upper Respiratory Tract Infections | ObsP | USA | Inpatient | •• | Educational session, three individual audit and feedback sessions, and one group feedback session. | Following interventions, the mean percentage inappropriate oral 3GC decreased from 72% to 45% (absolute reduction 27%, p < 0.001), which was sustained the year following the last PDSA cycle (absolute reduction 26%, p < 0.001). Total monthly oral 3CG prescribing at Clinic A decreased over time, but not in four control clinics. |
| Thampi et al [269] | 2019 | Prospective audit and feedback on antibiotic use in neonatal intensive care: A retrospective cohort study | BA | CAN | Inpatient | • | Prospective audit and feedback | Overall antibiotic use decreased to 339 days of therapy per 1000 patient-days from 395 (14%, P < 0.001), without an increase in mortality |
| Ting et al [270] | 2019 | Reduction of Inappropriate Antimicrobial Prescriptions in a Tertiary Neonatal Intensive Care Unit After Antimicrobial Stewardship Care Bundle Implementation. | BA | CAN | Inpatient | ••• | Protocol, audit and real-time feedback, instrumented blood culture system | After the introduction of ASP program, 22.2%, 7.5%, 5.4% and 0% of meropenem, cefotaxime, vancomycin and linezolid courses, respectively, were inappropriate (Before : 26.3%, 12.1%, 11.4% and 0%). The numbers of inappropriate antibiotic-days/1000 days of therapy with cefotaxime and vancomycin decreased. |
| Tolia et al [271] | 2017 | Implementation of an Automatic Stop Order and Initial Antibiotic Exposure in Very Low Birth Weight Infants. | BA | USA | Inpatient | • | Automatic Stop Order and Initial Antibiotic Exposure | The median DOT decreased from 6.5 to 4 (p < 0.001), a 38% reduction, and the DOT/1000 PD decreased from 99.5 to 71.7 (p < 0.001), a 28% reduction. The percentage of infants with antibiotic use > 48 hours was also significantly lower (63.4% vs. 41.3%, p < 0.001) |
| Turner et al [272] | 2017 | Impact of an Antimicrobial Stewardship Program on Antibiotic Use at a Nonfreestanding Children’s Hospital. | BA | USA | Inpatient | • | physician-group engagement and pharmacist prospective auditing and feedback | Antibiotic use decreased by 16.8% (95% CI 18.0% to −9.2%). Vancomycin use decreased by 38%, whereas antipseudomonal β-lactam use was unaltered. Drug-acquisition cost savings were estimated to be $67000/year over the 2-year post-intervention period |
| Uhl, Bethany et al [273] | 2021 | Increasing Adherence to Acute Otitis Media Treatment Duration Guidelines using a Quality Improvement Approach. | ObsP | USA | Inpatient | •••• | Clinician and family education, clinical decision support, discharge template that defaulted to a 7-day duration of antibiotics for patients 2 years and older diagnosed with AOM | The percentage of patients diagnosed with AOM receiving a short antibiotic course increased from a baseline of 7% to a new centerline mean of 67%, which exceeded the goal. |
| Velasco-Arnaiz et al. [274] | 2020 | Benefits of a Pediatric Antimicrobial Stewardship Program in Antimicrobial Use and Quality of Prescriptions in a Referral Children's Hospital | BA | ESP | Inpatient | • | postprescription review with feedback | Total antimicrobial use and antibacterial use significantly decreased during the intervention period (P = .002 and P = .001 respectively), and total antifungal use remained stable. A significant decline in parenteral antimicrobial use was also observed (P < .001). An increasing trend in the rate of optimal prescriptions was observed after the first point-prevalence survey (P = .0898). Nonoptimal prescriptions were more common in surgical than in medical departments, in antibacterial prescriptions with prophylactic intention, and in empirical more than in targeted treatments. No significant differences were observed in terms of mortality or readmission rates. Only minor changes in antimicrobial resistance rates were noted. |
| Verma A et al [275] | 2022 | Impact of antimicrobial stewardship and screening on multidrug resistant organisms: Epidemiology, outcome and associated risk factors in paediatric liver transplant patients | BA | GBR | Inpatient | ••• | Routine review of antimicrobials through daily remote notifications by a consultant medical microbiologist, weekly ward rounds and in multi-disciplinary team meetings, antibiotic guidelines for transplant patients | Colonisation and infection rate due to Carbapenem-resistant Enterobacteriaceae was 6% and 3%, respectively, during screening and AMS, compared to historical control of 25% and 30%, respectively, without screening and AMS. There was significant reduction in VRE and CRE infection during AMS period in comparison to historical control. |
| Villanueva P et al [276] | 2021 | Impact of an antimicrobial stewardship intervention in neonatal intensive care: Recommendations and implementation | ObsP | AUS | Inpatient | • | Weekly AMS audit-feedback joint ward round (6-month period) | The majority (45, 73%) of recommendations were accepted, resulting in significant improvement in the proportion of the 233 episodes that had completely appropriate antibiotic prescribing: 175 (75%) to 217 (93%) (relative risk 1.2, 95% confidence intervals 1.1-1.3, P < 0.001). |
| Villaverde S et al [277] | 2023 | PACTA-Ped: Antimicrobial stewardship programme in a tertiary care hospital in Spain | BA | ESP | Inpatient | • | Reviewed all antimicrobial prescriptions, daily sessions with the Microbiology Department, | We found average decreases of 27.8% in the days of treatment per 1000 inpatient days and 22.9% in the number of antimicrobial starts per 1000 admissions in P2. The use of carbapenems, cephalosporins and glycopeptides decreased in P2 compared to P1. The average annual cost of antimicrobial treatment decreased from є150 356/year during P1 to є98 478/year in P2. |
| Vyas, Dipen et al [278] | 2022 | Reduction of unnecessary antibiotic days in a level IV neonatal intensive care unit. | BA | USA | Inpatient | ••• | New consensus guidelines for antibiotic duration, prospective audit, antibiotic stop dates and justification for treatment duration. Educational sessions. | After sequential interventions, the percentage of UAD decreased from 42% to 12%, which exceeded our goal of a 20% decrease. Compliance with antibiotic stop dates increased from 32% to 76%, and no antibiotics were reinitiated within 2 weeks. |
| Walker et al [279] | 2017 | Antibiotic stewardship in the newborn surgical patient: A quality improvement project in the neonatal intensive care unit. | BA | USA | Inpatient | • | standardized antibiotic administration for surgical neonates: antibiotic protocol | Surgical site infection rates were similar pre- and post-protocol, 14% and 9% respectively. The incidence of hospital-acquired infections (13.7% vs 8.7%) and multidrug-resistant organism (4.7% vs 1.6%) was similar between the 2 periods |
| Wattier et al [280] | 2017 | Reducing Second Gram-Negative Antibiotic Therapy on Pediatric Oncology and Hematopoietic Stem Cell Transplantation Services. | BA | USA | Inpatient | • | institutional FN guideline for the pediatric oncology service | Phase 1 had mixed effects–long-term reduction in tobramycin use (97% below projected at 18 months) but rebound with increasing slope in ciprofloxacin use (+18% per month). Following phase 2, tobramycin and ciprofloxacin use on the oncology service were both 99% below projected levels at 12 months. On the HSCT service, tobramycin use was 99% below the projected level and ciprofloxacin use was 96% below the projected level at 12 months |
| Webber et al [281] | 2013 | Conversion of a single-facility pediatric antimicrobial stewardship program to multi-facility application with computerized provider order entry and clinical decision support. | ObsR | USA | Inpatient | • | Computerized Provider Order Entry and Clinical Decision Support | 437 incidents were documented, 1.1% of which were associated with ASP content or workflow |
| Willis et al [282] | 2016 | Reducing Antimicrobial Use in an Academic Pediatric Institution: Evaluation of the Effectiveness of a Prospective Audit With Real-Time Feedback | BA | USA | Inpatient | • | ASP pharmacist reviews these reports, the ASP pharmacist and physician review many cases regarding the best course of action before making recommendations. | Parenteral antimicrobial use was decreasing at our hospital by 3.7%/year, similar to the 3.4%/year found across children’s hospitals. The rate of change after implementation of the ASP at our hospital was 11.1%/year, compared to 5.6%/year for other hospitals over the same period |
| Woods-Hill et al [283] | 2022 | Association of Diagnostic Stewardship for Blood Cultures in Critically Ill Children with Culture Rates, Antibiotic Use, and Patient Outcomes: Results of the Bright STAR Collaborative | ObsP | USA | Inpatient | ••• | Unique clinical decision support tools to guide blood culture practice, educational sessions, electronic clinical pathways, audit and feedback of culture rates to PICU clinicians, and targeted emails to PICU clinicians. | Comparing the periods before and after implementation, the rate of broad-spectrum antibiotic use decreased from 506 days to 440 days per 1000 patient-days/mo, 13% relative reduction. The broad-spectrum antibiotic initiation rate decreased from 58.1 to 53.6 initiations/1000 patient-days/mo. Rates of CLABSI decreased from 1.8 to 1.1 per 1000 central venous line days/mo. |
| Wright M.R. et al [284] | 2025 | Requiring durations of therapy at the time of antibiotic order entry reduces antibiotic use | BA | USA | Inpatient | •• | Reviewed antibiotic medication orders with an indication of empiric treatment for suspected infection + requiring enter stop date | Requiring ordering clinicians to enter stop dates at the time of antibiotic order entry decreased DOT/1000 patient days for orders with empiric indication from 154 to 119 (–34.9 (–55.7 to –14)). |
| Wu et al [285] | 2017 | Comparison of Procalcitonin Guidance-Administered Antibiotics with Standard Guidelines on Antibiotic Therapy in Children with Lower Respiratory Tract Infections: A Retrospective Study in China. | BA | CHN | Inpatient | •• | algorithm based on the biomarker PCT | Antibiotic prescribing rates were significantly different in the PCT group compared to the standard group: 54.64% versus 83.91% (difference: –29.26%; 95% CI: –38.31, –20.22; p = 0.23). Mean duration of antibiotic exposure in the PCT group (3.98 ± 2.17 days) was lower than the standard groups (6.66 ± 5.59 days) (difference: –2.68%; 95% CI: –3.21 to –2.16) |
| Yonts, Alexandra et al [286] | 2023 | Multidisciplinary Initiative to Increase Guideline-concordant Antibiotic Prescription at Discharge for Hospitalized Children with Uncomplicated Community-acquired Pneumonia. | ObsP | USA | Inpatient | •• | Small bimonthly group didactic sessions, Visual job aids posted in resident work areas, A noon conference session. | After our interventions, the average monthly percentage of children discharged with guideline-concordant antibiotics increased to 87%, with the increase persisting for at least 12 months. There were no significant differences in balancing measures pre- and post-interventions. |
| Yoshida et al. [287] | 2021 | Application of multiplex polymerase chain reaction for pathogen identification and antibiotic use in children with respiratory infections in a picu | BA | JPN | Inpatient | • | multiplex polymerase chain reaction testing in children with respiratory infections in a PICU | There were no differences in antibiotic use (84% vs 75%; p = 0.14), broad-spectrum antibiotic use (33% vs 34%; p = 0.91), or the duration of antibiotic use within 14 days of admission (6.0 vs 7.0 d; p = 0.45) between the pre- and postmultiplex polymerase chain reaction periods. |
| Yoshimura J et al [288] | 2022 | Effect of Gram Stain-Guided Initial Antibiotic Therapy on Clinical Response in Patients With Ventilator-Associated Pneumonia: the GRACE-VAP Randomized Clinical Trial | RCT | JPN | Inpatient | • | Gram stain–guided antibiotic therapy or guideline-based antibiotic therapy | Reduced use of antipseudomonal agents (30.1%; 95% CI, 21.5%-39.9%; P < .001) and anti-MRSA agents (38.8%; 95% CI, 29.4%-48.9%; P < .001) was observed in the Gram stain–guided group vs guideline-based group. |
| Yu et al [289] | 2016 | Integrating a Rapid Diagnostic Test and Antimicrobial Stewardship. | BA | USA | Inpatient | • | MALDI-TOF MS for routine species identification of Gram-negative bacteria directly from positive blood cultures | Targeted antibiotic use for infections caused by methicillin-susceptible S. aureus improved (44%–80%), including when final culture results were not available |
| Zihlmann-Ji J et al [290] | 2021 | Reduction of Duration of Antibiotic Therapy for Suspected Early-Onset Sepsis in Late-Preterm and Term Newborns After Implementation of a Procalcitonin-Guided Algorithm: A Population-Based Study in Central Switzerland | BA | CHE | Inpatient | • | PCT-guided therapy using the NeoPInS algorithm | Statistically significant reduction of antibiotic therapy from 4 (median, IQR 3-6) to 3 calendar days (median, IQR 2-4) from 2014 to 2018 |
| Gunnlaugsdottir et al. [291] | 2021 | Encouraging rational antibiotic prescribing behaviour in primary care – prescribing practice among children aged 0–4 years 2016–2018: an observational study | BA | ICE | Outpatient | •• | quality project on prudent prescribing of antibiotics in primary healthcare | A reduction of 9% in the total number of prescriptions between 2017–2018 was observed. During this period, the prescribing of co-amoxiclav and macrolides decreased by 52.3% and 40.7%, respectively. These changes were significant in all cases, p < 0.0001. |
| Machnes et al. [292] | 2023 | Antibiotic Stewardship for Community-Acquired Pediatric Pharyngitis: A Pre-Post Intervention Study | BA | ISR | Outpatient | • | one-day seminar for primary care pediatricians | There was no change in the types of antibiotics prescribed before and after the intervention (P = 0.152). |
| Shapiro D.J et al [293] | 2021 | Short- Versus Prolonged-Duration Antibiotics for Outpatient Pneumonia in Children | ObsR | USA | Outpatient | • | short-vs prolonged-duration antibiotics. | Compared with the prolonged-duration group, the aORs for hospitalization, new antibiotic prescriptions, and acute care visits in the short-duration group were 1.16 (95% CI 0.80-1.66), 0.93 (95% CI 0.85-1.01), and 1.06 (95% CI 0.98-1.15), respectively. |
| Al-Tawfiq et al [294] | 2017 | A multifaceted approach to decrease inappropriate antibiotic use in a pediatric outpatient clinic | ObsP | SAU | Primary Care | •• | Educational grand round, academic detailing and prospective auditi and feedback and peer comparison | The monthly rate of prescriptions of inappropriate antibiotics significantly decreased from 12.3% to 3.8% |
| Blair et al ["95] | 2023 | Multi-faceted intervention to improve management of antibiotics for children presenting to primary care with acute cough and respiratory tract infection (CHICO): efficient cluster randomised controlled trial. | RCT | GBR | Primary care | ••• | Elicitation of parental concerns during consultation; a clinician focused prognostic algorithm to identify children at very low, normal, or elevated 30 day risk of hospital admission accompanied by antibiotic prescribing guidance; and a leaflet for carers including safety netting advice. | No evidence was found that antibiotic dispensing differed between intervention practices (155 (95% confidence interval 138 to 174) items/year/1000 children) and control practices (157 (140 to 176) items/year/1000 children) (rate ratio 1.011, 95% confidence interval 0.992 to 1.029; P=0.25). Pre-specified sensitivity analysis suggested reduced dispensing among older children in the intervention arm (P=0.03). |
| Bourgeois et al [296] | 2010 | Impact of a Computerized Template on Antibiotic Prescribing for Acute Respiratory Infections in Children and Adolescents | RT | USA | Primary Care | • | ARI-IT within an EHR to manage pediatric ARIs | When using CDS, AP for acute respiratory illness were significantly reduced (31.7% vs 39.9%; p = 0.02) as the use of macrolides (6.2% vs 9.5%; p = 0.02) |
| Clegg et al [297] | 2021 | Improving antibiotic prescribing for pediatric acute respiratory tract infections: A cluster randomized trial to evaluate individual versus clinic feedback | RCT | USA | Primary care | ••• | 1-hour, in-person, educational session on clinical guidelines for URTI, ABS, and AOM. A tip sheet detailing how to improve scores, an after-visit summary | Both intervention and control groups demonstrated improvement from baseline, but the intervention group had significantly greater improvement compared with the control group: URTI (odds ratio [OR], 1.62; 95% confidence interval [CI], 1.37–1.92; P < 0.01); ABS (OR, 1.45; 95% CI, 1.11–1.88; P < 0.01); and AOM (OR, 1.59; 95% CI, 1.24–2.03; P < 0.01). The intervention group also showed significantly greater reduction in broad-spectrum antibiotic prescribing percentage (BSAP%): odds ratio 0.80, 95% CI 0.74-0.87, P < 0.01. During the postintervention year, gains were maintained in the intervention group for each ARTI and for URI and AOM in the control group. |
| Clegg et al [298] | 2019 | Impact of Education and Peer Comparison on Antibiotic Prescribing for Pediatric Respiratory Tract Infections | BA | USA | Primary Care | •••• | Educational materials, clinical guidelines in June 2014, For the collaborative clinics a 1 hour, in person educational visit was held with each clinic's lead clinician and clinic administrator to discuss the clinical guidelines, measure definitions, baseline performance scores and imprevoment methods in September 2014, Perfomance feedback since September 2014, Non productive compensation, tip sheet emailed monthly for all clinics with clinic-specific feedback | Collaborative clinics had baseline medians for appropriate or first lime treatment of 70% for URI, 53% for ABS and 36% for AOM. To reach targets for URI, ABS, AOM required 6, 14 and 18 moths respectively. At 42 months performance for all 3 ARTIs remained >90%. BASP% decreased from baseline of 57% to 34% ad 24 months. |
| Cohen et al [299] | 2022 | Pediatric Antibiotic Stewardship for Community-Acquired Pneumonia: A Pre-Post Intervention Study | BA | ISR | Primary care | • | 1-day seminar for primary care pediatricians on the diagnosis and treatment of CAP | Substantial decrease in the use of azithromycin after the intervention. In younger children, there was a 42% decrease, alongside an increased use of amoxicillin (P < .001). In older children, there was a smaller, non-statistically significant decrease in the use of azithromycin (P = .45). |
| Di Mario et al [£00] | 2018 | Observational pre–post study showed that a quality improvement project reduced paediatric antibiotic prescribing rates in primary care | BA | ITA | Primary Care | •••• | Developing guidelines and updates, disseminating evidence, audits and feedback, public information campaigns, engaging health managers and performarce incentives | The total prescription rate declined over time (p <0.001), by 33%. The ratio of amoxicillin to amoxicillin clavulanate rose significantly (p = 0.001) by 78%, from 0.6 to 1.1 |
| Diaz et al [301] | 2020 | Impact of a Personalized Audit and Feedback Intervention on Antibiotic Prescribing Practices for Outpatient Pediatric Community-Acquired Pneumonia | RCT | USA | Primary Care | •• | Educational webinar, Personalized audit and feedback monthly only for intervention group | In patients > 5 years, the intervention group has fewer non -guideline-concordant antibiotics prescribed (22/103 [21,4%] control; 3/51 [5,9%] intervention, p<0,05) and received more of the guideline-concordant antibiotics |
| Fiks et al [302] | 2015 | Adoption of Electronic Medical Record-Based Decision Support for Otitis Media in Children | RCT | USA | Primary Care | •• | CDS and performance feedback | Clinicians who received performance feedbacks had a relative increase in CDS use of 9.0 percentage points compared to others (p = 0.001). For AOM, there was a 5.4 percentage point relative increase in use of amoxicillin as a first-line therapy and a 4.9 percentage point increase in the prescribing of an appropriate antibiotic for penicillin-allergic patients. In addition, there was a 17.0 percentage point relative increase in prescribing of high-dose amoxicillin |
| Finkelstein et al [303] | 2008 | Impact of a 16-Community Trial to Promote Judicious Antibiotic Use in Massachusetts | RCT | USA | Primary Care | •••• | Guideline dissemination, small group education, frequent updates and educational materials and prescribing feedback; parents received educational materials by mail and in primary care practices, pharmacies and child care settings. | There was a 4.2 % decrease in antibiotic prescription among children aged 24 to <48 months and 6.7% decrease among those aged 48 to <72 months |
| Forrest et al [304] | 2013 | Improving Adherence to Otitis Media Guidelines With Clinical Decision Support and Physician Feedback | RCT | USA | Primary Care | •• | CDS and performance feedback | The increase from baseline to intervention periods in adherence to guidelines was larger for CDS compared with non-CDS visits |
| Francis et al [305] | 2009 | Effect of using an interactive booklet about childhood respiratory tract infections in primary care consultations on reconsulting and antibiotic prescribing: a cluster randomised controlled trial | RCT | GBR | Primary Care | •••• | Booklet for clinician and parents | Antibiotics were prescribed at the index consultation to 19.5% of children in the intervention group and 40.8% of children in the control group (absolute RR 21.3%, 95% CI 13.7 to 28.9), p<0.001) |
| Frost et al [306] | 2021 | Improving delayed antibiotic prescribing for acute otitis media | BA | USA | Primary care | •• | Educational sessions (in-person and virtual sessions), audit and feedback, online resources, and content expertise. | The rate of delayed antibiotic prescribing increased from 2% at baseline to 21% at intervention end (RRR: 8.96; 95% confidence interval [CI]: 4.68-17.17). Five practices submitted postintervention data. The rate of delayed prescribing at 3 months and 6 months postintervention remained significantly higher than baseline (3 months postintervention, RRR: 8.46; 95% CI: 4.18-17.11; 6 months postintervention, RRR: 6.69; 95% CI: 3.53-12.65) and did not differ from intervention end (3 months postintervention, RRR: 1.12; 95% CI: 0.62-2.05; 6-months postintervention, RRR: 0.89; 95% CI: 0.53-1.49). |
| Gagliotti et al [307] | 2015 | A regionwide intervention to promote appropriate antibiotic use in children reversed trends in erythromycin resistance to Streptococcus pyogenes | BA | ITA | Primary Care | • | Guideline for the management of acute pharyngitis | The outpatient APR showed a decrease of 14% over the seven-year period. The use of macrolides decreased by 24% in 2007 and 2013 (p < 0.001).The use of macrolides in children with at least one pharyngeal isolation of *S.pyogenes* during the year decreased by 28% (p < 0.001) and erythromycin resistance significantly declined from 23% to 9% (p < 0.001) |
| Gerber et al [39] | 2013 | Effect of an Outpatient Antimicrobial Stewardship Intervention on Broad-Spectrum Antibiotic Prescribing by Primary Care Pediatricians A randomized trial | RCT | USA | Primary Care | •• | One 1-hour on-site clinician education session followed by 1 year of personalized, quarterly audut and feedback of prescribing for bacterial and viral ARTIs or usual practice | Broad-spectrum antibiotic prescription decreased from 26.8% to 14.3% vs from 28.4% to 22.6% in controls. CAP off-guideline prescribing decreased from 15.7% to 4.2% among intervention practices compared with 17.1% to 16.3% in controls. Acute sinusitis off-guideline prescribing decreased from 38.9% to 18.8% in intervention practices and from 40.0% to 33.9% in controls. Off-guideline prescribing was uncommon at baseline and changed little for GAS pharyngitis and for viral infections |
| Gulliford et al [308] | 2019 | Effectiveness and safety of electronically delivered prescribing feedback and decision support on antibiotic use for respiratory illness in primary care: REDUCE cluster randomised trial | RCT | GBR | Primary Care | •• | AMS intervention comprised a brief training webinar, automated monthly feedback of antibiotic prescribing and electronic decision supporto tools to inform appropriate prescribing over 12 months. | The adjusted rate ratio for antibiotic prescribing for ARTI was 0,88 (95% CI 0,78 to 0,99, p = 0,04). Antibiotic prescribing was reduced most in adults aged 15 - 84 years . There was no evidence of effect for children younger than 15 years (adjusted rate ratio 0,96, 95% CI 0,82-1,12) |
| Hersh et al [209] | 2018 | Impact of Antimicrobial Stewardship for Pediatric Outpatient Parenteral Antibiotic Therapy | BA | USA | Primary Care | • | 1) stewardship team review/reccomendations included input regarding discharge with OPAT prescription, 2) the peripherally inserted centrale catheter team paged the stewardiship team before line placement if the designated purpose was OPAT, 3) care coordinators paged the stewardship team when arranging home care for OPAT, 4) discharge planning software was modified to generate an electronic alert via text message to the stewardhisp team when discharge medications included OPAT | Introduction of the program was associated with a 24% overall (6,7% monthly) reduction in outpatient parenteral antibiotic therapy use. Most specifically, a reduction in OPAT use was observed for conditions in which evidence supports the use of oral therapy in place of intravenous therapy (eg osteoraticula and respiratory infections) |
| Hurlimann et al [310] | 2015 | Improvement of antibiotic prescription in outpatient care: a cluster-randomized intervention study using a sentinel surveillance network of physicians | RCT | CHE | Primary Care | *••* | Providing guidelines on treatment of ARTIs and UTIs coupled with sustained regular feedback on individual antibiotic prescription behaviour during 2 years | Implementing guidelines coupled with sustained individual feedback was not able to reduce the proportion of sinusitis and otherA RTIs rteated with antibiotics but increased the use of recommended antibiotics for ARTIs and UTIs. The effect of intervention was significatly larger in children than in adults. The intervention was less effective in pediatric practices than in general or internal practices. |
| Huynh et al [311] | 2019 | Impact of expanding a paediatric OPAT programme with an antimicrobial stewardship intervention | ObsP | AUS | Primary Care | *••* | OPAT specific guidelines, Active review of OPAT prescription and input by Paediatric Infectious Diseases | Despite the increase in activity between the 2 periods, with the AMS intervention, overall approppriate antibiotic prescribing remained high: 71% versus 76%. Inappropriately long durations reduced from 30/312 (10%) to 37/617 (6%) (OR 0,6, 95% CI 0,4 to 0,99, p=0,04) and median number of days on broad spectrum antibioticts from 11 (IQR 8-24,5) to 8 (IQR 5-11). |
| Iwamoto et al [312] | 2022 | Change in use of pediatric oral antibiotics in Japan, pre- and post-implementation of an antimicrobial resistance action plan | BA | JPN | Primary care | • | AMR action plan | Antimicrobial usage varied with age. Specifically, usage decreased post-AMR in patients aged ≤8 years and increased in those aged >15 years. Further, antimicrobial prescriptions tended to decrease after 2016 in primary care clinics and hospitals. |
| Jindrak et al [313] | 2008 | Improvements in antibiotic prescribing by community paediatricians in the czech republic | BA | CZE | Primary Care | •• | Feedback based on the results of the repeates surveys, Dissemination of printed survey results to individual doctors, Final conference and local seminars | In 2003 was observed a significant decrease of the overall antibiotic consumption, however its qualitative structure remained inappropriate (high consumption of aminopenicillins with beta lactamase inhibitors, macrolides and fluoroquinolones). A rapid increase of resistance to erythromycin from 3% to more than 16% was observed between 1996 and 2000. This dangerous trend was interrupted and the rates fell back to 9% in 2002-2003 probably due to a decrease in macrolide consumption during 2001-2002 |
| Karas et al [314] | 2023 | Utilizing Clinical Decision Support in the Treatment of Urinary Tract Infection across a Large Pediatric Primary Care Network. | BA | USA | Primary care | ••• | Guideline for urinary tract infections + standardized order set provided clinical decision support regarding appropriate first-line antibiotic therapy. | Utilization of the recommended first-line therapy, cephalexin, increased from 27.5% to 74.8%. Over the same period, trimethoprim-sulfamethoxazole, no longer recommended due to high local resistance, decreased from 31.8% to 8.1%. |
| Katz et al [315] | 2022 | Improvements in appropriate ambulatory antibiotic prescribing using a bundled antibiotic stewardship intervention in general pediatrics practices | BA | USA | Primary care | •••• | online tool kit with resources for implementation of outpatient pediatric antimicrobial stewardship, and we paired the tool kit with quarterly peer comparison and monthly practice-specific data review | We detected improvements in guideline-concordant antibiotic use in the pre-COVID-19 intervention period, and they were sustained in the study period during the pandemic (P3): otitis media (P1 72.14% vs P2 81.42% vs P3 86.11%), group A streptococcal pharyngitis (P1 66.13% vs P2 81.56% vs P3 80.44%), pneumonia (P1 70.6% vs P2 76.2% vs P3 100%), sinusitis (P1 76.2% vs P2 83.78% vs P3 82.86%), skin and soft-tissue infections (P1 97.18% vs P2 100% vs P3 100%). |
| Lemiengre et al [316] | 2018 | Point-of-care CRP matters: normal CRP levels reduce immediate antibiotic prescribing for acutely ill children in primary care: a cluster randomized controlled trial | RCT cluster | BEL | Primary Care | •• | POC CRP test | Compared to episodes in which CRP was not tested, the mere perfoming of PCO CRP reduced prescribing in case EBM practice guidelines advise to prescrive antibiotics (aOR 0,54 CI 95% 0,33-0,9). Normal CRP levels reduced antibiotic prescribing, regardless of whether the advice was to prescribed (aOR 0,24 95%CI 0,11-0,5) or to withhold (aOR 0,31, 95% cI 0,17-0,57). Elevaterd CRP levels did not increase antibiotic prescribing |
| Mainous et al [317] | 2013 | Impact of a clinical decision support system on antibiotic prescribing for acute respiratory infections in primary care: quasi-experimental trial | BA | USA | Primary Care | •• | Quaterly EHR based audit and feedback,"best practice" dissemination during meetings of practice representatives and practice site visits for academic detailing, performance review and CDSS training | Decline of 19.7% in broad-spectrum AP versus an increase of 0.9% in control practices |
| Norton et al [318] | 2018 | Improving Guideline-Based Streptococcal Pharyngitis Testing: A Quality Improvement Initiative | BA | USA | Primary Care | ••• | Face to face meeting, Provider education, modification of existing office procedure, email update, communication strategies and patient and family education | An absolute reduction in unnecessary GAS testing of 23.5% (from 64% to 40.5%) was observed during the project. Appropriate antibiotic use for GAS pharyngitis did not significantly change during the project |
| Pagano F et al [319] | 2023 | Reduction in broad-spectrum antimicrobial prescriptions by primary care pediatricians following a multifaceted antimicrobial stewardship program | BA | ITA | Primary care | ••• | Obligation to associate an appropriate International Classification of Diseases-9 code to each antibiotic prescription, the publication of schemes for empirical antibiotic therapy and educational interventions. | From 2016 to 2020 we observed a substantial reduction in both the annual prescription rate per 100 patients (9.33 to 3.39; R 2 = 0.927, p = 0.009). The prescription rates of Amoxicillin-Clavulanate (50.25 to 14.21; R 2 = 0.983, p = 0.001) and 3GCP (28.43 to 5.43; R 2 = 0.995, p < 0.01) significantly decreased. |
| Papaevangelou et al [320] | 2012 | Decrease of Antibiotic Consumption in Children with Upper Respiratory Tract Infections after Implementation of an Intervention Program in Cyprus | BA | CYP | Primary Care | *••* | 1 day workshop on antibiotic misuse in children with URTIs, lectures on antibiotics use and therapeutic algorithms : To educate parents: invitations topartecipate in educational lectures, instructive pamphlets at pediatric offices and emergency rooms, educational video in waiting rooms and a 30 minuttes discussione was broadcasted through the radio and an article was published in the local newspaper | The difference between consumption units indexes pre- and postintervention had a p= 0.008 |
| Puzz L. et al [321] | 2023 | Evaluation of a Pediatric Community-Acquired Pneumonia Antimicrobial Stewardship Intervention at an Academic Medical Center | BA | USA | Primary care | •• | Local pediatric CAP treatment guidelines + Handshake stewardship included prospective audits with feedback and rounding in person | Antibiotic selection significantly improved, with prescriptions for ceftriaxone decreasing (p < 0.001) and ampicillin increasing (p < 0.001) following the interventions. Antibiotic duration decreased from a median of ten days in the pre-intervention group and post-intervention group 1 to eight days in post-intervention group 2 |
| Ray K.N et al [322] | 2021 | Antibiotic Prescribing for Acute Respiratory Tract Infections During Telemedicine Visits Within a Pediatric Primary Care Network | BA | USA | Primary care | •• | learning collaborative videoconferences and sharing of clinic and clinician-level metrics through an interactive dashboard. | Guideline-concordant antibiotic management occurred in 92.5% of telemedicine visits compared to 90.7% of in-person office visits (P = .004). |
| Regev-Yochay et al [323] | 2011 | Reduction in Antibiotic Use Following a Cluster Randomized Controlled Multifaceted Intervention: The Israeli Judicious Antibiotic Prescription Study | RCT | ISR | Primary Care | ••• | Physicians focus group meetings, workshops, seminars, practice campaigns, evidence-based guidelines, pamphlets, posters, coloring bookleets | The decreased overall APR was significantly greater in the intervention group than in the control group (RR, 0.89; 95% CI, 0.81–0.98). Macrolide prescription rates were most markedly reduced (RR, 0.65; 95% CI, 0.52–0.81; p = 0.001). The penicillin prescription rate did not change significantly in either group. The cephalosporin prescription rate was reduced significantly but with no difference between the 2 groups |
| Stille et al [324] | 2008 | Physician Responses to a Community-Level Trial Promoting Judicious Antibiotic Use | RCT | USA | Primary Care | ••• | Locally endorsed guidelines, group educational sessions and biweekly newletters | Intervention group (OR = 2.4; 95% CI, 1.2-4.9) and increasing years in practice (OR = 1.04 for each additional year; 95% CI, 1.00-1.08) were positively associated with reported decreases in use during the study period |
| Torres et al [325] | 2014 | Impact Assessment of a Decision Rule for Using Antibiotics in Pneumonia: A Randomized Trial | RCT | ARG | Primary Care | • | Prediction rule bacterial pneumonia score | The use of antibiotics was significantly lower in the bacterial pneumonia score group (46.6% vs. 86.6, OR 0.13 95% CI: 0.05 - 0.35, p<0,001) |
| Trinh et al [326] | 2020 | Association between National Treatment Guidelines for Upper Respiratory Tract Infections and Outpatient Pediatric Antibiotic Use in France: An Interrupted Time–Series Analysis | ITS | FRA | Primary Care | *•* | Update clinical practice guidelines for managing upper respiratory tract infections (announced on Web sites and communicated through several oral presentations during conferences and workshops both before and after its official release) | The annual antibiotic prescription rate decreased by 33,15% (from 1387 to 928 per 1000 pediatric inhabitants per year), consistently across age groups and major antibiotic agents except for amoxicillin (+14,4%). We observed a gradual increase in the proportion of amoxicillin (relative change 5 years postintervention of +64,3% 95% CI 51,6-80,1 and +28,4% 95% CI 21,1 - 36,2 for children 0-5 and 6-14 years respectively) concomitantly with a gradual decrease in the proportion of broad spectrum antibiotics (relative change 5 years postintervention of -26,1% 95% CI -29,3, -23,7 and -19,8% 95% CI -22,1, -16 for children 0-5 and 6-14 years respectively |
| Wei et al [327] | 2017 | Effect of a training and educational intervention for physicians and caregivers on antibiotic prescribing for upper respiratory tract infections in children at primary care | RCT | CHN | Primary Care + Inpatient | •••• | Evidence-based prescribing guideline, training and monthly prescribing peer-review meetings for doctors, brief educational for caregiver during consultations and an educational waiting room video for caregivers | The APR at the individual level decreased from 82% to 40% in the intervention group, and from 75% to 70% in the control group. The APR difference between the groups represented an intervention effect (absolute RR in antibiotic prescribing) of –29% (95% CI –42 to –16; p=0.0002) |
| Wei et al [42] | 2019 | Long-term outcomes of an educational intervention to reduce antibiotic prescribing for childhood upper respiratory tract infections in rural China: Follow-up of a cluster-randomised controlled trial | RCT | CHN | Primary Care + Inpatient | •••• | Evidence-based prescribing guideline, training and monthly prescribing peer-review meetings for doctors, brief educational for caregiver during consultations and an educational waiting room video for caregivers only for the first 6 months | In intervention facilities the APR was 84% at baseline, 37% ad 6 months and 54% at 18 months and in control facilities it 76%, 77% and 75% respectively. The difference at 18 months represented an 18month intervention-arm reduction in the APR of -36pp (95% CI -55 to -17, p<0,0001) |
| Zhang et al [328] | 2018 | Cost-effectiveness analysis of a multi-dimensional intervention to reduce inappropriate antibiotic prescribing for children with upper respiratory tract infections in China | RCT | CHN | Primary Care + Inpatient | •••• | COincise evidence-based clinical guidelines on URTI management, monthly peer-review meetings assessing providers' antibitioc prescirption rates, Patients and caregivers received information on approppriate antibiotic use, both verbally and via an educational leaflet, A video with key messages on approppriate use of antibiotics was played daily in the waiting rooms and public areas of the town-ship hospital | A 29% reduction in APR was achieved at an average upfront cost of $390.65 per health facility and an incremental cost of $1.02 per patient in the intervention arm compared with the control arm. This produced an ICER of $0.03 per percentage point reduction, meaning the intervention is close to cost-neutral |

***Intervention legend:*** *• = Guidelines, •=Audit and feedback, • = Physicians education, • = Parents education, • = Pre-authorization, • = CDS tool, • = CP, • = Other ASP intervention, • = Diagnostic Stewardship*

**Abbreviations**: 3GCs,Third Generation Cephalosporins; ABS, Acute Bacterial Sinusitis; ACV, acyclovir; AD, admission days; AMS, antimicrobials stewardship; AOM, Acute Otitis Media; aOR, adjusted odd ratios; APR, antibiotic prescription rates; AR-IAI, Appendix-Related Intraabdominal Infections; ARI-IT, Acute respiratory interactive template; ARTI, acute respiratory tract infection; ASP, Antibiotic stewardship program; AST, antimicrobial susceptibility testing; AU, antibiotic use; AUR, antibiotic use rate; BA, Before and after; BD, Bed days; BLI, β-lactamase inhibitors; BPA, best practice advisory; BRPCR, broad range PCR; BTS, British Thoracic Society; CA, complex appendicitis; CAP, Community acquires pneumonia; CDC, Centers for Disease Control and Prevention; CDS, Clinical Decision Support; cfDNA, plasma cell-free DNA; CHOP, Children's Hospital of Philadelphia; CI, Confidence Interval; CLABSI, central line-associated bloodstream infection; CPG, clinical practice guideline; CPOE, computerized physician order entry; CPs, Clinical Pathways; CR-GNB, carbapenem-resistant gram-negative bacteria; CRE, carbapenem-resistant Enterobacteriaceae; CRP, C reactive protein; CSF, cerebrospinal fluid; CXR, Chest X-ray; DDD, Defined daily doses; dDD, Direct disk diffusion testing; DOT, Day of therapy; EBM, evidence based medicine; ED, Emergency Department; EHR, Electronic Health Record; EM, emergency medicine; EMR, electronic medical record; EONI, Early onset neonatal infection; EOS, Early Onset Sepsis; ESBL, Extended Spectrum beta-lactamase; Flu, Influenza; FN, Febrile Neutropenia; GA, Gestational age; GAS, Group A Streptococcus; GIP, gastrointestinal panels; H&P, history and physical; HR, Hazard Ratio; ICER, incremental cost-effectiveness ratio; ICU, Intensive care unit; ID, Infectious diseases; IDSA, Infectious Diseases Society of America; IQR, interquartile range; IRRS, incident rate ratios; ITS, Interrupted Time series; IV, Intravenous; LOC, Slength of critical care stay; LOS, Length of stay; LOT, Length of therapy; LPs, Lumbar punctures; MEP, Meningitis Encephalitis Panel; mNGF, Metagenomic next-generation sequencing; MRSA, methicillin-resistant SA; MSSA, methicillin-susceptible SA; NEC, Necrotizing enterocolitis; NICU, Neonatal Intensive Care Unit; ObsP, Observational Prospective; ObsR, Observational Retrospective; OPAT, outpatient parenteral antimicrobial treatment; OR, Odds ratio; PAP, Perioperative antibiotic prophylaxis; PCR, Polymerase chain reaction; PCT, procalcitonin; PD Patient Day; PDSA, plan-do-study-act cycle; PICU, Pediatric Intensive Care Unit; PID, paediatric infectious disease; PIDS, Pediatric Infectious Diseases Society; PK, pharmacokinetic; POCT, Point of Care Test; QI quality improvement; RDT, Rapid Diagnostic Test; RR, Relative Risk; RRR, Relative Risk Reduction; RSV, Respiratory Syncytial Virus; SA *S. aureus*; SAB, Staphylococcus aureus bacteremia; SAP, surgical antibiotic prophylaxis; SOPs, Standard operating procedures; SPIN, specialist pneumonia intervention nursing; SRC, sepsis risk calculator; SSI, Surgical site infection; SSTI, Skin and Soft tissue infection; TDM, Therapeutic Drug monitoring; UAD, unnecessary antibiotic days; URTI, Upper Respiratory Tract Infections; uUTI, uncomplicated Urinary tract infection; VRE, Vancomycin Resistant Enterococcus; WSPs, wait-and-see prescriptions; RCT, Randomized controlled trial

**References**

1. Andrade A, Bang H, Reddick K, Villaseñor B, Tran NK, May L. Evaluation of pharmacist guided intervention using procalcitonin and respiratory virus testing. Am J Emerg Med. 2023;66:146-151. doi:10.1016/j.ajem.2023.01.041
2. Angoulvant F, Skurnik D, Bellanger H, et al. Impact of implementing French antibiotic guidelines for acute respiratory-tract infections in a paediatric emergency department, 2005-2009. Eur J Clin Microbiol Infect Dis. 2012;31(7):1295-1303. doi:10.1007/s10096-011-1442-4
3. Angoulvant F, Pereira M, Perreaux F, et al. Impact of unlabeled French antibiotic guidelines on antibiotic prescriptions for acute respiratory tract infections in 7 Pediatric Emergency Departments, 2009-2012. Pediatr Infect Dis J. 2014;33(3):330-333. doi:10.1097/INF.0000000000000125
4. Aronson PL, Thurm C, Williams DJ, et al. Association of clinical practice guidelines with emergency department management of febrile infants ≤56 days of age. J Hosp Med. 2015;10(6):358-365. doi:10.1002/jhm.2329
5. Baer G, Baumann P, Buettcher M, et al. Procalcitonin guidance to reduce antibiotic treatment of lower respiratory tract infection in children and adolescents (ProPAED): a randomized controlled trial. PLoS One. 2013;8(8):e68419. Published 2013 Aug 6. doi:10.1371/journal.pone.0068419
6. Bird C, Winzor G, Lemon K, Moffat A, Newton T, Gray J. A Pragmatic Study to Evaluate the Use of a Rapid Diagnostic Test to Detect Group A Streptococcal Pharyngitis in Children With the Aim of Reducing Antibiotic Use in a UK Emergency Department. Pediatr Emerg Care. 2021;37(5):e249-e251. doi:10.1097/PEC.0000000000001560
7. Crook J, Xu M, Slaughter JC, et al. Impact of clinical guidance and rapid molecular pathogen detection on evaluation and outcomes of febrile or hypothermic infants. Infect Control Hosp Epidemiol. 2020;41(11):1285-1291. doi:10.1017/ice.2020.317
8. Cunney R, Kirrane-Scott M, Rafferty A, Stapleton P, Okafor I, McNamara R. 'Start smart': using front-line ownership to improve the quality of empiric antibiotic prescribing in a paediatric hospital. BMJ Open Qual. 2019;8(3):e000445. Published 2019 Aug 19. doi:10.1136/bmjoq-2018-000445
9. Daggett A, Wyly DR, Stewart T, et al. Improving Emergency Department Use of Safety-Net Antibiotic Prescriptions for Acute Otitis Media. Pediatr Emerg Care. 2022;38(3):e1151-e1158. doi:10.1097/PEC.0000000000002525
10. Demirjian A, Bustinduy AL, Ladhani S, Iqbal Y, Sharland M. Implementation of a Highly Accurate Rapid Point-of-Care Test for Group a Streptococcus Detection at a Large Pediatric Emergency Department in South London. Pediatr Infect Dis J. 2019;38(8):e183-e185. doi:10.1097/INF.0000000000002284
11. Dona D, Baraldi M, Brigadoi G, et al. The Impact of Clinical Pathways on Antibiotic Prescribing for Acute Otitis Media and Pharyngitis in the Emergency Department. Pediatr Infect Dis J. 2018;37(9):901-907. doi:10.1097/INF.0000000000001976
12. Dube AR, Zhao AR, Odozor CU, et al. Improving Prescribing for Otitis Media in a Pediatric Emergency Unit: A Quality Improvement Initiative. Pediatr Qual Saf. 2023;8(1):e625. Published 2023 Jan 16. doi:10.1097/pq9.0000000000000625
13. Free RC, Richardson M, Pillay C, et al. Specialist pneumonia intervention nurse service improves pneumonia care and outcome. BMJ Open Respir Res. 2021;8(1):e000863. doi:10.1136/bmjresp-2020-000863
14. Geurts DH, Vos W, Moll HA, Oostenbrink R. Impact analysis of an evidence-based guideline on diagnosis of urinary tract infection in infants and young children with unexplained fever. Eur J Pediatr. 2014;173(4):463-468. doi:10.1007/s00431-013-2182-5
15. Gomez B, Fernandez-Uria A, Benito J, Lejarzegi A, Mintegi S. Impact of the Step-by-Step on febrile infants. Arch Dis Child. 2021;106(11):1047-1049. doi:10.1136/archdischild-2021-322475
16. Grandjean-Blanchet C, Le CK, Villeneuve S, et al. Value-Based Care for Healthy Children With First Episode of Febrile Neutropenia. Hosp Pediatr. 2023;13(6):536-544. doi:10.1542/hpeds.2022-007075
17. Hamner M, Nedved A, Austin H, et al. Improving Duration of Antibiotics for Skin and Soft-tissue Infections in Pediatric Urgent Cares. Pediatrics. 2022;150(6):e2022057974. doi:10.1542/peds.2022-057974
18. Kooner GK, Bass M, Saroha V, Gonzalez PJ, Jain S. Reducing Antibiotic Duration for Uncomplicated UTI in the Pediatric Emergency Department. Hosp Pediatr. 2024;14(4):265-271. doi:10.1542/hpeds.2023-007561
19. Mercurio L, Hill R, Duffy S, Zonfrillo MR. Clinical Practice Guideline Reduces Evaluation and Treatment for Febrile Infants 0 to 56 Days of Age. Clin Pediatr (Phila). 2020;59(9-10):893-901. doi:10.1177/0009922820920933
20. Nedved A, Lee BR, Hamner M, Wirtz A, Burns A, El Feghaly RE. Impact of an antibiotic stewardship program on antibiotic choice, dosing, and duration in pediatric urgent cares. Am J Infect Control. 2023;51(5):520-526. doi:10.1016/j.ajic.2022.07.027
21. Nedved A, Fung M, Bizune D, et al. A Multisite Collaborative to Decrease Inappropriate Antibiotics in Urgent Care Centers. Pediatrics. 2022;150(1):e2021051806. doi:10.1542/peds.2021-051806
22. Otake S, Kusama Y, Tsuzuki S, et al. Comparing the effects of antimicrobial stewardship at primary emergency centers. Pediatr Int. 2023;65(1):e15614. doi:10.1111/ped.15614
23. Powell SL, Liebelt E. Appropriate use of vancomycin in a pediatric emergency department through the use of a standardized electronic guideline. J Pediatr Nurs. 2015;30(3):494-497. doi:10.1016/j.pedn.2014.12.014
24. Shishido A, Otake S, Kimura M, et al. Effects of a nudge-based antimicrobial stewardship program in a pediatric primary emergency medical center. Eur J Pediatr. 2021;180(6):1933-1940. Doi:10.1007/s00431-021-03979-3
25. van de Maat JS, Garcia Perez D, Driessen GJA, et al. The influence of chest X-ray results on antibiotic prescription for childhood pneumonia in the emergency department. Eur J Pediatr. 2021;180(9):2765-2772. doi:10.1007/s00431-021-03996-2
26. van de Maat JS, Peeters D, Nieboer D, et al. Evaluation of a clinical decision rule to guide antibiotic prescription in children with suspected lower respiratory tract infection in The Netherlands: A stepped-wedge cluster randomised trial. PLoS Med. 2020;17(1):e1003034. Published 2020 Jan 31. doi:10.1371/journal.pmed.1003034
27. Walters EM, D'Auria J, Jackson C, Walsh-Kelly C, Park D, Willis ZI. An Ambulatory Antimicrobial Stewardship Initiative to Improve Diagnosis and Treatment of Urinary Tract Infections in Children. Jt Comm J Qual Patient Saf. 2019;45(12):829-837. doi:10.1016/j.jcjq.2019.08.004
28. Weddle G, Goldman J, Myers A, Newland J. Impact of an Educational Intervention to Improve Antibiotic Prescribing for Nurse Practitioners in a Pediatric Urgent Care Center. J Pediatr Health Care. 2017;31(2):184-188. doi:10.1016/j.pedhc.2016.07.005
29. Williams DJ, Martin JM, Nian H, et al. Antibiotic clinical decision support for pneumonia in the ED: A randomized trial. J Hosp Med. 2023;18(6):491-501. doi:10.1002/jhm.13101
30. Ambroggio L, Thomson J, Murtagh Kurowski E, et al. Quality improvement methods increase appropriate antibiotic prescribing for childhood pneumonia. Pediatrics. 2013;131(5):e1623-e1631. doi:10.1542/peds.2012-2635
31. Donà D, Zingarella S, Gastaldi A, et al. Effects of clinical pathway implementation on antibiotic prescriptions for pediatric community-acquired pneumonia. PLoS One. 2018;13(2):e0193581. Published 2018 Feb 28. doi:10.1371/journal.pone.0193581
32. Doyon S, Perreault M, Marquis C, et al. Quantitative evaluation of a clinical intervention aimed at changing prescriber behaviour in response to new guidelines. J Eval Clin Pract. 2009;15(6):1111-1117. doi:10.1111/j.1365-2753.2009.01259.x
33. McDaniel CE, Haaland W, Parlaman J, Zhou C, Desai AD. A Multisite Intervention for Pediatric Community-acquired Pneumonia in Community Settings. Acad Emerg Med. 2018;25(8):870-879. doi:10.1111/acem.13405
34. Rutman L, Wright DR, OʼCallaghan J, et al. A Comprehensive Approach to Pediatric Pneumonia: Relationship Between Standardization, Antimicrobial Stewardship, Clinical Testing, and Cost. J Healthc Qual. 2017;39(4):e59-e69. doi:10.1097/JHQ.0000000000000048
35. Yeo YL, O'Brien S, Bear N, Borland ML. Knowledge translation in Western Australia tertiary paediatric emergency department: An audit cycle of effectiveness of guideline dissemination on bronchiolitis management. J Paediatr Child Health. 2020;56(9):1358-1364. doi:10.1111/jpc.14930
36. Aoybamroong N, Kantamalee W, Thadanipon K, Techasaensiri C, Malathum K, Apiwattanakul N. Impact of an Antibiotic Stewardship Program on Antibiotic Prescription for Acute Respiratory Tract Infections in Children: A Prospective Before-After Study. Clin Pediatr (Phila). 2019;58(11-12):1166-1174. doi:10.1177/0009922819870248
37. Di Pietro P, Della Casa Alberighi O, Silvestri M, et al. Monitoring adherence to guidelines of antibiotic use in pediatric pneumonia: the MAREA study. Ital J Pediatr. 2017;43(1):113. Published 2017 Dec 22. doi:10.1186/s13052-017-0432-2
38. March-López P, Madridejos R, Tomas R, et al. Impact of a Multifaceted Antimicrobial Stewardship Intervention in a Primary Health Care Area: A Quasi-Experimental Study. Front Pharmacol. 2020;11:398. Published 2020 Apr 2. doi:10.3389/fphar.2020.00398
39. Poole NM, Kronman MP, Rutman L, et al. Improving Antibiotic Prescribing for Children With Urinary Tract Infection in Emergency and Urgent Care Settings. Pediatr Emerg Care. 2020;36(6):e332-e339. doi:10.1097/PEC.0000000000001342
40. Saha D, Patel J, Buckingham D, Thornton D, Barber T, Watson JR. Urine Culture Follow-up and Antimicrobial Stewardship in a Pediatric Urgent Care Network. Pediatrics. 2017;139(4):e20162103. doi:10.1542/peds.2016-2103
41. Shaw R, Popovsky E, Abo A, et al. Improving antibiotic prescribing in the emergency department for uncomplicated community-acquired pneumonia. World J Emerg Med. 2020;11(4):199-205. doi:10.5847/wjem.j.1920-8642.2020.04.001
42. Widmer K, Schmidt S, Bakel LA, Cookson M, Leonard J, Tyler A. Use of Procalcitonin in a Febrile Infant Clinical Pathway and Impact on Infants Aged 29 to 60 Days. Hosp Pediatr. 2021;11(3):223-230. doi:10.1542/hpeds.2020-000380
43. Alfraij A, Abdelmoniem A, Elseadawy M, et al. The effect of Telehealth Antimicrobial Stewardship Program (Tele-ASP) on antimicrobial use in a pediatric intensive care unit: Pre- and post-implementation single center study. J Infect Public Health. 2023;16(9):1361-1367. doi:10.1016/j.jiph.2023.06.010
44. Achten NB, Dorigo-Zetsma JW, van der Linden PD, van Brakel M, Plötz FB. Sepsis calculator implementation reduces empiric antibiotics for suspected early-onset sepsis. Eur J Pediatr. 2018;177(5):741-746. doi:10.1007/s00431-018-3113-2
45. Acuña M, Benadof D, Yohannessen K, Leiva Y, Clement P. FilmArray® Meningoencephalitis panel in the diagnosis of central nervous system infections: stewardship and cost analysis in a paediatric hospital in Chile. BMC Pediatr. 2022;22(1):182. Published 2022 Apr 5. doi:10.1186/s12887-022-03241-1
46. Adams SM, Ngo L, Morphew T, Babbitt CJ. Does an Antimicrobial Time-Out Impact the Duration of Therapy of Antimicrobials in the PICU?. Pediatr Crit Care Med. 2019;20(6):560-567. doi:10.1097/PCC.0000000000001925
47. Agwu AL, Lee CK, Jain SK, et al. A World Wide Web-based antimicrobial stewardship program improves efficiency, communication, and user satisfaction and reduces cost in a tertiary care pediatric medical center. Clin Infect Dis. 2008;47(6):747-753. doi:10.1086/591133
48. Akangire G, Simpson E, Weiner J, Noel-MacDonnell J, Petrikin J, Sheehan M. Implementation of the Neonatal Sepsis Calculator in Early-Onset Sepsis and Maternal Chorioamnionitis. Adv Neonatal Care. 2020;20(1):25-32. doi:10.1097/ANC.0000000000000668
49. Akter SF, Heller RD, Smith AJ, Milly AF. Impact of a training intervention on use of antimicrobials in teaching hospitals. J Infect Dev Ctries. 2009;3(6):447-451. Published 2009 Jul 1. doi:10.3855/jidc.416
50. Alejandre C, Balaguer M, Guitart C, et al. Procalcitonin-guided protocol decreased the antibiotic use in paediatric patients with severe bronchiolitis. Acta Paediatr. 2020;109(6):1190-1195. doi:10.1111/apa.15148
51. Aljassim NA, Noël KC, Maratta C, et al. Antimicrobial Stewardship in Bronchiolitis: A Retrospective Cohort Study of Three PICUs in Canada. Pediatr Crit Care Med. 2022;23(3):160-170. doi:10.1097/PCC.0000000000002834
52. Arora V, Strunk D, Furqan SH, et al. Optimizing antibiotic use for early onset sepsis: A tertiary NICU experience. J Neonatal Perinatal Med. 2019;12(3):301-312. doi:10.3233/NPM-180075
53. Astorga MC, Piscitello KJ, Menda N, et al. Antibiotic Stewardship in the Neonatal Intensive Care Unit: Effects of an Automatic 48-Hour Antibiotic Stop Order on Antibiotic Use. J Pediatric Infect Dis Soc. 2019;8(4):310-316. doi:10.1093/jpids/piy043
54. Bauer SC, Kaeppler C, Soung P, Porada K, Bushee G, Havens PL. Using Electronic Health Record Tools to Decrease Antibiotic Exposure in Infant Sepsis Evaluation. Hosp Pediatr. 2021;11(9):936-943. doi:10.1542/hpeds.2021-005883
55. Beavers JB, Bai S, Perry J, Simpson J, Peeples S. Implementation and Evaluation of the Early-Onset Sepsis Risk Calculator in a High-Risk University Nursery. Clin Pediatr (Phila). 2018;57(9):1080-1085. doi:10.1177/0009922817751337
56. Berild D, Abrahamsen TG, Andresen S, et al. A controlled intervention study to improve antibiotic use in a Russian paediatric hospital. Int J Antimicrob Agents. 2008;31(5):478-483. doi:10.1016/j.ijantimicag.2008.01.009
57. Berrondo C, Carone M, Katz C, Kenny A. Adherence to Perioperative Antibiotic Prophylaxis Recommendations and Its Impact on Postoperative Surgical Site Infections. Cureus. 2022;14(6):e25859. Published 2022 Jun 11. doi:10.7759/cureus.25859
58. Bobillo-Perez S, Sole-Ribalta A, Balaguer M, et al. Procalcitonin to stop antibiotics after cardiovascular surgery in a pediatric intensive care unit-The PROSACAB study. PLoS One. 2019;14(9):e0220686. Published 2019 Sep 18. doi:10.1371/journal.pone.0220686
59. Borzęcka B, Krasuski K, Kuchar EP. Antibiotic usage at a clinical paediatric hospital before and after the implementation of actions related to the hospital antibiotic policy. Eur J Hosp Pharm. 2021;28(4):207-211. doi:10.1136/ejhpharm-2019-001984
60. Cantey JB, Wozniak PS, Pruszynski JE, Sánchez PJ. Reducing unnecessary antibiotic use in the neonatal intensive care unit (SCOUT): a prospective interrupted time-series study. Lancet Infect Dis. 2016;16(10):1178-1184. doi:10.1016/S1473-3099(16)30205-5
61. Cantey JB, Correa CC, Dugi DD, Huff E, Olaya JE, Farner R. Remote Stewardship for Medically Underserved Nurseries: A Stepped-Wedge, Cluster Randomized Study. Pediatrics. 2022;149(5):e2021055686. doi:10.1542/peds.2021-055686
62. Caruso TJ, Wang E, Schwenk HT, et al. A quality improvement initiative to optimize dosing of surgical antimicrobial prophylaxis. Paediatr Anaesth. 2017;27(7):702-710. doi:10.1111/pan.13137
63. Ceradini J, Tozzi AE, D'Argenio P, et al. Telemedicine as an effective intervention to improve antibiotic appropriateness prescription and to reduce costs in pediatrics. Ital J Pediatr. 2017;43(1):105. Published 2017 Nov 17. doi:10.1186/s13052-017-0423-3
64. Chan S, Hossain J, Di Pentima MC. Implications and impact of prior authorization policy on vancomycin use at a tertiary pediatric teaching hospital. Pediatr Infect Dis J. 2015;34(5):506-508. doi:10.1097/INF.0000000000000615
65. Chiotos K, Fitzgerald JC, Hayes M, et al. Improving Vancomycin Stewardship in Critically Ill Children. Pediatrics. 2022;149(4):e2021052165. doi:10.1542/peds.2021-052165
66. Chiu CH, Michelow IC, Cronin J, Ringer SA, Ferris TG, Puopolo KM. Effectiveness of a guideline to reduce vancomycin use in the neonatal intensive care unit. Pediatr Infect Dis J. 2011;30(4):273-278. doi:10.1097/INF.0b013e3182011d12
67. Ciofi Degli Atti M, Alegiani SS, Raschetti R, et al. A collaborative intervention to improve surgical antibiotic prophylaxis in children: results from a prospective multicenter study. Eur J Clin Pharmacol. 2017;73(9):1141-1147. doi:10.1007/s00228-017-2270-y
68. Coggins SA, Wynn JL, Hill ML, et al. Use of a computerized C-reactive protein (CRP) based sepsis evaluation in very low birth weight (VLBW) infants: a five-year experience. PLoS One. 2013;8(11):e78602. Published 2013 Nov 11. doi:10.1371/journal.pone.0078602
69. Colletti AA, Wang E, Marquez JL, et al. A multifaceted quality improvement project improves intraoperative redosing of surgical antimicrobial prophylaxis during pediatric surgery. Paediatr Anaesth. 2019;29(7):705-711. doi:10.1111/pan.13651
70. Cotter JM, Thomas J, Birkholz M, Ambroggio L, Holstein J, Dominguez SR. Clinical Impact of a Diagnostic Gastrointestinal Panel in Children. Pediatrics. 2021;147(5):e2020036954. doi:10.1542/peds.2020-036954
71. Cowart MC, Miller D, Laham FR, Jordan-Villegas A. Implementation of an Automatic 48-Hour Vancomycin Hard-Stop in a Pediatric Community Hospital. J Pediatr Pharmacol Ther. 2022;27(2):147-150. doi:10.5863/1551-6776-27.2.147
72. Cunningham ME, Zhu H, Hoch CT, et al. Effectiveness of a clinical pathway for pediatric complex appendicitis based on antibiotic stewardship principles. J Pediatr Surg. 2020;55(6):1026-1031. doi:10.1016/j.jpedsurg.2020.02.045
73. Andre Ricardo Araujo da Silva and Deborah Cardoso Albernaz de Almeida Dias. Quality Analysis of Antimicrobial Restriction Policy in Pediatrics. Arch Pediatr Infect Dis. 2021 April; 9(2):e100986. doi: 10.5812/pedinfect.100986
74. Dassner AM, Girotto JE. Evaluation of a Second-Sign Process for Antimicrobial Prior Authorization. J Pediatric Infect Dis Soc. 2018;7(2):113-118. doi:10.1093/jpids/pix015
75. Di Pentima MC, Chan S, Eppes SC, Klein JD. Antimicrobial prescription errors in hospitalized children: role of antimicrobial stewardship program in detection and intervention. Clin Pediatr (Phila). 2009;48(5):505-512. doi:10.1177/0009922808330774
76. Di Pentima MC, Chan S. Impact of antimicrobial stewardship program on vancomycin use in a pediatric teaching hospital. Pediatr Infect Dis J. 2010;29(8):707-711. doi:10.1097/INF.0b013e3181d683f8
77. Di Pentima MC, Chan S, Hossain J. Benefits of a pediatric antimicrobial stewardship program at a children's hospital. Pediatrics. 2011;128(6):1062-1070. doi:10.1542/peds.2010-3589
78. Dimopoulou A, Kourlaba G, Psarris A, Coffin S, Spoulou V, Zaoutis T. Perioperative antimicrobial prophylaxis in pediatric patients in Greece: Compliance with guidelines and impact of an educational intervention. J Pediatr Surg. 2016;51(8):1307-1311. doi:10.1016/j.jpedsurg.2015.11.017
79. Dommett R, Geary J, Freeman S, et al. Successful introduction and audit of a step-down oral antibiotic strategy for low risk paediatric febrile neutropaenia in a UK, multicentre, shared care setting. Eur J Cancer. 2009;45(16):2843-2849. doi:10.1016/j.ejca.2009.06.003
80. Donà D, Luise D, La Pergola E, et al. Effects of an antimicrobial stewardship intervention on perioperative antibiotic prophylaxis in pediatrics. Antimicrob Resist Infect Control. 2019;8:13. Published 2019 Jan 15. doi:10.1186/s13756-019-0464-z
81. Downes KJ, Fitzgerald JC, Schriver E, et al. Implementation of a Pragmatic Biomarker-Driven Algorithm to Guide Antibiotic Use in the Pediatric Intensive Care Unit: the Optimizing Antibiotic Strategies in Sepsis (OASIS) II Study [published correction appears in J Pediatric Infect Dis Soc. 2019 Dec 27;8(6):585. doi: 10.1093/jpids/piz054.]. J Pediatric Infect Dis Soc. 2020;9(1):36-43. doi:10.1093/jpids/piy113
82. Doyon S, Perreault M, Marquis C, et al. Quantitative evaluation of a clinical intervention aimed at changing prescriber behaviour in response to new guidelines. J Eval Clin Pract. 2009;15(6):1111-1117. doi:10.1111/j.1365-2753.2009.01259.x
83. Drwiega, Emily N.; Nichols, Kristen R., Israel, Emily N; Knoderer, Chad A. Impact of Rapid mecA Polymerase Chain Reaction Rapid Diagnostic Testing for Staphylococcus aureus in a Pediatric Setting. Infectious Diseases in Clinical Practice 27(5):p 268-272, September 2019. | DOI: 10.1097/IPC.0000000000000737
84. Dukhovny D, Buus-Frank ME, Edwards EM, et al. A Collaborative Multicenter QI Initiative to Improve Antibiotic Stewardship in Newborns. Pediatrics. 2019;144(6):e20190589. doi:10.1542/peds.2019-0589
85. Esposito S, Tagliabue C, Picciolli I, et al. Procalcitonin measurements for guiding antibiotic treatment in pediatric pneumonia. Respir Med. 2011;105(12):1939-1945. doi:10.1016/j.rmed.2011.09.003
86. Fernández-Polo A, Ramon-Cortes S, Plaja-Dorca J, et al. Impact of an outpatient parenteral antimicrobial treatment (OPAT) as part of a paediatric-specific PROA program. Enferm Infecc Microbiol Clin (Engl Ed). 2023;41(4):230-234. doi:10.1016/j.eimce.2022.08.004
87. Frost HM, Wittmer N, Keith A, Durfee MJ, Jenkins TC. Sustainability of Interventions to Increase Guideline-Concordant Durations of Antibiotic Therapy for Children with Acute Otitis Media. J Pediatr. 2023;253:292-296.e2. doi:10.1016/j.jpeds.2022.09.004
88. Gareau-Terrell J, Branham S. Can Procalcitonin Improve Antibiotic Stewardship for Late-Onset Sepsis Evaluations in Neonates?. Adv Neonatal Care. 2020;20(6):473-478. doi:10.1097/ANC.0000000000000761
89. Gill CJ, Mantaring JB, Macleod WB, et al. Impact of enhanced infection control at 2 neonatal intensive care units in the Philippines. Clin Infect Dis. 2009;48(1):13-21. doi:10.1086/594120
90. Gillon J, Xu M, Slaughter J, Di Pentima MC. Vancomycin Use: Room for Improvement Among Hospitalized Children. J Pharm Pract. 2017;30(3):296-299. doi:10.1177/0897190016635478
91. Goel N, Cannell S, Davies G, et al. Implementation of an adapted Sepsis Risk Calculator algorithm to reduce antibiotic usage in the management of early onset neonatal sepsis: a multicentre initiative in Wales, UK. Arch Dis Child Fetal Neonatal Ed. 2022;107(3):303-310. doi:10.1136/archdischild-2020-321489
92. Goff Z, Abbotsford J, Yeoh DK, et al. The Impact of a Multifaceted Tertiary Pediatric Hospital's Antimicrobial Stewardship Service. Pediatr Infect Dis J. 2022;41(12):959-966. doi:10.1097/INF.0000000000003704
93. Goldman JL, Lee BR, Hersh AL, et al. Clinical diagnoses and antimicrobials predictive of pediatric antimicrobial stewardship recommendations: a program evaluation. Infect Control Hosp Epidemiol. 2015;36(6):673-680. doi:10.1017/ice.2015.45
94. Goldman JL, Newland JG, Price M, Yu D, Lee BR. Clinical impact of an antimicrobial stewardship program on high-risk pediatric patients. Infect Control Hosp Epidemiol. 2019;40(9):968-973. doi:10.1017/ice.2019.198
95. Gong S, Qiu X, Song Y, et al. Effect of Financially Punished Audit and Feedback in a Pediatric Setting in China, within an Antimicrobial Stewardship Program, and as Part of an International Accreditation Process. Front Public Health. 2016;4:99. Published 2016 May 18. doi:10.3389/fpubh.2016.00099
96. Graus JM, Herbozo C, Hernandez R, Pantoja AF, Zegarra J. Managing antibiotics wisely in a neonatal intensive care unit in a low resource setting. J Perinatol. 2022;42(7):965-970. doi:10.1038/s41372-022-01388-4
97. Grewer-Katona G., Hüebner  J., Pecar  A., Wolf G. K. Opportunities for Antibiotic Stewardship Interventions in a Pediatric Hospital, J Pediatr Infect Dis 2022; 17(02): 083-089 DOI: 10.1055/s-0042-1744559
98. Guitart C, Alejandre C, Torrús I, et al. Impact of a modification of the clinical practice guide of the American Academy of Pediatrics in the management of severe acute bronchiolitis in a pediatric intensive care unit. Impacto de una modificación de la guía de práctica clínica de la Academia Americana de Pediatría en el manejo de la bronquiolitis aguda grave en una unidad de cuidados intensivos pediátricos. Med Intensiva (Engl Ed). 2021;45(5):289-297. Doi:10.1016/j.medin.2019.10.006
99. Gustavsson L, Lindquist S, Elfvin A, Hentz E, Studahl M. Reduced antibiotic use in extremely preterm infants with an antimicrobial stewardship intervention. BMJ Paediatr Open. 2020;4(1):e000872. Published 2020 Dec 7. doi:10.1136/bmjpo-2020-000872
100. Hamdy RF, Bhattarai S, Basu SK, et al. Reducing Vancomycin Use in a Level IV NICU. Pediatrics. 2020;146(2):e20192963. doi:10.1542/peds.2019-2963
101. Hersh AL, De Lurgio SA, Thurm C, et al. Antimicrobial stewardship programs in freestanding children's hospitals. Pediatrics. 2015;135(1):33-39. doi:10.1542/peds.2014-2579
102. Holzmann-Pazgal G, Khan AM, Northrup TF, Domonoske C, Eichenwald EC. Decreasing vancomycin utilization in a neonatal intensive care unit. Am J Infect Control. 2015;43(11):1255-1257. doi:10.1016/j.ajic.2015.06.028
103. Horikoshi Y, Higuchi H, Suwa J, Isogai M, Shoji T, Ito K. Impact of computerized pre-authorization of broad spectrum antibiotics in Pseudomonas aeruginosa at a children's hospital in Japan. J Infect Chemother. 2016;22(8):532-535. doi:10.1016/j.jiac.2016.05.001
104. Horikoshi Y, Suwa J, Higuchi H, et al. Sustained pediatric antimicrobial stewardship program with consultation to infectious diseases reduced carbapenem resistance and infection-related mortality. Int J Infect Dis. 2017;64:69-73. doi:10.1016/j.ijid.2017.09.012
105. Horikoshi Y, Kaneko T, Morikawa Y, et al. The North Wind and the Sun: Pediatric Antimicrobial Stewardship Program Combining Restrictive and Persuasive Approaches in Hematology-Oncology Ward and Hematopoietic Stem Cell Transplant Unit. Pediatr Infect Dis J. 2018;37(2):164-168. doi:10.1097/INF.0000000000001746
106. Huebner J, Rack-Hoch AL, Pecar A, Schmid I, Klein C, Borde JP. Pilotprojekt einer pädiatrischen Antibiotic-Stewardship-Initiative am Dr. von Haunerschen Kinderspital - neue Wege der pädiatrischen Infektiologie [Pilot project of a pediatric antibiotic stewardship initiative at the Hauner children's hospital]. Klin Padiatr. 2013;225(4):223-229. doi:10.1055/s-0033-1349063
107. Huetz N, Launay E, Gascoin G, et al. Potential Impact of Umbilical-Cord-Blood Procalcitonin-Based Algorithm on Antibiotics Exposure in Neonates With Suspected Early-Onset Sepsis. Front Pediatr. 2020;8:127. Published 2020 Apr 17. doi:10.3389/fped.2020.00127
108. Hum RS, Cato K, Sheehan B, et al. Developing clinical decision support within a commercial electronic health record system to improve antimicrobial prescribing in the neonatal ICU. Appl Clin Inform. 2014;5(2):368-387. Published 2014 Apr 9. doi:10.4338/ACI-2013-09-RA-0069
109. Hurst AL, Child J, Pearce K, Palmer C, Todd JK, Parker SK. Handshake Stewardship: A Highly Effective Rounding-based Antimicrobial Optimization Service. Pediatr Infect Dis J. 2016;35(10):1104-1110. doi:10.1097/INF.0000000000001245
110. Kalil J, Bowes J, Reddy D, Barrowman N, Le Saux N. Pediatric Inpatient Antimicrobial Stewardship Program Safely Reduces Antibiotic Use in Patients with Bronchiolitis Caused by Respiratory Syncytial Virus: A Retrospective Chart Review. Pediatr Qual Saf. 2019;4(5):e211. Published 2019 Aug 30. doi:10.1097/pq9.0000000000000211
111. Karaali C, Emiroglu M, Atalay S, et al. A new antibiotic stewardship program approach is effective on inappropriate surgical prophylaxis and discharge prescription. J Infect Dev Ctries. 2019;13(11):961-967. Published 2019 Nov 30. doi:10.3855/jidc.11734
112. Karandikar MV, Milliren CE, Zaboulian R, et al. Limiting Vancomycin Exposure in Pediatric Oncology Patients With Febrile Neutropenia May Be Associated With Decreased Vancomycin-Resistant Enterococcus Incidence. J Pediatric Infect Dis Soc. 2020;9(4):428-436. doi:10.1093/jpids/piz064
113. Kashtan M, Dawson M, Anandalwar S, Hills-Dunlap J, Graham DA, Rangel S. Implementation of a Plan-Do-Study-Act framework to reduce unindicated surgical antimicrobial prophylaxis. J Pediatr Surg. 2020;55(1):86-89. doi:10.1016/j.jpedsurg.2019.09.059
114. Katz SE, Crook J, Gillon J, et al. Use of a Procalcitonin-guided Antibiotic Treatment Algorithm in the Pediatric Intensive Care Unit. Pediatr Infect Dis J. 2021;40(4):333-337. doi:10.1097/INF.0000000000002986
115. Ketha B, Stephenson KJ, Dassinger MS 3rd, Smith SD, Burford JM. Eliminating Use of Home Oral Antibiotics in Pediatric Complicated Appendicitis. J Surg Res. 2021;263:151-154. doi:10.1016/j.jss.2020.12.059
116. Khorshidi-Malahmadi I., Sima S., Ansari N., Moghaddas A., Second-year Outcomes of Implementing Antimicrobial Stewardship Program in a Tertiary Pediatric Hospital, Jundishapur Journal of Microbiology: 2021, Vol. 14, issue 8; e118460; <https://doi.org/10.5812/jjm.118460>
117. Kit-Anan W, Boonsathorn S, Anantasit N, Techasaensiri C, Chaisavaneeyakorn S, Apiwattanakul N. Handshake stewardship reduces carbapenem prescription in a pediatric critical care setting. Pediatr Int. 2022;64(1):e15227. doi:10.1111/ped.15227
118. Kitano T, Takagi K, Arai I, et al. A simple and feasible antimicrobial stewardship program in a neonatal intensive care unit of a Japanese community hospital. J Infect Chemother. 2019;25(11):860-865. doi:10.1016/j.jiac.2019.04.012
119. Kopsidas I, Tsopela GC, Molocha NM, et al. Reducing Duration of Antibiotic Use for Presumed Neonatal Early-Onset Sepsis in Greek NICUs. A "Low-Hanging Fruit" Approach. Antibiotics (Basel). 2021;10(3):275. Published 2021 Mar 9. doi:10.3390/antibiotics10030275
120. Kreitmeyr K, von Both U, Pecar A, Borde JP, Mikolajczyk R, Huebner J. Pediatric antibiotic stewardship: successful interventions to reduce broad-spectrum antibiotic use on general pediatric wards. Infection. 2017;45(4):493-504. doi:10.1007/s15010-017-1009-0
121. Kreitmeyr K, Pecar A, Mikolajczyk R, von Both U, Huebner J. Pediatric Antibiotic Stewardship: Optimization of Vancomycin Therapy Based on Individual Pharmacokinetics. Pediatr Infect Dis J. 2021;40(6):556-562. doi:10.1097/INF.0000000000003058
122. Labenne M, Michaut F, Gouyon B, Ferdynus C, Gouyon JB. A population-based observational study of restrictive guidelines for antibiotic therapy in early-onset neonatal infections. Pediatr Infect Dis J. 2007;26(7):593-599. doi:10.1097/INF.0b013e318068b656
123. Laccetta G, Ciantelli M, Tuoni C, Sigali E, Miccoli M, Cuttano A. Early-onset sepsis risk calculator: a review of its effectiveness and comparative study with our evidence-based local guidelines. Ital J Pediatr. 2021;47(1):73. Published 2021 Mar 25. doi:10.1186/s13052-021-01028-1
124. Lamba V, D'souza S, Carafa C, et al. Standardizing the approach to late onset sepsis in neonates through antimicrobial stewardship: a quality improvement initiative. J Perinatol. 2020;40(9):1433-1440. doi:10.1038/s41372-019-0577-5
125. Lanata MM, Diaz A, Hecht SM, et al. Empiric Vancomycin Reduction in a Pediatric Intensive Care Unit. Pediatrics. 2021;148(3):e2020009142. doi:10.1542/peds.2020-009142
126. Lee J, Pai H, Kim YK, et al. Control of extended-spectrum beta-lactamase-producing Escherichia coli and Klebsiella pneumoniae in a children's hospital by changing antimicrobial agent usage policy. J Antimicrob Chemother. 2007;60(3):629-637. doi:10.1093/jac/dkm225
127. Lee KR, Bagga B, Arnold SR. Reduction of Broad-Spectrum Antimicrobial Use in a Tertiary Children's Hospital Post Antimicrobial Stewardship Program Guideline Implementation. Pediatr Crit Care Med. 2016;17(3):187-193. doi:10.1097/PCC.0000000000000615
128. Lee RA, Al Dhaheri F, Pollock NR, Sharma TS. Assessment of the Clinical Utility of Plasma Metagenomic Next-Generation Sequencing in a Pediatric Hospital Population. J Clin Microbiol. 2020;58(7):e00419-20. Published 2020 Jun 24. doi:10.1128/JCM.00419-20
129. LeRiger MM, Phipps AR, Norton BM, Spitznagel RA. Improving the Compliance of Intraoperative Antibiotic Redosing: A Quality Improvement Initiative. Pediatr Qual Saf. 2020;5(2):e285. Published 2020 Apr 10. doi:10.1097/pq9.0000000000000285
130. Naureckas Li C, Nakamura MM. Utility of Broad-Range PCR Sequencing for Infectious Diseases Clinical Decision Making: a Pediatric Center Experience. J Clin Microbiol. 2022;60(5):e0243721. doi:10.1128/jcm.02437-21
131. Liem TY, Van Den Hoogen A, Rademaker CM, Egberts TC, Fleer A, Krediet TG. Antibiotic weight-watching: slimming down on antibiotic use in a NICU. Acta Paediatr. 2010;99(12):1900-1902. doi:10.1111/j.1651-2227.2010.01957.x
132. Lighter-Fisher J, Desai S, Stachel A, Pham VP, Klejmont L, Dubrovskaya Y. Implementing an Inpatient Pediatric Prospective Audit and Feedback Antimicrobial Stewardship Program Within a Larger Medical Center. Hosp Pediatr. 2017;7(9):516-522. doi:10.1542/hpeds.2016-0144
133. Lloyd EC, Martin ET, Dillman N, et al. Impact of a Best Practice Advisory for Pediatric Patients With Staphylococcus aureus Bacteremia. J Pediatric Infect Dis Soc. 2021;10(3):282-288. doi:10.1093/jpids/piaa058
134. Lombardi J, Nguy P, Robichaud Ducharme A, et al. Assessment of Surgical Antibiotic Prophylaxis Compliance in Pediatrics: A Pre-post Quasi-experimental Study. Pediatr Infect Dis J. 2020;39(1):48-53. doi:10.1097/INF.0000000000002490
135. Lu C, Liu Q, Yuan H, Wang L. Implementation of the Smart Use of Antibiotics Program to Reduce Unnecessary Antibiotic Use in a Neonatal ICU: A Prospective Interrupted Time-Series Study in a Developing Country. Crit Care Med. 2019;47(1):e1-e7. doi:10.1097/CCM.0000000000003463
136. MacBrayne CE, Williams MC, Levek C, et al. Sustainability of Handshake Stewardship: Extending a Hand Is Effective Years Later. Clin Infect Dis. 2020;70(11):2325-2332. doi:10.1093/cid/ciz650
137. Malcolmson C, Ng K, Hughes S, et al. Impact of Matrix-Assisted Laser Desorption and Ionization Time-of-Flight and Antimicrobial Stewardship Intervention on Treatment of Bloodstream Infections in Hospitalized Children. J Pediatric Infect Dis Soc. 2017;6(2):178-186. doi:10.1093/jpids/piw033
138. McCarthy KN, Hawke A, Dempsey EM. Antimicrobial stewardship in the neonatal unit reduces antibiotic exposure. Acta Paediatr. 2018;107(10):1716-1721. doi:10.1111/apa.14337
139. McCulloh RJ, Queen MA, Lee B, et al. Clinical Impact of an Antimicrobial Stewardship Program on Pediatric Hospitalist Practice, a 5-Year Retrospective Analysis. Hosp Pediatr. 2015;5(10):520-527. doi:10.1542/hpeds.2014-0250
140. McCulloh RJ, Commers T, Williams DD, Michael J, Mann K, Newland JG. Effect of Combined Clinical Practice Guideline and Electronic Order Set Implementation on Febrile Infant Evaluation and Management. Pediatr Emerg Care. 2021;37(1):e25-e31. doi:10.1097/PEC.0000000000002012
141. McMullan BJ, Mahony M, Java L, et al. Improving intravenous-to-oral antibiotic switch in children: a team-based audit and implementation approach. BMJ Open Qual. 2021;10(1):e001120. doi:10.1136/bmjoq-2020-001120
142. Messacar K, Hurst AL, Child J, et al. Clinical Impact and Provider Acceptability of Real-Time Antimicrobial Stewardship Decision Support for Rapid Diagnostics in Children With Positive Blood Culture Results. J Pediatric Infect Dis Soc. 2017;6(3):267-274. doi:10.1093/jpids/piw047
143. Messacar K, Campbell K, Pearce K, et al. A Handshake From Antimicrobial Stewardship Opens Doors for Infectious Disease Consultations. Clin Infect Dis. 2017;64(10):1449-1452. doi:10.1093/cid/cix139
144. Messacar K, Palmer C, Gregoire L, et al. Clinical and Financial Impact of a Diagnostic Stewardship Program for Children with Suspected Central Nervous System Infection. J Pediatr. 2022;244:161-168.e1. doi:10.1016/j.jpeds.2022.02.002
145. Metjian TA, Prasad PA, Kogon A, Coffin SE, Zaoutis TE. Evaluation of an antimicrobial stewardship program at a pediatric teaching hospital. Pediatr Infect Dis J. 2008;27(2):106-111. doi:10.1097/INF.0b013e318158603a
146. Metz J, Oehler P, Burggraf M, Burdach S, Behrends U, Rieber N. Improvement of Guideline Adherence After the Implementation of an Antibiotic Stewardship Program in a Secondary Care Pediatric Hospital. Front Pediatr. 2019;7:478. Published 2019 Nov 13. doi:10.3389/fped.2019.00478
147. Meyers JM, Tulloch J, Brown K, Caserta MT, D'Angio CT; GOLISANO CHILDREN’S HOSPITAL NICU ANTIBIOTIC STEWARDSHIP TEAM. A Quality Improvement Initiative To Optimize Antibiotic Use in a Level 4 NICU. Pediatrics. 2020;146(5):e20193956. doi:10.1542/peds.2019-3956
148. Miller H, Tseng A, Lowerre T, et al. Improving Time to Stat Intravenous Antibiotic Administration: An 8-Year Quality Initiative. Hosp Pediatr. 2023;13(1):88-94. doi:10.1542/hpeds.2021-006422
149. Miloslavsky M, Galler MF, Moawad I, Actis J, Cummings BM, El Saleeby CM. The Impact of Pediatric-Specific Vancomycin Dosing Guidelines: A Quality Improvement Initiative. Pediatrics. 2017;139(6):e20162423. doi:10.1542/peds.2016-2423
150. Minotti C, Tirelli F, Guariento C, et al. Impact of guidelines implementation on empiric antibiotic treatment for pediatric uncomplicated osteomyelitis and septic arthritis over a ten-year period: Results of the ELECTRIC study (ostEomyeLitis and sEptiC arThritis tReatment in children). Front Pediatr. 2023;11:1135319. Published 2023 Feb 23. doi:10.3389/fped.2023.1135319
151. Molloy L, McGrath E, Thomas R, Kaye KS, Rybak MJ. Acceptance of Pharmacist-Driven Antimicrobial Stewardship Recommendations With Differing Levels of Physician Involvement in a Children's Hospital. Clin Pediatr (Phila). 2017;56(8):744-751. doi:10.1177/0009922816678598
152. Mrosak J, Kandaswamy S, Stokes C, et al. The influence of integrating clinical practice guideline order bundles into a general admission order set on guideline adoption. JAMIA Open. 2021;4(4):ooab087. Published 2021 Oct 8. doi:10.1093/jamiaopen/ooab087
153. Muller MR, Mahadeo AM, Mayne JP, et al. Decreased Antibiotic Exposure for Suspected Early-Onset Sepsis in the Neonatal Intensive Care Unit Through Implementation of an Antimicrobial Time-out. J Pediatr Pharmacol Ther. 2022;27(8):746-749. doi:10.5863/1551-6776-27.8.746
154. Murni IK, Duke T, Kinney S, Daley AJ, Soenarto Y. Reducing hospital-acquired infections and improving the rational use of antibiotics in a developing country: an effectiveness study. Arch Dis Child. 2015;100(5):454-459. doi:10.1136/archdischild-2014-307297
155. Murni IK, Duke T, Kinney S, et al. Multifaceted interventions for healthcare-associated infections and rational use of antibiotics in a low-to-middle-income country: Can they be sustained?. PLoS One. 2020;15(6):e0234233. Published 2020 Jun 16. doi:10.1371/journal.pone.0234233
156. Newland JG, Stach LM, De Lurgio SA, et al. Impact of a Prospective-Audit-With-Feedback Antimicrobial Stewardship Program at a Children's Hospital. J Pediatric Infect Dis Soc. 2012;1(3):179-186. doi:10.1093/jpids/pis054
157. Newman RE, Hedican EB, Herigon JC, Williams DD, Williams AR, Newland JG. Impact of a guideline on management of children hospitalized with community-acquired pneumonia. Pediatrics. 2012;129(3):e597-e604. doi:10.1542/peds.2011-1533
158. Nguyen-Ha PT, Howrie D, Crowley K, et al. A Quality Assessment of a Collaborative Model of a Pediatric Antimicrobial Stewardship Program. Pediatrics. 2016;137(5):e20150316. doi:10.1542/peds.2015-0316
159. Nzegwu NI, Rychalsky MR, Nallu LA, et al. Implementation of an Antimicrobial Stewardship Program in a Neonatal Intensive Care Unit. Infect Control Hosp Epidemiol. 2017;38(10):1137-1143. doi:10.1017/ice.2017.151
160. Okado C, Teramae T. Antibiotic Practice Change to Curtail Linezolid Use in Pediatric Hospitalized Patients in Hawai'i with Uncomplicated Skin and Soft Tissue Infections. Hawaii J Health Soc Welf. 2020;79(5 Suppl 1):87-90.
161. Oliveira da Silva BB, de Menezes FG, Silva M, Troster EJ. The impact of monitoring software on antimicrobial management in a pediatric intensive care unit. Am J Infect Control. 2022;50(1):92-98. doi:10.1016/j.ajic.2021.08.016
162. Olson J, Mehra S, Hersh AL, et al. Oral Step-Down Therapy With Levofloxacin for Febrile Neutropenia in Children With Cancer. J Pediatric Infect Dis Soc. 2021;10(1):27-33. doi:10.1093/jpids/piaa015
163. Otake S, Nakagawa Y, Ryu H, Oue T, Kasai M. How do we reduce acyclovir overuse? Impact of FilmArray meningitis/encephalitis panel tests for pediatric patients. J Infect Chemother. 2022;28(9):1261-1265. doi:10.1016/j.jiac.2022.05.005
164. Pace D, Mack SJ, Chan S, et al. Antimicrobial Stewardship in Neonates with Necrotizing Enterocolitis: A Quality Improvement Initiative. J Pediatr Surg. 2023;58(10):1982-1989. doi:10.1016/j.jpedsurg.2023.06.009
165. Pantoja A, Sveum S, Frost S, et al. New strategies to Reduce Unnecessary Antibiotic Use in the NICU: A Quality Improvement Initiative. Pediatr Qual Saf. 2023;8(3):e659. Published 2023 Jun 7. doi:10.1097/pq9.0000000000000659
166. Papastergiou P, Tsioutis C, Mendris M. Implementation of a hospital antimicrobial stewardship program to improve vancomycin use in Cyprus: Challenges and opportunity. Infect Prev Pract. 2022;4(4):100254. Published 2022 Oct 20. doi:10.1016/j.infpip.2022.100254
167. Parker SK, Hurst AL, Thurm C, et al. Anti-infective Acquisition Costs for a Stewardship Program: Getting to the Bottom Line. Clin Infect Dis. 2017;65(10):1632-1637. doi:10.1093/cid/cix631
168. Pauquet E, Coppry M, Sarlangue J, Rogues AM. Carbapenem stewardship program in a French university children's hospital. Arch Pediatr. 2021;28(8):621-625. doi:10.1016/j.arcped.2021.10.004
169. Pontello E, Favero V, Mainini N, et al. Neonatal Early Onset Sepsis: Impact of Kaiser Calculator in an Italian Tertiary Perinatal Center. Pediatr Infect Dis J. 2022;41(2):161-165. doi:10.1097/INF.0000000000003342
170. Putnam LR, Chang CM, Rogers NB, et al. Adherence to surgical antibiotic prophylaxis remains a challenge despite multifaceted interventions. Surgery. 2015;158(2):413-419. doi:10.1016/j.surg.2015.04.013
171. Ren Z, Yang S, Han J, et al. Reduction of antibiotic use and multi-drug resistance bacteria infection in neonates after improvement of antibiotics use strategy in a level 4 neonatal intensive care unit in southern China. Eur J Clin Microbiol Infect Dis. 2023;42(1):87-98. doi:10.1007/s10096-022-04522-4
172. Renk H, Sarmisak E, Spott C, Kumpf M, Hofbeck M, Hölzl F. Antibiotic stewardship in the PICU: Impact of ward rounds led by paediatric infectious diseases specialists on antibiotic consumption. Sci Rep. 2020;10(1):8826. Published 2020 Jun 1. doi:10.1038/s41598-020-65671-0
173. Ross RK, Beus JM, Metjian TA, et al. Safety of Automatic End Dates for Antimicrobial Orders to Facilitate Stewardship. Infect Control Hosp Epidemiol. 2016;37(8):974-978. doi:10.1017/ice.2016.103
174. Rungsitsathian K, Wacharachaisurapol N, Nakaranurack C, et al. Acceptance and outcome of interventions in a meropenem de-escalation antimicrobial stewardship program in pediatrics. Pediatr Int. 2021;63(12):1458-1465. doi:10.1111/ped.14703
175. Ruvinsky S, Mónaco A, Pérez G, et al. Effectiveness of a program to improve antibiotic use in children hospitalized in a children's tertiary care facility in Argentina. Arch Argent Pediatr. 2014;112(2):124-131. doi:10.5546/aap.2014.eng.124
176. Salau HD, Orchard A, Stacey S, Varughese S, Johnston D, Khan R. Antibiotic usage in a South African paediatric medical ward following the introduction of an antibiotic prescription chart. Pan Afr Med J. 2023;45:26. Published 2023 May 8. doi:10.11604/pamj.2023.45.26.36548
177. Same RG, Amoah J, Hsu AJ, et al. The Association of Antibiotic Duration With Successful Treatment of Community-Acquired Pneumonia in Children. J Pediatric Infect Dis Soc. 2021;10(3):267-273. doi:10.1093/jpids/piaa055
178. Savage TJ, Rao S, Joerger J, Ozonoff A, McAdam AJ, Sandora TJ. Predictive Value of Direct Disk Diffusion Testing from Positive Blood Cultures in a Children's Hospital and Its Utility in Antimicrobial Stewardship. J Clin Microbiol. 2021;59(6):e02445-20. Published 2021 May 19. doi:10.1128/JCM.02445-20
179. Saw C, Kulasekaran K, Fernando DT, et al. Retrospective cohort study of neonatal early onset of sepsis and the role of the EOS calculator in a level II nursery. Pediatr Neonatol. 2021;62(5):512-521. doi:10.1016/j.pedneo.2021.05.005
180. Schwenk HT, Kruger JF, Sacks LD, Wood MS, Qureshi L, Bio LL. Use of Prospective Audit and Feedback to Reduce Antibiotic Exposure in a Pediatric Cardiac ICU. Pediatr Crit Care Med. 2021;22(3):e224-e232. doi:10.1097/PCC.0000000000002608
181. Scott PA, Lai M, Inglis GDT, Davies MW. Neonatal early-onset sepsis calculator safety in an Australian tertiary perinatal centre. J Paediatr Child Health. 2022;58(5):863-867. doi:10.1111/jpc.15860
182. Seah XF, Ong YL, Tan SW, et al. Impact of an antimicrobial stewardship program on the use of carbapenems in a tertiary women's and children's hospital, Singapore. Pharmacotherapy. 2014;34(11):1141-1150. doi:10.1002/phar.1490
183. Seddik TB, Rabsatt LA, Mueller C, et al. Reducing Piperacillin and Tazobactam Use for Pediatric Perforated Appendicitis. J Surg Res. 2021;260:141-148. doi:10.1016/j.jss.2020.11.067
184. Sick AC, Lehmann CU, Tamma PD, Lee CK, Agwu AL. Sustained savings from a longitudinal cost analysis of an internet-based preapproval antimicrobial stewardship program. Infect Control Hosp Epidemiol. 2013;34(6):573-580. doi:10.1086/670625
185. Sick-Samuels AC, Booth LD, Milstone AM, Schumacher C, Bergmann J, Stockwell DC. A Novel Comprehensive Algorithm for Evaluation of PICU Patients With New Fever or Instability. Pediatr Crit Care Med. 2023;24(8):670-680. doi:10.1097/PCC.0000000000003256
186. Simó S, Velasco-Arnaiz E, Ríos-Barnés M, et al. Effects of a Paediatric Antimicrobial Stewardship Program on Antimicrobial Use and Quality of Prescriptions in Patients with Appendix-Related Intraabdominal Infections. Antibiotics (Basel). 2020;10(1):5. Published 2020 Dec 23. doi:10.3390/antibiotics10010005
187. Smith MJ, Kong M, Cambon A, Woods CR. Effectiveness of antimicrobial guidelines for community-acquired pneumonia in children. Pediatrics. 2012;129(5):e1326-e1333. doi:10.1542/peds.2011-2412
188. So JP, Aleem IS, Tsang DS, Matlow AG, Wright JG; SickKids Surgical Site Infection Task Force. Increasing Compliance With an Antibiotic Prophylaxis Guideline to Prevent Pediatric Surgical Site Infection: Before and After Study. Ann Surg. 2015;262(2):403-408. doi:10.1097/SLA.0000000000000934
189. Stocker M, Hop WC, van Rossum AM. Neonatal Procalcitonin Intervention Study (NeoPInS): Effect of Procalcitonin-guided decision making on duration of antibiotic therapy in suspected neonatal early-onset sepsis: A multi-centre randomized superiority and non-inferiority Intervention Study. BMC Pediatr. 2010;10:89. Published 2010 Dec 8. doi:10.1186/1471-2431-10-89
190. Stocker M, Ferrao E, Banya W, Cheong J, Macrae D, Furck A. Antibiotic surveillance on a paediatric intensive care unit: easy attainable strategy at low costs and resources. BMC Pediatr. 2012;12:196. Published 2012 Dec 21. doi:10.1186/1471-2431-12-196
191. Stocker M, van Herk W, El Helou S, et al. Procalcitonin-guided decision making for duration of antibiotic therapy in neonates with suspected early-onset sepsis: a multicentre, randomised controlled trial (NeoPIns). Lancet. 2017;390(10097):871-881. doi:10.1016/S0140-6736(17)31444-7
192. Taylor M, Liechti S, Palazzi D. Intermittent Education and Audit and Feedback Reduce Inappropriate Prescribing of Oral Third-Generation Cephalosporins for Pediatric Upper Respiratory Tract Infections. Jt Comm J Qual Patient Saf. 2021;47(4):250-257. doi:10.1016/j.jcjq.2020.12.003
193. Thampi N, Shah PS, Nelson S, et al. Prospective audit and feedback on antibiotic use in neonatal intensive care: a retrospective cohort study. BMC Pediatr. 2019;19(1):105. Published 2019 Apr 11. doi:10.1186/s12887-019-1481-z
194. Ting JY, Paquette V, Ng K, et al. Reduction of Inappropriate Antimicrobial Prescriptions in a Tertiary Neonatal Intensive Care Unit After Antimicrobial Stewardship Care Bundle Implementation. Pediatr Infect Dis J. 2019;38(1):54-59. doi:10.1097/INF.0000000000002039
195. Tolia VN, Desai S, Qin H, et al. Implementation of an Automatic Stop Order and Initial Antibiotic Exposure in Very Low Birth Weight Infants. Am J Perinatol. 2017;34(2):105-110. doi:10.1055/s-0036-1584522
196. Turner RB, Valcarlos E, Loeffler AM, Gilbert M, Chan D. Impact of an Antimicrobial Stewardship Program on Antibiotic Use at a Nonfreestanding Children's Hospital. J Pediatric Infect Dis Soc. 2017;6(3):e36-e40. doi:10.1093/jpids/piw059
197. Uhl BD, Boutzoukas A, Gallup N, et al. Increasing Adherence to Acute Otitis Media Treatment Duration Guidelines using a Quality Improvement Approach. Pediatr Qual Saf. 2021;6(6):e501. Published 2021 Dec 15. doi:10.1097/pq9.0000000000000501
198. Velasco-Arnaiz E, Simó-Nebot S, Ríos-Barnés M, et al. Benefits of a Pediatric Antimicrobial Stewardship Program in Antimicrobial Use and Quality of Prescriptions in a Referral Children's Hospital. J Pediatr. 2020;225:222-230.e1. doi:10.1016/j.jpeds.2020.06.008
199. Verma A, Vimalesvaran S, Dhawan A. Epidemiology, Risk Factors and Outcome Due to Multidrug Resistant Organisms in Paediatric Liver Transplant Patients in the Era of Antimicrobial Stewardship and Screening. Antibiotics (Basel). 2022;11(3):387. Published 2022 Mar 15. doi:10.3390/antibiotics11030387
200. Villanueva P, Freyne B, Hickey L, Carr J, Bryant PA. Impact of an antimicrobial stewardship intervention in neonatal intensive care: Recommendations and implementation. J Paediatr Child Health. 2021;57(8):1208-1214. doi:10.1111/jpc.15427
201. Villaverde S, Caro JM, Domínguez-Rodríguez S, et al. PACTA-Ped: Antimicrobial stewardship programme in a tertiary care hospital in Spain. An Pediatr (Engl Ed). 2023;99(5):312-320. doi:10.1016/j.anpede.2023.09.012
202. Vyas DP, Quinones-Cardona V, Gilfillan MA, Young ME, Pough KA, Carey AJ. Reduction of unnecessary antibiotic days in a level IV neonatal intensive care unit. Antimicrob Steward Healthc Epidemiol. 2022;2(1):e50. Published 2022 Mar 28. doi:10.1017/ash.2022.33
203. Walker S, Datta A, Massoumi RL, Gross ER, Uhing M, Arca MJ. Antibiotic stewardship in the newborn surgical patient: A quality improvement project in the neonatal intensive care unit. Surgery. 2017;162(6):1295-1303. doi:10.1016/j.surg.2017.07.021
204. Wattier RL, Levy ER, Sabnis AJ, Dvorak CC, Auerbach AD. Reducing Second Gram-Negative Antibiotic Therapy on Pediatric Oncology and Hematopoietic Stem Cell Transplantation Services. Infect Control Hosp Epidemiol. 2017;38(9):1039-1047. doi:10.1017/ice.2017.118
205. Webber EC, Warhurst HM, Smith SS, Cox EG, Crumby AS, Nichols KR. Conversion of a single-facility pediatric antimicrobial stewardship program to multi-facility application with computerized provider order entry and clinical decision support. Appl Clin Inform. 2013;4(4):556-568. Published 2013 Nov 27. doi:10.4338/ACI-2013-07-RA-0054
206. Willis ZI, Gillon J, Xu M, Slaughter JC, Di Pentima MC. Reducing Antimicrobial Use in an Academic Pediatric Institution: Evaluation of the Effectiveness of a Prospective Audit With Real-Time Feedback. J Pediatric Infect Dis Soc. 2017;6(4):339-345. doi:10.1093/jpids/piw054
207. Woods-Hill CZ, Colantuoni EA, Koontz DW, et al. Association of Diagnostic Stewardship for Blood Cultures in Critically Ill Children With Culture Rates, Antibiotic Use, and Patient Outcomes: Results of the Bright STAR Collaborative. JAMA Pediatr. 2022;176(7):690-698. doi:10.1001/jamapediatrics.2022.1024
208. Wright MR, Gillon J, Katz SE, Banerjee R. Requiring durations of therapy at the time of antibiotic order entry reduces antibiotic use. Antimicrob Steward Healthc Epidemiol. 2025;5(1):e45. Published 2025 Feb 12. doi:10.1017/ash.2025.20
209. Wu G, Wu G, Wu S, Wu H. Comparison of Procalcitonin Guidance-Administered Antibiotics with Standard Guidelines on Antibiotic Therapy in Children with Lower Respiratory Tract Infections: A Retrospective Study in China. Med Princ Pract. 2017;26(4):316-320. doi:10.1159/000477936
210. Yonts AB, O'Neill LB, Magyar MA, Bozzella MJ. Multidisciplinary Initiative to Increase Guideline-concordant Antibiotic Prescription at Discharge for Hospitalized Children with Uncomplicated Community-acquired Pneumonia. Pediatr Qual Saf. 2023;8(6):e711. Published 2023 Dec 12. doi:10.1097/pq9.0000000000000711
211. Yoshida K, Hatachi T, Okamoto Y, et al. Application of Multiplex Polymerase Chain Reaction for Pathogen Identification and Antibiotic Use in Children With Respiratory Infections in a PICU. Pediatr Crit Care Med. 2021;22(12):e644-e648. doi:10.1097/PCC.0000000000002794
212. Yoshimura J, Yamakawa K, Ohta Y, et al. Effect of Gram Stain-Guided Initial Antibiotic Therapy on Clinical Response in Patients With Ventilator-Associated Pneumonia: The GRACE-VAP Randomized Clinical Trial [published correction appears in JAMA Netw Open. 2022 Oct 03;5(10):e2240335. doi: 10.1001/jamanetworkopen.2022.40335.]. JAMA Netw Open. 2022;5(4):e226136. Published 2022 Apr 1. doi:10.1001/jamanetworkopen.2022.6136
213. Yu D, Stach L, Newland JG, Selvarangan R, Goldman J. Integrating a Rapid Diagnostic Test and Antimicrobial Stewardship: Optimizing Discharge Antibiotics in Skin and Soft Tissue Infections. Pediatr Infect Dis J. 2016;35(12):1362-1364. doi:10.1097/INF.0000000000001332
214. Zihlmann-Ji J, Braun C, Buettcher M, Hodel M, Lehnick D, Stocker M. Reduction of Duration of Antibiotic Therapy for Suspected Early-Onset Sepsis in Late-Preterm and Term Newborns After Implementation of a Procalcitonin-Guided Algorithm: A Population-Based Study in Central Switzerland. Front Pediatr. 2021;9:702133. Published 2021 Jul 22. doi:10.3389/fped.2021.702133
215. Gunnlaugsdottir MR, Linnet K, Jonsson JS, Blondal AB. Encouraging rational antibiotic prescribing behaviour in primary care - prescribing practice among children aged 0-4 years 2016-2018: an observational study. Scand J Prim Health Care. 2021;39(3):373-381. doi:10.1080/02813432.2021.1958506
216. Machnes MD, Cohen HA, Gerstein M, et al. Antibiotic Stewardship for Community-Acquired Pediatric Pharyngitis: A Pre-Post Intervention Study. Isr Med Assoc J. 2023;25(7):500-504.
217. Shapiro DJ, Hall M, Lipsett SC, et al. Short- Versus Prolonged-Duration Antibiotics for Outpatient Pneumonia in Children. J Pediatr. 2021;234:205-211.e1. doi:10.1016/j.jpeds.2021.03.017
218. Al-Tawfiq JA, Alawami AH. A multifaceted approach to decrease inappropriate antibiotic use in a pediatric outpatient clinic. Ann Thorac Med. 2017;12(1):51-54. doi:10.4103/1817-1737.197779
219. Blair PS, Young G, Clement C, et al. Multi-faceted intervention to improve management of antibiotics for children presenting to primary care with acute cough and respiratory tract infection (CHICO): efficient cluster randomised controlled trial. BMJ. 2023;381:e072488. Published 2023 Apr 26. doi:10.1136/bmj-2022-072488
220. Bourgeois FC, Linder J, Johnson SA, Co JP, Fiskio J, Ferris TG. Impact of a computerized template on antibiotic prescribing for acute respiratory infections in children and adolescents. Clin Pediatr (Phila). 2010;49(10):976-983. doi:10.1177/0009922810373649
221. Clegg HW, Ezzo SJ, Flett KB, Anderson WE. Improving antibiotic prescribing for pediatric acute respiratory tract infections: A cluster randomized trial to evaluate individual versus clinic feedback. Antimicrob Steward Healthc Epidemiol. 2021;1(1):e43. Published 2021 Nov 3. doi:10.1017/ash.2021.212
222. Clegg HW, Bean RA, Ezzo SJ, Hoth AN, Sheedy DJ, Anderson WE. Impact of Education and Peer Comparison on Antibiotic Prescribing for Pediatric Respiratory Tract Infections. Pediatr Qual Saf. 2019;4(4):e195. Published 2019 Jul 29. doi:10.1097/pq9.0000000000000195
223. Cohen HA, Gerstein M, Loewenberg Weisband Y, et al. Pediatric Antibiotic Stewardship for Community-Acquired Pneumonia: A Pre-Post Intervention Study. Clin Pediatr (Phila). 2022;61(11):795-801. doi:10.1177/00099228221102827
224. Di Mario S, Gagliotti C, Buttazzi R, et al. Observational pre-post study showed that a quality improvement project reduced paediatric antibiotic prescribing rates in primary care. Acta Paediatr. 2018;107(10):1805-1809. doi:10.1111/apa.14381
225. Diaz MCG, Handy LK, Crutchfield JH Jr, Cadilla A, Hossain J, Werk LN. Impact of a Personalized Audit and Feedback Intervention on Antibiotic Prescribing Practices for Outpatient Pediatric Community-Acquired Pneumonia. Clin Pediatr (Phila). 2020;59(11):988-994. doi:10.1177/0009922820928054
226. Fiks AG, Zhang P, Localio AR, et al. Adoption of electronic medical record-based decision support for otitis media in children. Health Serv Res. 2015;50(2):489-513. doi:10.1111/1475-6773.12240
227. Finkelstein JA, Huang SS, Kleinman K, et al. Impact of a 16-community trial to promote judicious antibiotic use in Massachusetts. Pediatrics. 2008;121(1):e15-e23. doi:10.1542/peds.2007-0819
228. Forrest CB, Fiks AG, Bailey LC, et al. Improving adherence to otitis media guidelines with clinical decision support and physician feedback. Pediatrics. 2013;131(4):e1071-e1081. doi:10.1542/peds.2012-1988
229. Francis NA, Butler CC, Hood K, Simpson S, Wood F, Nuttall J. Effect of using an interactive booklet about childhood respiratory tract infections in primary care consultations on reconsulting and antibiotic prescribing: a cluster randomised controlled trial. BMJ. 2009;339:b2885. Published 2009 Jul 29. doi:10.1136/bmj.b2885
230. Frost HM, Monti JD, Andersen LM, et al. Improving Delayed Antibiotic Prescribing for Acute Otitis Media. Pediatrics. 2021;147(6):e2020026062. doi:10.1542/peds.2020-026062
231. Gagliotti C, Buttazzi R, Di Mario S, Morsillo F, Moro ML. A regionwide intervention to promote appropriate antibiotic use in children reversed trends in erythromycin resistance to Streptococcus pyogenes. Acta Paediatr. 2015;104(9):e422-e424. doi:10.1111/apa.13072
232. Gulliford MC, Prevost AT, Charlton J, et al. Effectiveness and safety of electronically delivered prescribing feedback and decision support on antibiotic use for respiratory illness in primary care: REDUCE cluster randomised trial. BMJ. 2019;364:l236. Published 2019 Feb 12. doi:10.1136/bmj.l236
233. Hersh AL, Olson J, Stockmann C, et al. Impact of Antimicrobial Stewardship for Pediatric Outpatient Parenteral Antibiotic Therapy. J Pediatric Infect Dis Soc. 2018;7(2):e34-e36. doi:10.1093/jpids/pix038
234. Hürlimann D, Limacher A, Schabel M, et al. Improvement of antibiotic prescription in outpatient care: a cluster-randomized intervention study using a sentinel surveillance network of physicians. J Antimicrob Chemother. 2015;70(2):602-608. doi:10.1093/jac/dku394
235. Huynh J, Hodgson KA, Boyce S, Ibrahim LF, Bryant PA. Impact of expanding a paediatric OPAT programme with an antimicrobial stewardship intervention. Arch Dis Child. 2020;105(12):1220-1228. doi:10.1136/archdischild-2019-318091
236. Iwamoto N, Morisaki N, Uda K, et al. Change in use of pediatric oral antibiotics in Japan, pre- and post-implementation of an antimicrobial resistance action plan. Pediatr Int. 2022;64(1):e15197. doi:10.1111/ped.15197
237. Jindrak V, Marek J, Vanis V, et al. Improvements in antibiotic prescribing by community paediatricians in the Czech Republic. Euro Surveill. 2008;13(46):19040. Published 2008 Nov 13.
238. Karas DR, Upadhyayula S, Love A, Bigham MT. Utilizing Clinical Decision Support in the Treatment of Urinary Tract Infection across a Large Pediatric Primary Care Network. Pediatr Qual Saf. 2023;8(3):e655. Published 2023 May 22. doi:10.1097/pq9.0000000000000655
239. Katz SE, Spencer P, Cates J, Harnack L, Xu M, Banerjee R. Improvements in appropriate ambulatory antibiotic prescribing using a bundled antibiotic stewardship intervention in general pediatrics practices. Infect Control Hosp Epidemiol. 2022;43(12):1894-1900. doi:10.1017/ice.2021.534
240. Lemiengre MB, Verbakel JY, Colman R, et al. Point-of-care CRP matters: normal CRP levels reduce immediate antibiotic prescribing for acutely ill children in primary care: a cluster randomized controlled trial. Scand J Prim Health Care. 2018;36(4):423-436. doi:10.1080/02813432.2018.1529900
241. Mainous AG 3rd, Lambourne CA, Nietert PJ. Impact of a clinical decision support system on antibiotic prescribing for acute respiratory infections in primary care: quasi-experimental trial. J Am Med Inform Assoc. 2013;20(2):317-324. doi:10.1136/amiajnl-2011-000701
242. Norton LE, Lee BR, Harte L, et al. Improving Guideline-Based Streptococcal Pharyngitis Testing: A Quality Improvement Initiative. Pediatrics. 2018;142(1):e20172033. doi:10.1542/peds.2017-2033
243. Pagano F, Amato C, De Marco G, et al. Reduction in broad-spectrum antimicrobial prescriptions by primary care pediatricians following a multifaceted antimicrobial stewardship program. Front Pediatr. 2023;10:1070325. Published 2023 Jan 6. doi:10.3389/fped.2022.1070325
244. Papaevangelou V, Rousounides A, Hadjipanagis A, Katsioulis A, Theodoridou M, Hadjichristodoulou C. Decrease of antibiotic consumption in children with upper respiratory tract infections after implementation of an intervention program in Cyprus. Antimicrob Agents Chemother. 2012;56(3):1658-1661. doi:10.1128/AAC.05969-11
245. Puzz L, Plauche EA, Cretella DA, Harrison VA, Wingler MJB. Evaluation of a Pediatric Community-Acquired Pneumonia Antimicrobial Stewardship Intervention at an Academic Medical Center. Antibiotics (Basel). 2023;12(4):780. Published 2023 Apr 19. doi:10.3390/antibiotics12040780
246. Ray KN, Martin JM, Wolfson D, et al. Antibiotic Prescribing for Acute Respiratory Tract Infections During Telemedicine Visits Within a Pediatric Primary Care Network. *Acad Pediatr*. 2021;21(7):1239-1243. doi:10.1016/j.acap.2021.03.008
247. Regev-Yochay G, Raz M, Dagan R, et al. Reduction in antibiotic use following a cluster randomized controlled multifaceted intervention: the Israeli judicious antibiotic prescription study. *Clin Infect Dis*. 2011;53(1):33-41. doi:10.1093/cid/cir272
248. Stille CJ, Rifas-Shiman SL, Kleinman K, Kotch JB, Finkelstein JA. Physician responses to a community-level trial promoting judicious antibiotic use. *Ann Fam Med*. 2008;6(3):206-212. doi:10.1370/afm.839
249. Torres FA, Pasarelli I, Cutri A, Ossorio MF, Ferrero F. Impact assessment of a decision rule for using antibiotics in pneumonia: a randomized trial. Pediatr Pulmonol. 2014;49(7):701-706. doi:10.1002/ppul.22849
250. Trinh NTH, Bruckner TA, Lemaitre M, et al. Association between National Treatment Guidelines for Upper Respiratory Tract Infections and Outpatient Pediatric Antibiotic Use in France: An Interrupted Time-Series Analysis. J Pediatr. 2020;216:88-94.e4. doi:10.1016/j.jpeds.2019.09.017
251. Wei X, Zhang Z, Walley JD, et al. Effect of a training and educational intervention for physicians and caregivers on antibiotic prescribing for upper respiratory tract infections in children at primary care facilities in rural China: a cluster-randomised controlled trial [published correction appears in Lancet Glob Health. 2018 Jan;6(1):e37. doi: 10.1016/S2214-109X(17)30447-3.]. Lancet Glob Health. 2017;5(12):e1258-e1267. doi:10.1016/S2214-109X(17)30383-2
252. Zhang Z, Dawkins B, Hicks JP, et al. Cost-effectiveness analysis of a multi-dimensional intervention to reduce inappropriate antibiotic prescribing for children with upper respiratory tract infections in China. *Trop Med Int Health*. 2018;23(10):1092-1100. doi:10.1111/tmi.13132
